# Reversible [4 + 1] Cycloaddition of Arenes by a “Naked” Acyclic Aluminyl Compound

**DOI:** 10.1021/jacs.4c00376

**Published:** 2024-04-16

**Authors:** Debotra Sarkar, Petra Vasko, Aisling F. Roper, Agamemnon E. Crumpton, Matthew M. D. Roy, Liam P. Griffin, Charlotte Bogle, Simon Aldridge

**Affiliations:** † Inorganic Chemistry Laboratory, Department of Chemistry, University of Oxford, South Parks Road, Oxford OX1 3QR, U.K.; ‡ Department of Chemistry, University of Helsinki, A.I. Virtasen Aukio 1, P.O. Box 55, Helsinki FI-00014, Finland

## Abstract

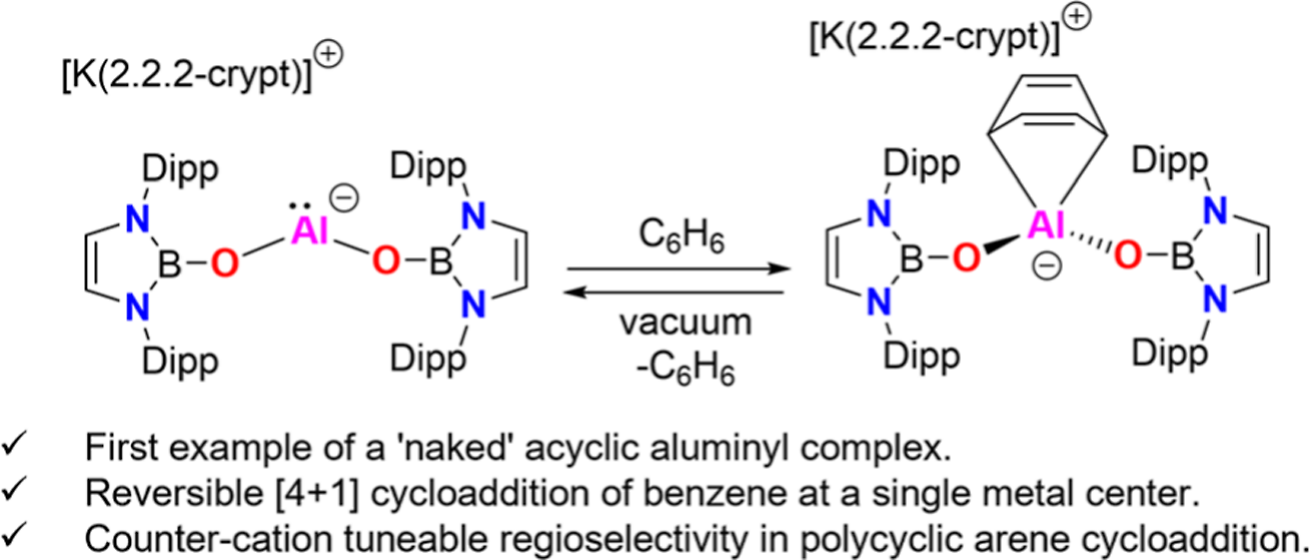

The large steric profile of the N-heterocyclic boryloxy ligand, –OB(NDippCH)_2_, and its ability to stabilize the metal-centered HOMO, are exploited in the synthesis of the first example of a “naked” acyclic aluminyl complex, [K(2.2.2-crypt)][Al{OB(NDippCH)_2_}_2_]. This system, which is formed by substitution at Al^I^ (rather than reduction of Al^III^), represents the first O-ligated aluminyl compound and is shown to be capable of hitherto unprecedented reversible single-site [4 + 1] cycloaddition of benzene. This chemistry and the unusual regioselectivity of the related cycloaddition of anthracene are shown to be highly dependent on the availability (or otherwise) of the K^+^ countercation.

## Introduction

The activation of small molecules using compounds of abundant main group elements represents a challenging yet attractive chemical endeavor.^1,2^ A number of powerful new paradigms for the binding and/or transformation of industrially relevant substrates have arisen in recent years, including carbenes (and their heavier metallylene counterparts),^3,4^ frustrated Lewis pairs,^5^ and dimetallynes—the heavier Group 14 analogues of alkynes.^6^ In a more limited number of cases, such systems even allow for the reversible activation of small molecules such as H_2_, alkenes, etc.^1,2,7−9^

Aluminum, the most abundant metal in the Earth’s crust, has gained attention on the basis of novel patterns of reactivity displayed by its compounds in the formal +I oxidation state.^10,11^ These include not only neutral compounds such as [Cp*Al]_4_ and {HC(RCDippN)_2_}Al (R = Me, ^*t*^Bu; Dipp = 2,6-^*i*^Pr_2_C_6_H_3_),^12,13^ which display significant capabilities in the oxidative addition of (for example) E–H bonds (E = H, B, Si, O, P, etc.),^14^ but also (more recently) anionic aluminyl compounds, [AlX_2_]^−^, which typically feature more nucleophilic metal centers. Such systems have further pushed the boundaries of small molecule activation by p-block systems; for instance, [K(NON)Al]_2_ [NON = 4,5-bis(2,6-di-isopropylanilido)-2,7-di-*tert*-butyl-9,9 dimethylxanthene)] affect single-site C–H oxidative addition of benzene (the first example for a main-group metal),^15^ while its monomeric counterpart [K(2.2.2-crypt)][(NON)Al] is capable of reversible C–C bond activation of the same substrate.^16^ Exploiting the high degree of metal-centered nucleophilicity of aluminyl reagents, a range of other small molecules (H_2_, CO_2_, N_2_O, P_4_, etc.) have also been activated, leading to the formation of reactive Al–E bonds (E = H, O, N, P, etc.).^10,11^

**Figure 1:**
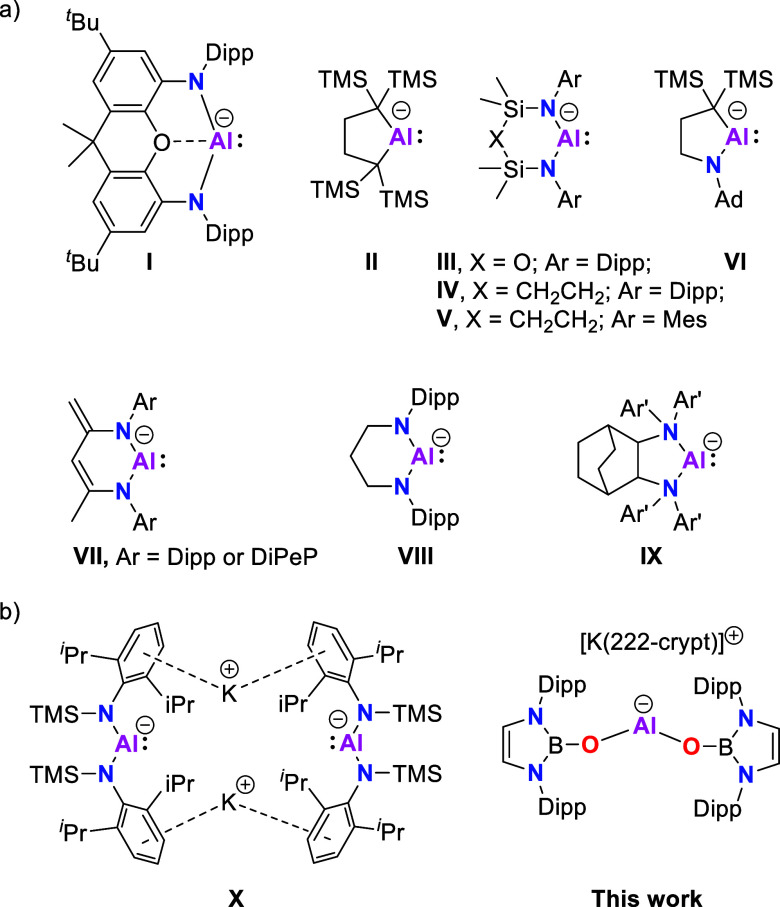
(a) Cyclic aluminyl compounds and (b) acyclic aluminyl compounds [DIPeP = 2,6-{Et_2_(H)C}_2_C_6_H_3_, Ar′ = 3,5-^t^Bu_2_C_6_H_3_].

Typically, aluminyl systems have been stabilized by N/N,^17−23^ N/C,^24,25^ or C/C-based^26^ dianionic donor sets within a chelating scaffold, resulting in an overall cyclic structure (e.g., **I–IX**; Figure 1a).^17^ Acyclic aluminyl compounds offer potentially greater flexibility in terms of the X–Al–X angle and would therefore be expected (by analogies previously drawn with related carbene/metallylene systems)^10,27^ to offer greater scope for variation of the HOMO–LUMO energy separation (and hence reactivity). The energy of the HOMO (typically a σ-symmetry lone pair at the carbene/metallylene center) is known to be strongly affected by the angle between the two X substituents, based on its influence on the s/p orbital contributions.^28^

Recently, Liptrot, Hicks, and co-workers reported an acyclic bis-amido aluminyl compound K_2_[Al{N(Dipp)SiMe_3_}_2_]_2_, **X** (Figure 1b), which was shown to exist as a dimer in solution and in the solid state.^29^ By contrast, a simple monomeric “naked” [AlX_2_]^−^ system of this type, free from stabilizing interactions with the countercation, would be expected to be extremely reactive, and such compounds have not yet been reported. In our pursuit of a such a system, we hypothesized that the use of highly electronegative (e.g., O-derived) X-substituents might help stabilize the Al-centered lone pair in the absence of a closely bound countercation.^28^ That said, there are no previous reports of systems of this type bearing alkoxy or related substituents, and even among the more heavily populated carbene family of compounds, few isolable examples of alkoxy-carbenes have been reported.^28,30,31^ Based on our previous work with heavier group 14 systems, however, we hypothesized that the N-heterocyclic boryloxy (NHBO) ligand might offer desirable characteristics, namely, (i) a high degree of steric bulk inherent in the single O-substituent; and (ii) Al lone pair stabilization by the highly electronegative α-substituents. For example, by means of comparison, the acyclic silylene Si{OB(NDippCH)_2_}_2_, which is isoelectronic with [Al{OB(NDippCH)_2_}_2_]^−^, features a very wide HOMO–LUMO gap (>5.4 eV), primarily on the basis of a very low-lying Si-centered lone pair.^32^

## Results and Discussion

Previously reported methods for the synthesis of aluminyl compounds typically involve the reaction of an Al^III^ halide with an alkali metal reductant.^10,11^ In the case of [Al{OB(NDippCH)_2_}_2_]^−^ however, related chemistry proved unsuccessful, generating intractable mixtures of products. With this in mind, we targeted alternative metathesis processes, exploiting ready-made Al^I^ precursors. We have previously employed the cyclopentadienyl anion as a leaving group^33^ and envisioned that the Cp* substituents in (Cp*Al)_4_ might undergo displacement by the NHBO ligand, driven by the low solubility of KCp*, and the oxophilicity of aluminum.^12,34−36^

**Scheme 1:**
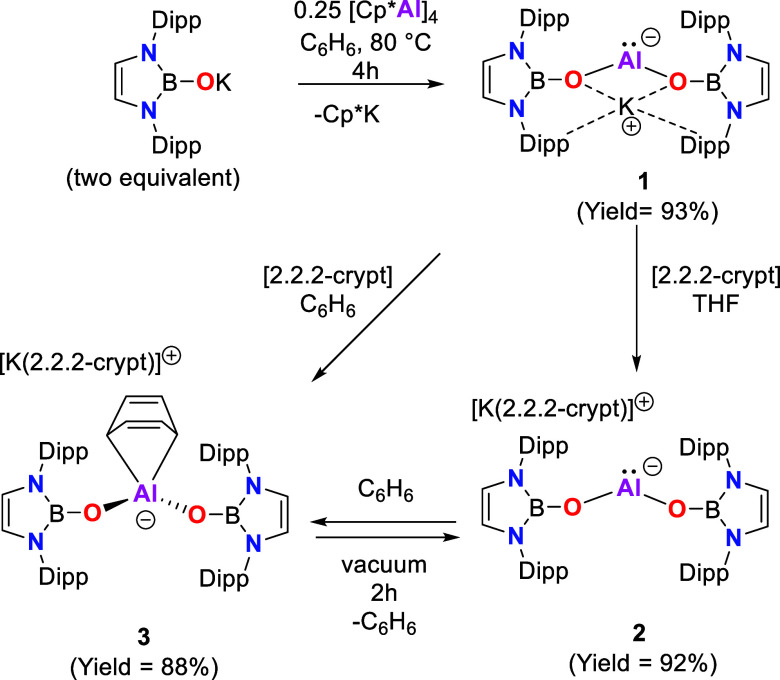
Syntheses of Compounds **1–3**

**Figure 2:**
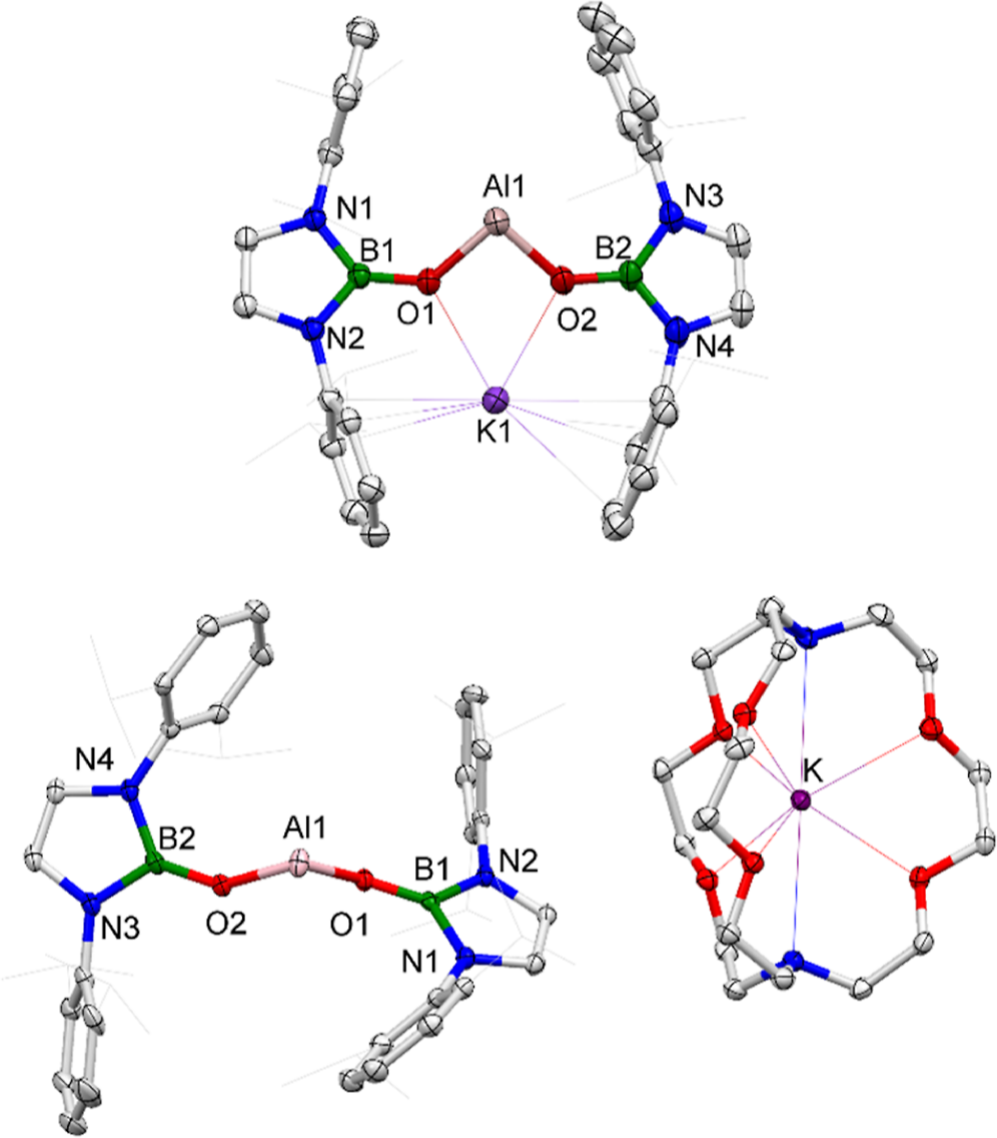
Molecular structures of **1** (upper) and **2** (lower) in the solid state as determined by X-ray crystallography (ellipsoids set at 35% probability level; H atoms omitted and ^*i*^Pr groups shown in the wireframe format for clarity). Key bond lengths (Å) and angles (deg): **1**: B1–O1 1.355(4), B2–O2 1.366(4), Al1–O1 1.817(2), Al1–O2 1.797(3); O1–Al1–O2 92.3(1); **2**: B1–O1 1.320(3), B2–O2 1.330(3), Al1–O1 1.787(2), Al1–O2 1.784(2); and O1–Al1–O2 100.0(9).

Treatment of (Cp*Al)_4_ with eight equivalents of K[OB(NDippCH)_2_] in benzene at 80 °C results in the elimination of KCp* and the formation of room-temperature stable bis-boryloxy aluminyl compound [KAl{OB(NDippCH)_2_}_2_], **1** (Scheme 1). **1** can be isolated in 93% yield as a pale-yellow powder and has been characterized by multinuclear NMR spectroscopy and X-ray diffraction. Its molecular structure in the solid state (Figure 2) reveals a two-coordinate aluminum center bound to two NHBO ligands, with the K^+^ cation encapsulated between the two oxygen atoms, to generate a four-membered AlO_2_K core. Notably, the bond angle at aluminum (∠O1–Al1–O2, 92.3(1)°) is markedly narrower than that observed in the N-ligated aluminyl dimer **X** (116.61(17) and 116.33(16)°).^29^

The frontier orbital energies (and hence reactivity) of carbene-like species are known to be strongly dependent on the angle at the central metal atom.^27^ With this in mind, we sought to remove the K^+^ ion from within the ligand scaffold of **1** using the sequestering agent 2.2.2-cryptand, to generate the “naked” acyclic aluminyl anion [Al{OB(NDippCH)_2_}_2_]^−^. The reaction between **1** and an equimolar amount of 2.2.2-cryptand in THF yields [K(2.2.2-crypt)] [Al{OB(NDippCH)_2_}_2_] (**2**) as an orange-yellow powder in a high yield (Scheme 1). **2** is insoluble in hexane, pentane, and toluene and decomposes in haloarenes. Single crystals of **2**, however, could be obtained from a mesitylene solution of **1** and 2.2.2-cryptand. The solid-state structure (Figure 2) reveals that **2** consists of well-separated anionic/cationic components, with all K···Al contacts >9 Å. Examples of charge-separated aluminyl compounds, devoid of the stabilizing influence of the M···Al interaction, are very rare.^16,21,22,24^

The O1–Al1–O2 bond angle in **2** is significantly wider than that of **1** (100.0(9)° vs 92.2(1)°). However, DFT calculations carried out on both systems (at the PBE0-GD3BJ/Def2-TZVP level, using the full molecules in both cases) reveal remarkably similar (wide) energy separations between the aluminum-based lone pair and the orthogonal Al-centered p_π_ orbital, albeit with the orbital manifold lying significantly higher in the case of charge-separated **2** (cf. lone pair energies of −3.94 and −2.77 eV, respectively). The respective lone pair to p_π_ energy separations are 4.34 eV (419 kJ mol^–1^) for **1** and 4.29 eV (414 kJ mol^–1^) for **2**. These observations can be rationalized on the basis of two (aligned) effects caused by K^+^ abstraction: (i) elevation of the lone pair (from −3.94 to −2.77 eV) on the basis of both the wider O–Al–O angle, and electrostatic factors associated with diminished anion/cation contact; and (ii) elevation of the (antibonding) p_π_ orbital (from +0.40 to +1.52 eV) due to more efficient O-to-Al π donation, itself caused by elevation of the O-centered lone pairs in the absence of the K^+^ cation. This enhanced π bonding component is also reflected in the shorter Al–O bond lengths measured for **2** compared to **1** (means: 1.786(2) vs 1.807(3) Å).

In terms of reactivity, **1** is stable in benzene, but **2** reacts with it. Addition of C_6_H_6_ to **2** at room temperature and sonication (for 30 min), followed by filtration and crystallization at room temperature, results in the formation of the [4 + 1] cycloaddition product [K(2.2.2-crypt)][(κ^2^-C_6_H_6_)Al{OB(NDippCH)_2_}_2_], **3**, as yellow crystals. Alternatively, compound **3** can be accessed rapidly by the addition of (2.2.2-crypt) into a benzene solution of **1**, which immediately leads to crystallization of compound **3**. At 25 °C, **3** is insoluble in hydrocarbon solvents (toluene, pentane, and hexane) and undergoes rapid decomposition in halogenated solvents. However, **3** is sparingly soluble in benzene after extended sonication (>1 h), allowing it to be characterized by ^1^H NMR spectroscopy: signals at δ_H_ = 2.51, 3.51, and 3.66 ppm are assigned, respectively, to the protons attached to the sp^3^- and two sp^2^-carbon atoms of the [C_6_H_6_]^2–^ moiety. At 50 °C, **3** exhibits enhanced solubility in C_6_D_6_ but spectral interpretation is hampered by severe broadening of the C_6_H_6_-derived signals (Figure S8).^37−39^

**Figure 3:**
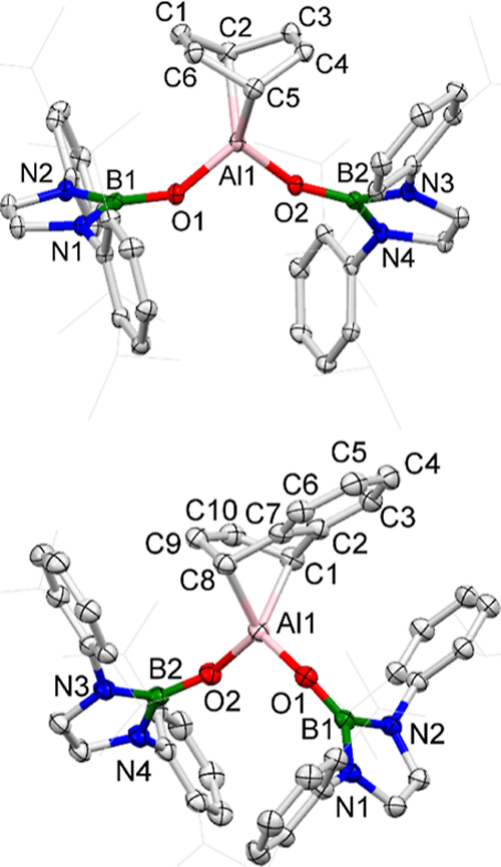
Molecular structures of the anionic components of **3** (upper) and **5** (lower) in the solid state as determined by X-ray crystallography (ellipsoids set at 35% probability level; H atoms, solvent molecules and counterions omitted, and ^*i*^Pr groups shown in the wireframe format for clarity). Key bond lengths (Å) and angles (deg): **3**: B1–O1 1.326(2), B2–O2 1.325(2), Al1–O1 1.745(1), Al1–O2 1.746(1), Al1–C5 2.067(1), Al1–C5 2.052(1), C1–C6, C6–C5 1.499(2), C5–C4 1.498(2), C4–C3 1.337(2), C3–C2 1.504(2), C2–C1 1.501(2); O1–Al1–O2 103.6(5); **4**: B1–O1 1.333(2), B2–O2 1.331(2), Al1–O1 1.737(1), Al1–O2 1.745(1), Al1–C1 2.070(2), Al1–C8 2.057(2), C7–C8, 1.492(2), C7–C2 1.417(2), C8–C9 1.498(3), C2–C1 1.501(2), C1–C10 1.496(2), C9–C10 1.344(3); and O1–Al1–O2 108.7(5).

The molecular structure of **3** in the solid state was confirmed crystallographically (Figure 3), revealing a boat-like [C_6_H_6_]^2–^ fragment. Bond lengths are consistent with a dearomatized (formally doubly reduced) C_6_ fragment, featuring two isolated double bonds [C2–C3, 1.338(2) Å; C5–C6, 1.337(2) Å] and four single bonds [1.498(2)-1.504(2) Å].

**Scheme 2:**
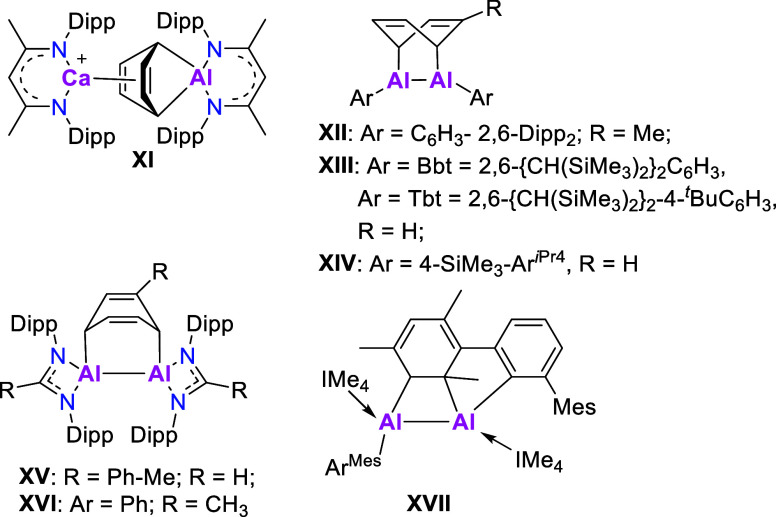
Known Examples of Al^I^-Mediated Cycloaddition of Arenes^37−39,52,59,60^

Subjecting solid samples of **3** to continuous vacuum (ca. 10^–2^ Torr) results in the regeneration of **2** (as judged by multinuclear NMR spectroscopy), signaling that the activation of benzene by this “naked” acyclic aluminyl system is reversible. To our knowledge, the conversion of **2** to **3** represents the first example of reversible single-site [4 + 1] activation of benzene at a metal center. The regeneration of **2** from **3** involves formal reductive elimination via the Al^III^/Al^I^ redox couple,^16,38−42^ a phenomenon which is very rare for spontaneous processes in aluminum chemistry.^9^ The cycloaddition of benzene by systems incorporating aluminum has been documented for compounds **XI** and **XIII–XV** (Scheme 2), with the heterobimetallic system **XI** undergoing exchange of the [C_6_H_6_]^2–^ fragment with C_6_D_6_.^37^ Additionally, reversible Büchner-type ring expansion of arenes via low-valent Al^I^ and Si^II^,^43−45^ and main group compound-mediated [4 + 1],^46,47^ [4 + 2],^48−50^ [4 + 3],^51^ [2 + 2],^52^ and [2 + 1]^53−55^ cycloaddition of arenes have also been reported.^56−58^

**Scheme 3:**
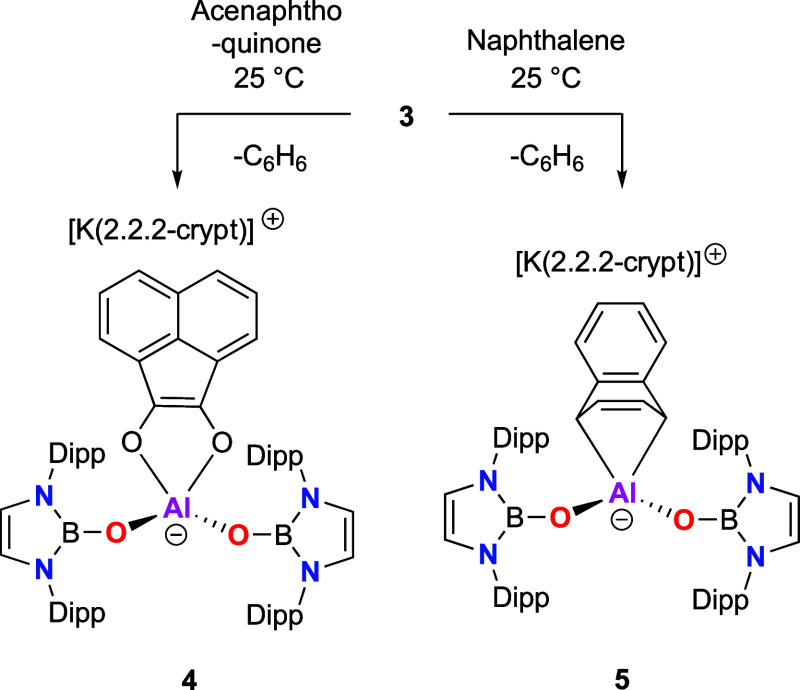
Synthesis of Compounds **4–5** via Benzene Displacement from **3**

To provide chemical corroboration of reversible benzene addition to **2**, we examined the reactivity of **3** toward other arenes (naphthalene and anthracene) and toward Al^I^ trapping agents such as quinones. Accordingly, the reactions of **3** with acenaphthoquinone and naphthalene result in the formation of new Al^III^ compounds **4** and **5**, via substitution of the [C_6_H_6_]^2–^ fragment (Scheme 3). The identities of **4** and **5** were confirmed by NMR and X-ray diffraction studies (Figures 3 and S25). Both compounds can also be synthesized through the direct reactions of “naked” aluminyl complex **2** with acenaphthoquinone/naphthalene, thereby confirming that compound **3** acts as a masked Al^I^ center via reversible benzene uptake.

In a broader context, the [4 + 1] cycloaddition reactions of **2** toward benzene and naphthalene contrast with the chemistry of its NON-supported analogue [K(2.2.2-crypt)][(NON)Al]. [K(2.2.2-crypt)][(NON)Al] undergoes C–C and C–H oxidative addition reactions with benzene and naphthalene, respectively.^16^ Interestingly, **1** does not appear to react with naphthalene. With this in mind, and in order to better understand the reactivity of NHBO-supported aluminyl compounds toward arenes, we examined via quantum chemical methods different pathways for the reactions of **1** and [Al{OB(NDippCH)_2_}_2_]^−^ (the anionic component of **2**) toward benzene, i.e., oxidative addition (C–C/C–H) and [4 + 1] cycloaddition.

**Figure 4:**
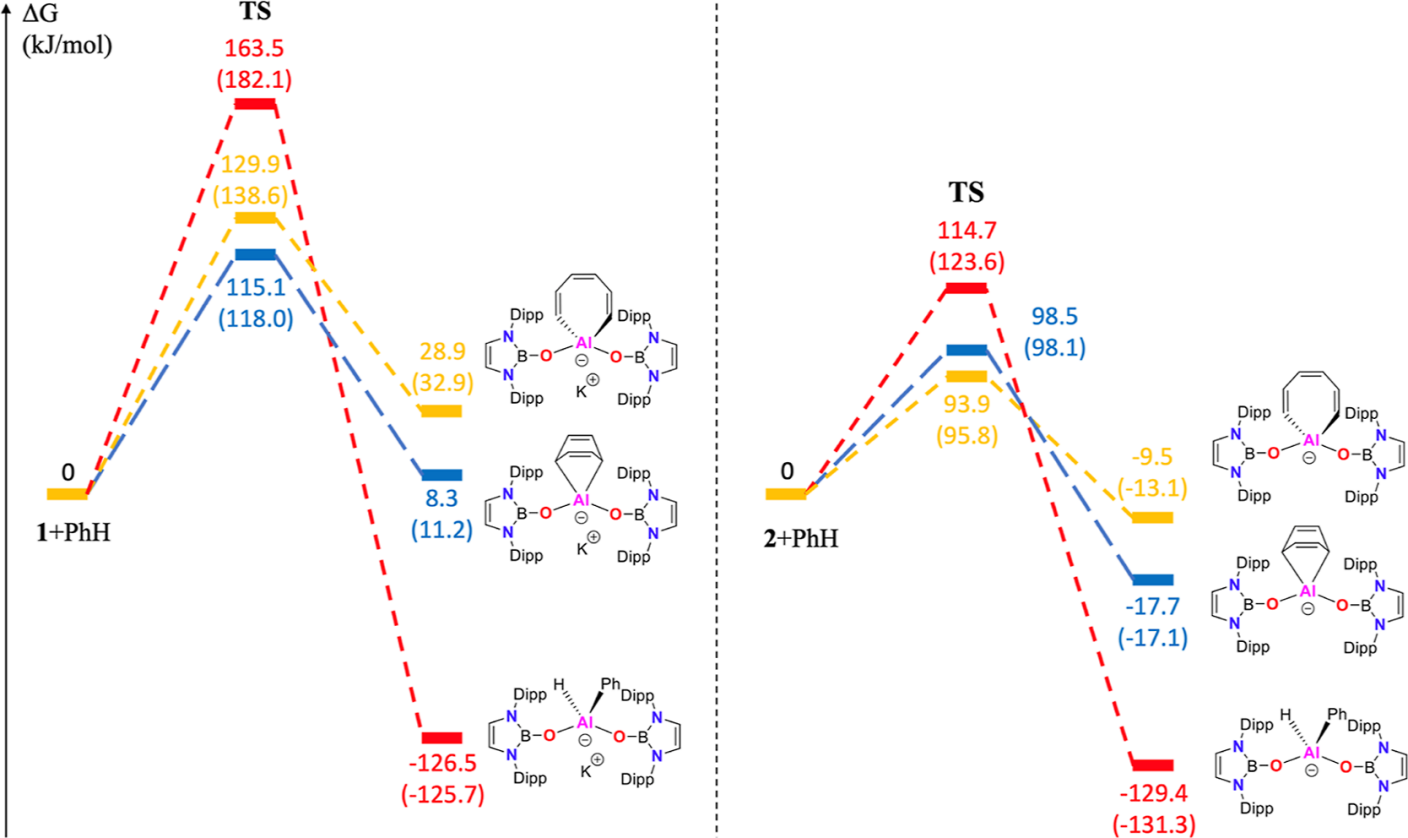
Free-energy plots (kJ mol^**–1**^) for the activation of benzene by **1** (left) and **2** (right) in either THF or benzene. Red: C–H insertion; yellow: C–C insertion; and blue: [4 + 1] cycloaddition [calculated at the PBE1PBE-GD3BJ/Def2-TZVP(SMD, solvent = THF/benzene)//PBE1PBE-GD3BJ/Def2-SVP level; figures in parentheses refer to benzene as the solvent continuum].

In the case of **1**, both cycloaddition and C–C insertion reactions are thermodynamically unfavorable (by 8.3 and 28.9 kJ mol^–1^, respectively, in THF; Figure 4). C–H activation to give an Al^III^ phenyl/hydride is highly exergonic (−126.5 kJ mol^–1^) but involves a prohibitive activation barrier (163.5 kJ mol^–1^). The lack of reactivity of **1** toward benzene and naphthalene is therefore rationalized on either thermodynamic (cycloaddition, C–C insertion) or kinetic grounds (C–H insertion). The “naked” aluminyl complex, by contrast, is calculated to undergo C–C insertion and [4 + 1] cycloaddition reactions via comparable activation barriers (93.9 and 98.5 kJ mol^–1^ in THF), to yield products that are thermodynamically more stable than [Al{OB(NDippCH)_2_}_2_]^−^ plus benzene (by −9.5 and −17.7 kJ mol^–1^, respectively, Figure 4). The isolation of **3** as the sole product suggests that C–C activation—while kinetically more facile—is reversible, and that the more exergonic (but slightly less facile) [4 + 1] cycloaddition pathway can therefore be accessed at room temperature. The activation barrier associated with the (ultimately much more exergonic) C–H activation reaction is significantly higher at +114.7 kJ mol^–1^. Consistently, **3** decomposes at temperatures above 90 °C in benzene, albeit to give a mixture of products, suggesting multiple C–H activation pathways.

**Scheme 4:**
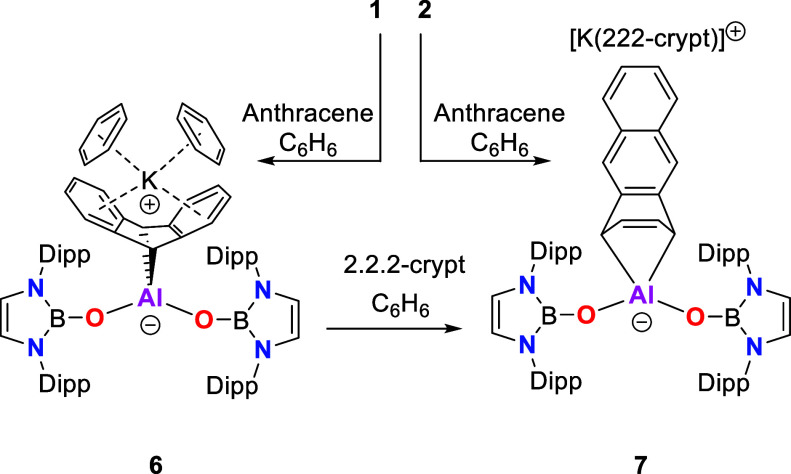
Syntheses of Compounds **6–7** via [4 + 1] Cycloaddition of Anthracene

**Figure 5:**
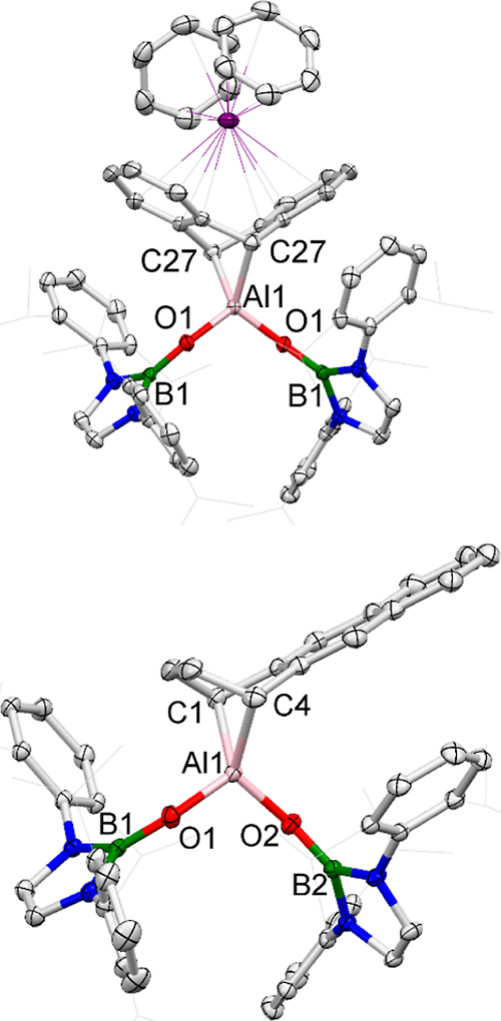
Molecular structures of **6** (upper) and of the anionic component of compound **7** (lower) in the solid state, as determined by X-ray crystallography (ellipsoids set at 35% probability level; H atoms, solvent molecules and counterions (for **7**) omitted, and ^*i*^Pr groups shown in the wireframe format for clarity). Key bond lengths (Å) and angles (deg): compound **6**: Al1–O1 1.729(17), Al1–C27 2.068(3); Al1–O1 110.7(12); compound **7**: Al1–O1 1.740(1), Al1–O2 1.733(9), B1–O1 1.322(2), B2–O2 1.328(1), Al1–C1 2.069(1), Al1–C1 2.056(2); O1–Al1–O2 108.5(5).

We also explored the reactivity of compounds **1** and **2** toward anthracene: both compounds undergo a [4 + 1] cycloaddition reaction, albeit with different regiochemistries. **1** selectively adds across the 9,10-positions, yielding **6**; more surprisingly, **2** adds across the 1,4 positions, leading to the formation of unsymmetrically derivatized anthracenediyl compound **7** (Scheme 4 and Figure 5).

Cycloaddition across the 9,10-positions of anthracene is typically more favorable thermodynamically, owing to the greater aromatic stabilization associated with two isolated benzene π systems over a single naphthalene unit.^61−63^ With this in mind, we sought to explore the possibility for the challenging conversion of **6** to its less common regio-isomer **7** by sequestration of the K^+^ counterion. In this event, addition of 2.2.2-cryptand to a benzene solution of **6** results in immediate formation of **7**; the addition of THF-*d*_8_ to **6** also leads to the rapid formation of the 1,4-activated product, presumably due to K^+^ sequestration by THF.

K^+^ “chelation” by the two π systems formed by the 9,10-activation of anthracene (Figure 5) offers an additional stabilizing interaction in **6** which is not available in the 1,4 product. As such, 9,10-activation across the central ring is favored in the presence of K^+^. On the other hand, the steric demands of the NHBO ligands offer a possible rationale for the alternative 1,4-regiochemistry in the absence of K^+^. The closest contacts between the anthracenediyl and NHBO fragments in **6** occur between the ^*i*^Pr groups of the boryloxy ligands and the 2-/3-positions of the carbocycle on both sides of the complex [closest C···C contacts: 3.356(6) Å]. By contrast, the more unsymmetrical nature of the 1,4-activated anthracene unit means that its steric profile is much diminished in the region of one of the NHBO ligands while maintaining a similar separation with the other boryloxy donor to that observed in **6** [closest C···C contact: 3.509(2) Å]. As such, we postulate that steric factors underpin the regioselectivity observed in the reaction of **2** with anthracene. Issues of regioselectivity were also explored by DFT calculations: 1,4- and 9,10-activated anthracene isomers were optimized both for zwitterionic **6** (without benzene molecules) and for the anionic component of **7**. In the case of **6**, the Gibbs free-energy difference was calculated at +24.7 kJ mol^–1^ in favor of the 9,10-product, in agreement with the observed stabilizing effects of the K^+^ cation. Removing the potassium cation diminishes the energy difference between the two isomers to close to zero (+5.7 kJ mol^–1^), with the implication that specific solvation or crystal packing effects (in solution/the solid state) are likely to be regioselectively important. Consistent with the role of steric factors in determining the regiochemistry of the reaction of **7** with anthracene, truncation of the NHBO ligands in silico (by replacement of the Dipp ^*i*^Pr groups by H) biases the thermodynamics in favor of the 9,10-isomer by 49.7 kJ mol^–1^.

## Conclusions

In conclusion, we have introduced the first example of a “naked” acyclic aluminyl compound, [AlX_2_]^−^, by exploiting the strongly stabilizing properties of the NHBO ligand on the aluminum-centered lone pair. In the absence of K^+^ (sequestered by 2.2.2-crypt), this system shows unique capabilities: in the reversible single-site activation of benzene via a [4 + 1] cycloaddition process and in the functionalization of anthracene via 1,4-cycloaddition at the noncentral carbocycle. The critical role played by K^+^ sequestration in this chemistry relates not only to the role of the cation in further stabilizing the aluminum-centered lone pair in the aluminyl reactant (by > 1.1 eV) but also in removing the possibility for (favorable) K^+^/arene contacts in one of the products.

## Experimental Section

### [KAl{OB(NDippCH)_2_}_2_] [**1**]

To a mixture of K_[OB(NDippCH)2]_ (500 mg, 1.13 mmol) and [Cp*Al]_4_ (92 mg, 0.14 mmol), benzene (3 mL) was added and stirred for 4 h at 80 °C to form a light yellow-green solution. The solution was filtered, and all volatiles were removed under reduced pressure, yielding compound **1** as a pale-yellow powder. The yield was 0.46 g (0.53 mmol, 93%). For crystallization, 50 mg of compound **1** was placed in a J. Young NMR tube, and 0.5 mL of hexane was added. The mixture was then heated at 80 °C overnight, during which yellow crystals formed at the neck of the NMR tube. Subsequently, the NMR tube was transferred to a glovebox, and the crystals were carefully collected. These crystals are suitable for single-crystal X-ray diffraction analysis. ^1^H NMR (400 MHz, C_6_D_6_, 297 K): δ = 1.17 (d, ^3^*J*_H–H_ = 7 Hz, 24H, CH(C*H*_3_)_2_), 1.24 (d, ^3^*J*_H–H_ = 7 Hz, 24H, CH(C*H*_3_)_2_), 3.38 (sept, ^3^*J*_H–H_ = 7 Hz, 8H, C*H*(CH_3_)_2_), 5.94 (s, 4H, NC*H*), 6.98–7.05 (Ar^Dipp^-*H*, 12H); ^13^C{^1^H} NMR (100 MHz, C_6_D_6_): δ = 24.0 (CH(*C*H_3_)_2_), 24.5 (CH(*C*H_3_)_2_), 28.5 (*C*H(CH_3_)_2_), 115.9 (N*C*H), 123.5 (Dipp-m-CH), 127.0 (Dipp-p-CH), 140.7 (Dipp-i-C), 147.8 (Dipp-o-C); ^11^B{^1^H} NMR (128 MHz, C_6_D_6_): δ = 22.7; Anal. Calcd. [%] for C_52_H_72_AlB_2_KN_4_O_2_: C, 71.55; H, 8.31; N, 6.42. Found: C, 71.27; H, 8.14; N, 6.17.

### [K(2.2.2-cryptand)][(HCDippN)_2_BO]_2_Al] [**2**]

A 2 mL of THF solution of **1** (100 mg, 0.1 mmol) was gradually added to [2.2.2]-cryptand (43.1 mg, 0.1 mmol) in 3 mL of THF. The solution was stirred for 15 min at room temperature and then evaporated, resulting in an orange-yellow powder of compound **2** (132 mg, 0.1 mmol, 92% yield). For crystallization: A 1 mL of mesitylene solution of **1** (100 mg, 0.1 mmol) was slowly added to [2.2.2]-cryptand (43.1 mg, 0.1 mmol) in mesitylene (3 mL) and kept for 2 days at room temperature without stirring. After 2 days, yellow crystals of compound **2** were obtained, which were suitable for X-ray diffraction analysis. ^1^H NMR (400 MHz, THF-*d*_8_, 297 K): δ = 0.97 (d, ^3^*J*_H–H_ = 7 Hz, 24H, CH(C*H*_3_)_2_), 1.03 (d, ^3^*J*_H–H_ = 7 Hz, 24H, CH(C*H*_3_)_2_), 2.55 (m,12H,NC*H*_2_-crypt.), 3.30 (sept, ^3^*J*_H–H_ = 7 Hz, 8H, C*H*(CH_3_)_2_), 3.53–3.56 [br, 24H, {(12H, NCH_2_C*H*_2_crypt)+ (12H, OC*H*_2_-crypt)}], 5.58 (s, 4H, NC*H*), 6.98–7.05 (Ar^Dipp^-*H*, 12H); ^13^C{^1^H} NMR (100 MHz, THF-*d*_8_): δ = 24.9 (CH(*C*H_3_)_2_), 25 (CH(*C*H_3_)_2_), 28.8 (*C*H(CH_3_)_2_), 54.9 (N*C*H_2_-crypt.), 68.6 (NCH_2_*C*H_2_-crypt.), 71.5 (O*C*H_2_-crypt.), 116.0 (N*C*H), 123.0, 125.6 (Dipp-m-*C*H), 129.2 (Dipp-p-*C*H), 142.8 (Dipp-i-*C*), 147.8 (Dipp-o-*C*); ^11^B{^1^H} NMR (128 MHz, THF-*d*_8_): δ = 19.8. Anal. Calcd. [%] for C_70_H_108_AlB_2_KN_6_O_8_: C, 67.30; H, 8.71; N, 6.73. Found: C, 67.05; H, 8.26; N, 6.59.

### [K(2.2.2-cryptand)][(HCDippN)_2_BO]_2_Al(**C**_**6**_**H**_**6**_)] [**3**]

A 2 mL of C_6_H_6_ solution of **1** (100 mg, 0.1 mmol) was gradually added to [2.2.2]-cryptand (43.1 mg, 0.1 mmol) in 3 mL of C_6_H_6_. The solution was kept for 2 days without stirring, resulting in the formation of light-yellow crystals of compound **3**. The crystals were washed with hexane and allowed to dry under argon inside the glovebox, with the sample vial kept open for 5 days (127 mg, 0.09 mmol, 84% yield). Alternatively, 25 mg of compound **2** was added to benzene, sonicated for 30 min, filtered, and left at room temperature without stirring. This process resulted in the formation of crystals of compound **3** after 2 days. NB: under a high vacuum, **3** releases benzene and forms **2**. Compound **3** is insoluble in common organic solvents such as C_6_H_6_, toluene, hexane, etc., at 25 °C. However, upon sonication of **3** in C_6_D_6_, it shows partial solubility, suitable for ^1^H NMR experiments. However, at 50 °C, **3** shows high solubility in C_6_D_6_, but a decrease in the (Al–C*H*) signal is observed in the ^1^H spectra, suggesting an exchange between C_6_H_6_ and C_6_D_6_ (Figure S8). Intriguingly, addition of THF-*d*_8_ to **3** leads to the decoordination of the C_6_H_6_ moiety (Figure S9). ^1^H NMR (400 MHz, C_6_D_6_, 297 K): δ = 1.42–1.47 (br, 48H, CH(C*H*_3_)_2_)), 1.99 (m,12H, NC*H*_2_-crypt.), 2.54 (m, 2H, Al–C*H*), 2.97–3.04 [br, 24H, {(12H, NCH_2_C*H*_2_crypt)+ (12H, OC*H*_2_- crypt)}], 3.51–3.66 (br, 4H, C^2,3,5,6^-*H*, C_6_H_6_), 3.79–3.88 (br, 8H, C*H*(CH_3_)_2_), 6.05–6.11 (s, 4H, NC*H*), 7.29–7.26 (Ar^Dipp^-*H*, 12H). Due to the poor solubility of **3**, a suitable ^13^C NMR spectrum of compound **3** was not observed.

### [[(HCDippN)_2_BO]_2_AlK(anthracene)] [**6**]

A benzene solution (2 mL) of **1** (0.1 g, 0.01 mmol) was added to anthracene (20.4 mg, 0.01 mmol) in 2 mL of benzene at room temperature. The color of the solution rapidly changed from orange to light yellow. After stirring the solution for 4 h, it was filtered via cannula filtration. The solution was then concentrated to 1 mL, and hexane (1 mL) was added to aid in crystallization. After 7 days, colorless crystals of compound **6** were obtained, which were suitable for X-ray diffraction analysis. For further analysis, the solvent was decanted, and the colorless crystals were washed once with hexane and dried in a high vacuum. Compound **6** was isolated as a colorless crystalline material (91 mg, 0.08 mmol, 75% yield). NB: after crystallization, **6** is poorly soluble in common aromatic and aliphatic organic solvents. The addition of THF-*d*_8_ to compound **6** or to a 1:1 mixture of **1** and anthracene leads to the rapid formation of the 1,4-activated product, presumably due to K+ sequestration by THF-*d*_8_ (Figure S19). ^1^H NMR (400 MHz, Toluene-*d*_8_, 297 K): δ = 1.08–1.19 (m, 48H, CH(C*H*_3_)_2_), 2.98 (br 2H, C^9,10^-*H*, anthra), 3.27 (br, 8H, C*H*(CH_3_)_2_), 5.76 (s, 4H, NC*H*), 6.28–6.37 (br, 8H, C^1,2,3,4,5,6,7,8^-*H*, anthra), 6.96–7.07 (br, 12H, Ar^Dipp^-*H*). ^**13**^C{^1^H} NMR (100 MHz, C_6_D_6_): δ = 23.6–25.5 (CH(*C*H_3_)_2_), 26.2–28.5 (*C*H(CH_3_)_2_), 115.6–117.1 (N*C*H), 122.6–123.5 (Dipp-m-*C*H), 125.5–126.7 (*C*H, C^1,4,5,8^-Anth), 127.5–128.0 (*C*H, C^2,3,6,7^-Anth), 131.3–132.2 (Dipp-p-*C*H), 140.0 (Dipp-i-C), 141.1 (*C*, C^4a,8a,9a,10a^-Anth), 147.8 (Dipp-o-C). ^**11**^B{^1^H} NMR (128 MHz, C_6_D_6_): δ = 20.2. Anal. Calcd. [%] for C_66_H_82_AlB_2_KN_4_O_2_: C, 75.42; H, 7.86; N, 5.33. Found: C, 74.98; H, 7.36; N, 5.13.

### [K(2.2.2-cryptand)][[(HCDippN)_2_BO]_2_Al(anthracene)] [**7**]

A THF (3 mL) solution of **2** (0.1 g, 0.08 mmol, 1.00 equiv) was added to anthracene (14.2 mg, 0.08 mmol, 1.00 equiv) in 2 mL of THF at room temperature. The color of the solution rapidly changed from orange to light yellow. After stirring the solution for 1 h, it was filtered via cannula filtration. The solution was concentrated to 1 mL, and hexane (1 mL) was added to aid crystallization. After 8 days, colorless crystals of compound **7** were obtained, which were suitable for X-ray diffraction analysis. For further analysis, the solvent was decanted, and the colorless crystals were washed once with hexane and dried under high vacuum. Compound **7** was isolated as a colorless crystalline material (110 mg, 0.08 mmol, 75% yield). Alternatively compound **7** could be isolated via the treatment of THF solution of **6** (1 equiv) with 1 eq of [2.2.2-cryptand] or reaction of **3** (1 equiv) with anthracene (1 equiv). ^1^H NMR (400 MHz, THF-*d*_8_, 297 K): δ = 0.52–1.09 (m, 48H, CH(C*H*_3_)_2_), 2.36 (m,12H, NC*H*_2_-crypt.), 2.45 (m, 2H, C^1,4^-*H*, anthra), 2.94 (br, 4H, C*H*(CH_3_)_2_), 3.31–3.39 [br, (24H, OC*H*_2_crypt, NCH_2_C*H*_2_crypt) + (4H, C*H*(CH_3_)_2_)], 4.85 (m, 2H, C^2,3^-*H*, anthra), 5.44–5.60 (s, 4H, NC*H*), 6.31 (m, 2H, C^9,10^-*H*, anthra), 6.78 (m, 2H, C^5,8^-*H*, anthra), 7.06–7.14 (br, 12H, Ar^Dipp^-*H*), 7.18 (m, 2H, C^6,7^-*H*, anthra); ^13^C{^1^H} NMR (100 MHz, THF-*d*_8_): δ = 23.8–23.9 (CH(*C*H_3_)_2_), 28.4–28.7 (*C*H(CH_3_)_2_), 54.7 (NCH_2_–crypt.), 68.4 (NCH_2_*C*H_2_-crypt.), 71.2 (O*C*H_2_-crypt.), 114.9 (C^9,10^-anthra), 116.6–117.0 (N*C*H), 120.1 (C^5,8^-anthra), 123.2 (C^2,3^-anthra), 125.9–126.2 (Dipp-m-CH), 126.6 (C^6,7^-anthra), 132.4 (C^4a,9a^-anthra), 141.7–142.5 (Dipp-i-C), 148.0 (Dipp-o-C), 150.9 (C^8a,10a^-anthra). ^11^B{^1^H} NMR (128 MHz, THF-*d*_8_): δ = 21.4. Anal. Calcd. [%] for C_84_H_118_AlB_2_KN_6_O_8_: C, 70.67; H, 8.33; N, 5.89. Found: C, 70.14; H, 8.09; N, 5.55.

## Supporting Information Available

Complete synthetic and characterizing data for new compounds; representative spectra; X-ray crystal structures; and details of quantum chemical calculations (PDF)

## Abbreviations

Dipp = 2: 6-diisopropylphenyl
NON = 4: 5-bis(2,6-diisopropylanilido)-2,7-di-*tert*-butyl-9,9-dimethylxanthene)
Cp* = 1: 2,3,4,5-pentamethylcyclopentadienyl
^Mes^Ar = 2: 6-(2,4,6-Me_3_C_6_H_2_)_2_C_6_H_3_
IMe_4_ = 1: 3,4,5-tetramethylimidazol-2-ylidene
