# Supplementary material for: Reversible [4 + 1] Cycloaddition of Arenes by a “Naked” Acyclic Aluminyl Compound
Source: J Am Chem Soc. 2024 Apr 16;146(17):11792–800. doi: 10.1021/jacs.4c00376 (PMC11066863; doi:10.1021/jacs.4c00376)
Supplement: Supplementary file 1 — ja4c00376_si_001.pdf [file ja4c00376_si_001.pdf]

Reversible [4+1] cycloaddition of Arenes by a 'Naked' Acyclic Aluminyl Compound

Authors: Debotra Sarkar,*,† Petra Vasko,‡ Aisling F. Roper,† Agamemnon E. Crumpton,† Matthew M.D. Roy,† Liam P. Griffin,† Charlotte Bogle,† and Simon Aldridge*,†

Affiliation:

† Research Laboratory, Department of Chemistry, University of Oxford, Oxford, OX1 3TA, United Kingdom

‡ Department of Chemistry, University of Helsinki, A.I. Virtasen aukio 1, P.O. Box 55, FI-00014, Finland

Table of Contents

Experimental Procedures 1

Preparation of [KAl{OB(NDippCH)$_2$}$_2$] [1] 1

Preparation of [K(2.2.2-cryptand)][(HCDippN)$_2$BO]$_2$Al] [2] 4

Preparation of [K(2.2.2-cryptand)][(HCDippN)$_2$BO]$_2$Al(C_6H_6)] [3] 7

Preparation of [K(2.2.2-cryptand)][(HCDippN)$_2$BO]$_2$Al ($C_{12}H_6O_2$)] [4] 10

Preparation of [K(2.2.2-cryptand)][[(HCDippN)$_2$BO]$_2$Al(naphthalene)] [5] 13

Preparation of [[(HCDippN)$_2$BO]$_2$AlK(anthracene)] [6] 16

Preparation of [K(2.2.2-cryptand)][[(HCDippN)$_2$BO]$_2$Al(anthracene)] [7] 20

Details for DFT Calculations 23

Single crystal X-Ray structure determination: 58

References: 65

Experimental Procedures

All manipulations were carried out using standard Schlenk line or dry-box techniques under an atmosphere of argon or dinitrogen. Solvents were degassed by sparging with argon and dried by passing through a column of the appropriate drying agent. Xylenes were refluxed over potassium, with the solvent then being distilled and stored under argon in Teflon valve ampoules. NMR spectra were measured in C_6D_6 (which was dried over potassium) or THF-d_8 (which was dried over $LiAlH_4$), with the solvent then being distilled under reduced pressure and stored under argon in Teflon valve ampoules. ^{1}H, ^{13}C{^{1}H} and ^{11}B{^{1}H} NMR spectra were recorded on Bruker 400 MHz spectrometer at ambient temperature and referenced internally to residual protio-solvent (^{1}H) or solvent (^{13}C) resonances and are reported relative to tetramethylsilane (δ = 0 ppm). Chemical shifts are quoted in δ (ppm) and coupling constants in Hz. Elemental analyses were carried out by London Metropolitan University. (HCDippN)$_2$BOK[1] and [Cp*Al]$_4$ was prepared by the literature method.[2]

Preparation of [KAl{OB(NDippCH)$_2$}$_2$] [1]

To a mixture of K[OB(NDippCH)$_2$] (500 mg, 1.13 mmol) and [Cp*Al]$_4$ (92 mg, 0.14 mmol), benzene (3 mL) was added and stirred for four hours at 80 °C to form a light yellow-green solution. The solution was filtered, and all

volatiles were removed under reduced pressure, yielding compound **1** as a pale-yellow powder. The yield was 0.46 g (0.53 mmol, 93%).

For crystallization, 50 mg of compound **1** was placed in a J. Young NMR tube, and 0.5 ml of hexane was added. The mixture was then heated at 80°C overnight, during which yellow crystals formed at the neck of the NMR tube. Subsequently, the NMR tube was transferred to a glove box, and the crystals were carefully collected. These crystals are suitable for single-crystal X-ray diffraction analysis.

^{1}H NMR (400 MHz, C_6D_6, 297 K): δ = 1.17 (d, $^3J_{H\text{-}H}$ = 7 Hz, 24H, CH(C*H*$_3$)$_2$), 1.24 (d, $^3J_{H\text{-}H}$ = 7 Hz, 24H, CH(C*H*$_3$)$_2$), 3.38 (sept, $^3J_{H\text{-}H}$ = 7 Hz, 8H, C*H*(CH$_3$)$_2$), 5.94 (s, 4H, NC*H*), 6.98-7.05 (Ar^{Dipp}-*H*, 12H); **^{13}C{^{1}H} NMR** (100 MHz, C_6D_6): δ = 24.0 (CH(*C*H$_3$)$_2$), 24.5 (CH(*C*H$_3$)$_2$), 28.5 (*C*H(CH$_3$)$_2$), 115.9 (N*C*H), 123.5 (Dipp-m-CH), 127.0 (Dipp-p-CH), 140.7 (Dipp-i-C), 147.8 (Dipp-o-C); **^{11}B{^{1}H} NMR** (128 MHz, C_6D_6): δ = 22.7; Anal. Calcd. [%] for $C_{52}H_{72}AlB_2KN_4O_2$: C, 71.55; H, 8.31; N, 6.42. Found: C, 71.27; H, 8.14; N, 6.17.

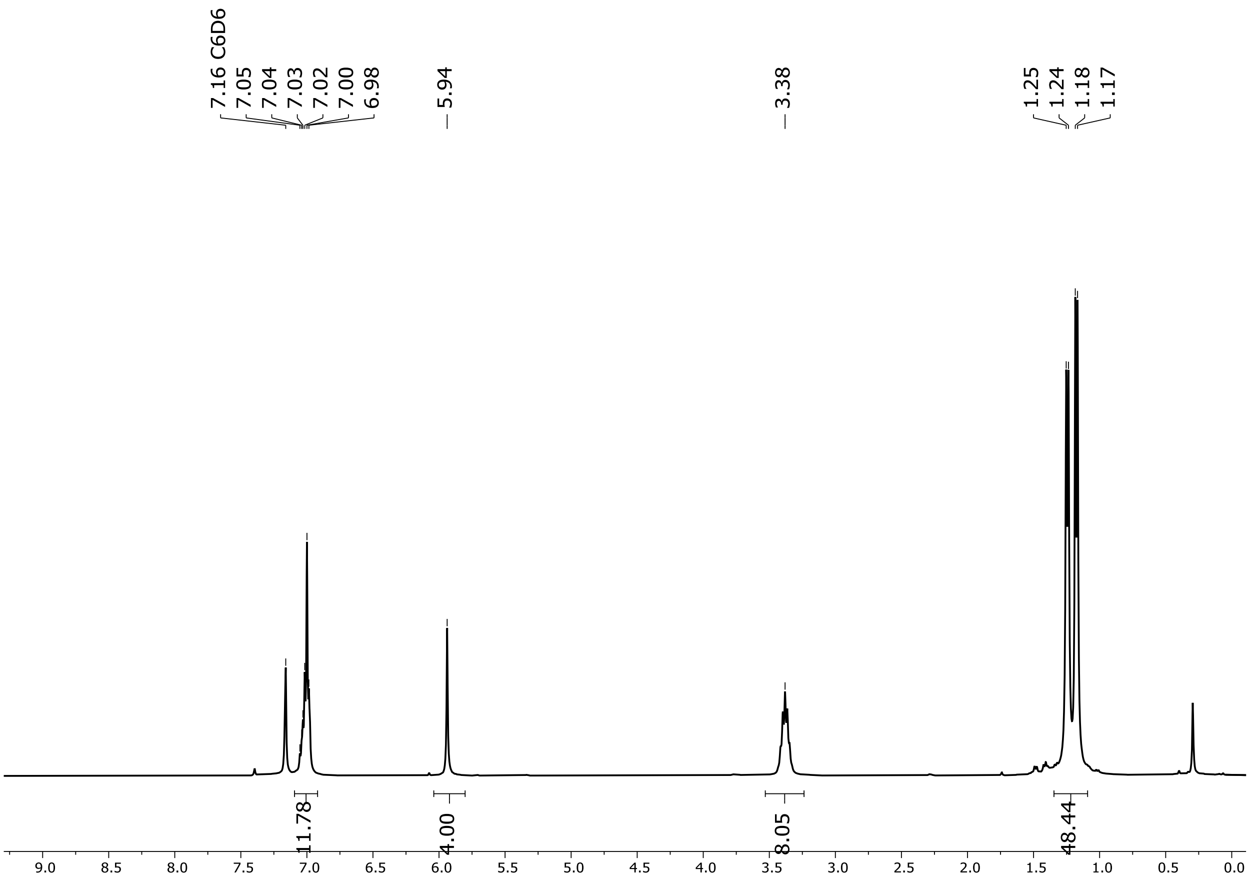

Figure S1. ^{1}H NMR spectrum of compound **1**.

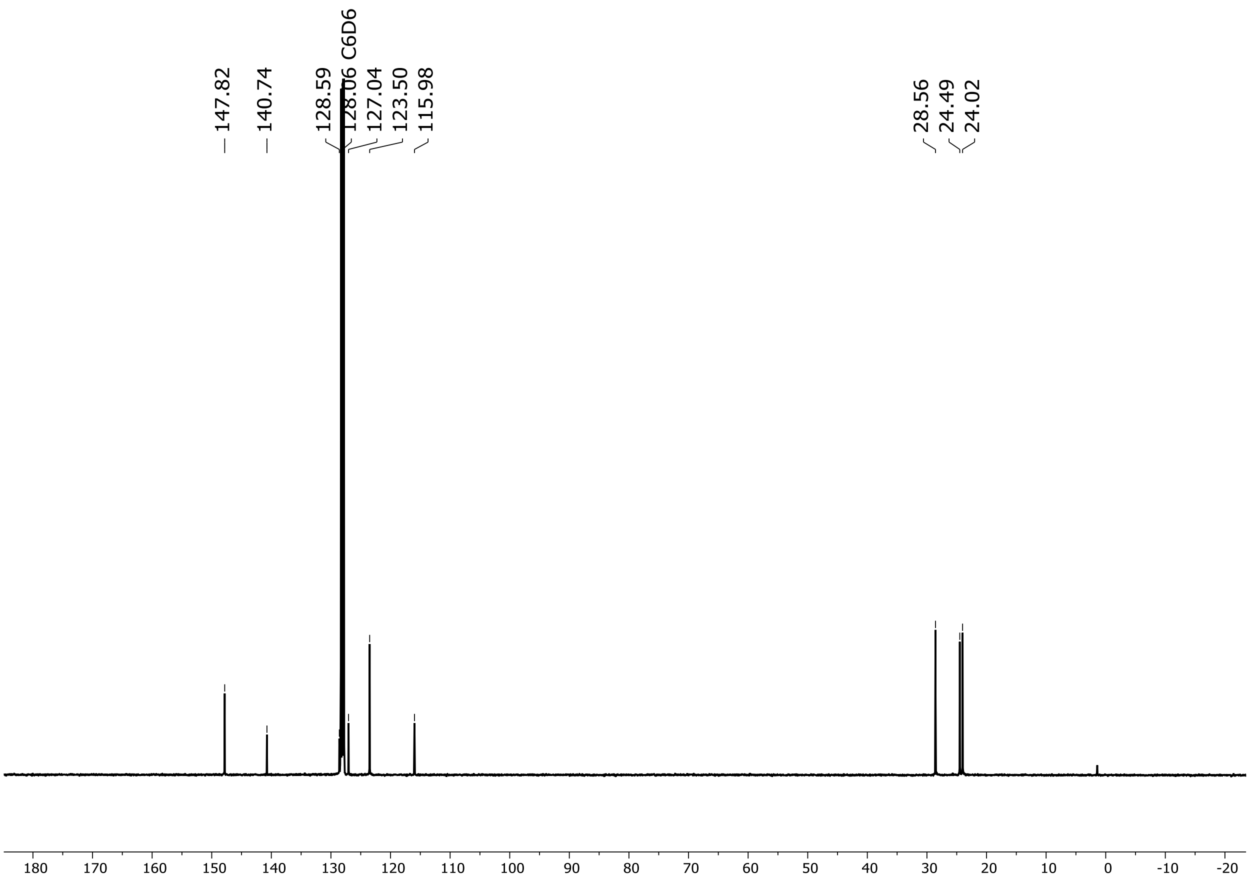

Figure S2. $^{13}C\{^{1}H\}$ NMR spectrum of compound **1**.

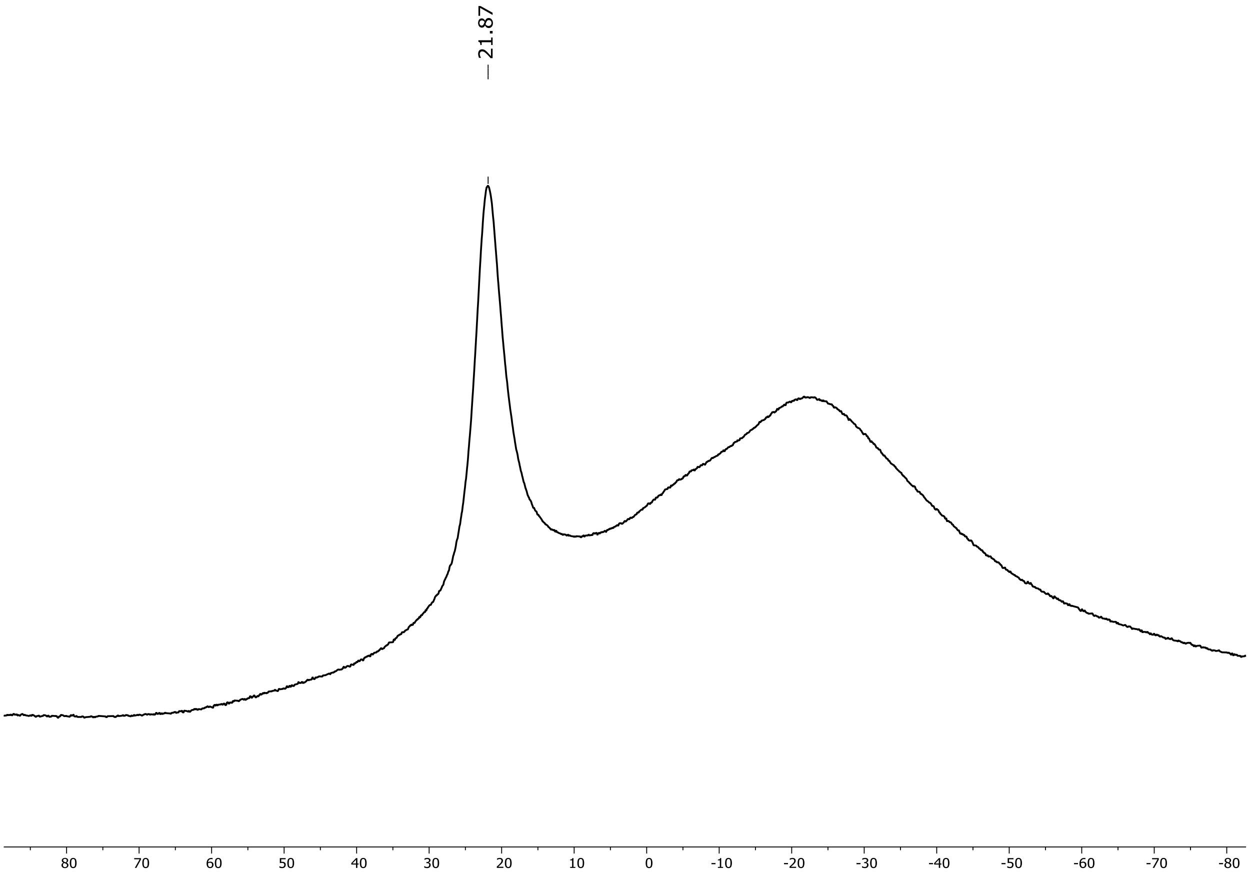

Figure S3. $^{11}B\{^{1}H\}$ NMR spectrum of compound **1**.

Preparation of [K(2.2.2-cryptand)][(HCDippN)$_2$BO]$_2$Al] [2].

A 2 mL THF solution of **1** (100 mg, 0.1 mmol) was gradually added to [2.2.2]-cryptand (43.1 mg, 0.1 mmol) in 3 mL of THF. The solution was stirred for 15 minutes at room temperature and then evaporated, resulting in an orange-yellow powder of compound **2** (132 mg, 0.1 mmol, 92 % yield).

For crystallization: A 1 mL mesitylene solution of **1** (100 mg, 0.1 mmol) was slowly added to [2.2.2]-cryptand (43.1 mg, 0.1 mmol) in mesitylene (3 mL) and kept for two days at room temperature without stirring. After two days, yellow crystals of compound **2** were obtained, which were suitable for X-ray diffraction analysis.

^{1}H NMR (400 MHz, THF-d$_8$, 297 K): δ = 0.97 (d, $^{3}J_{H\text{-}H}$ = 7 Hz, 24H, CH(C*H*$_3$)$_2$), 1.03 (d, $^{3}J_{H\text{-}H}$ = 7 Hz, 24H, CH(C*H*$_3$)$_2$), 2.55 (m,12H,NC*H*$_2$-crypt.), 3.30 (sept, $^{3}J_{H\text{-}H}$ = 7 Hz, 8H, C*H*(CH$_3$)$_2$), 3.53- 3.56 [br, 24H, {(12H, NCH$_2$C*H*$_2$crypt)+ (12 H, OC*H*$_2$- crypt)}], 5.58 (s, 4H, NC*H*), 6.98-7.05 (Ar^{Dipp}-*H*, 12H); **^{13}C{^{1}H} NMR** (100 MHz, THF-d$_8$): δ = 24.9 (CH(*C*H$_3$)$_2$), 25 (CH(*C*H$_3$)$_2$), 28.8 (*C*H(CH$_3$)$_2$), 54.9 (N*C*H$_2$-crypt.), 68.6 (NCH$_2$*C*H$_2$-crypt.), 71.5 (O*C*H$_2$-crypt.), 116.0 (N*C*H), 123.0, 125.6 (Dipp-m-*C*H), 129.2 (Dipp-p-*C*H), 142.8 (Dipp-i-*C*), 147.8 (Dipp-o-*C*); **^{11}B{^{1}H} NMR** (128 MHz, THF-d$_8$): δ = 19.8. Anal. Calcd. [%] for $C_{70}H_{108}AlB_2KN_6O_8$: C, 67.30; H, 8.71; N, 6.73. Found: C, 67.05; H, 8.26; N, 6.59.

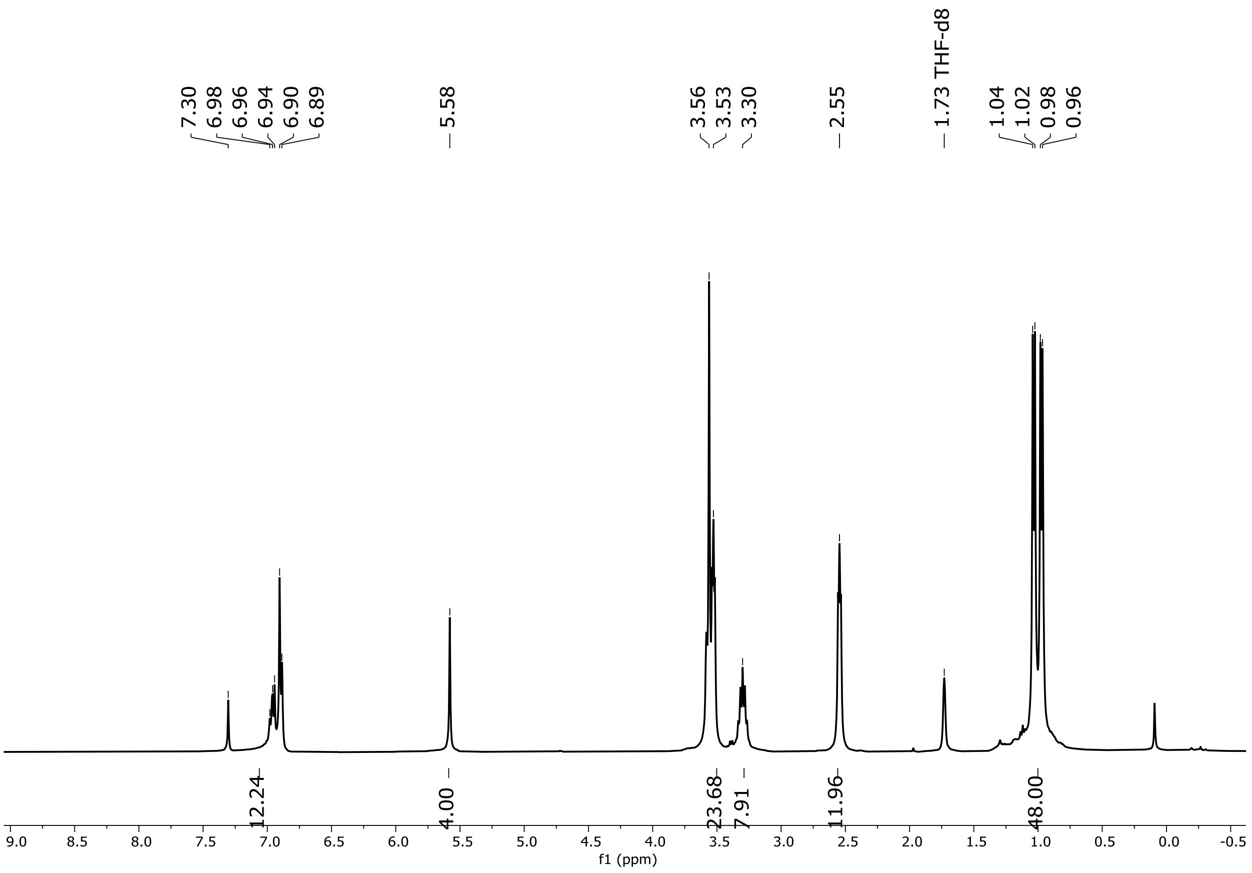

Figure S4. ^{1}H NMR spectrum of compound **2**.

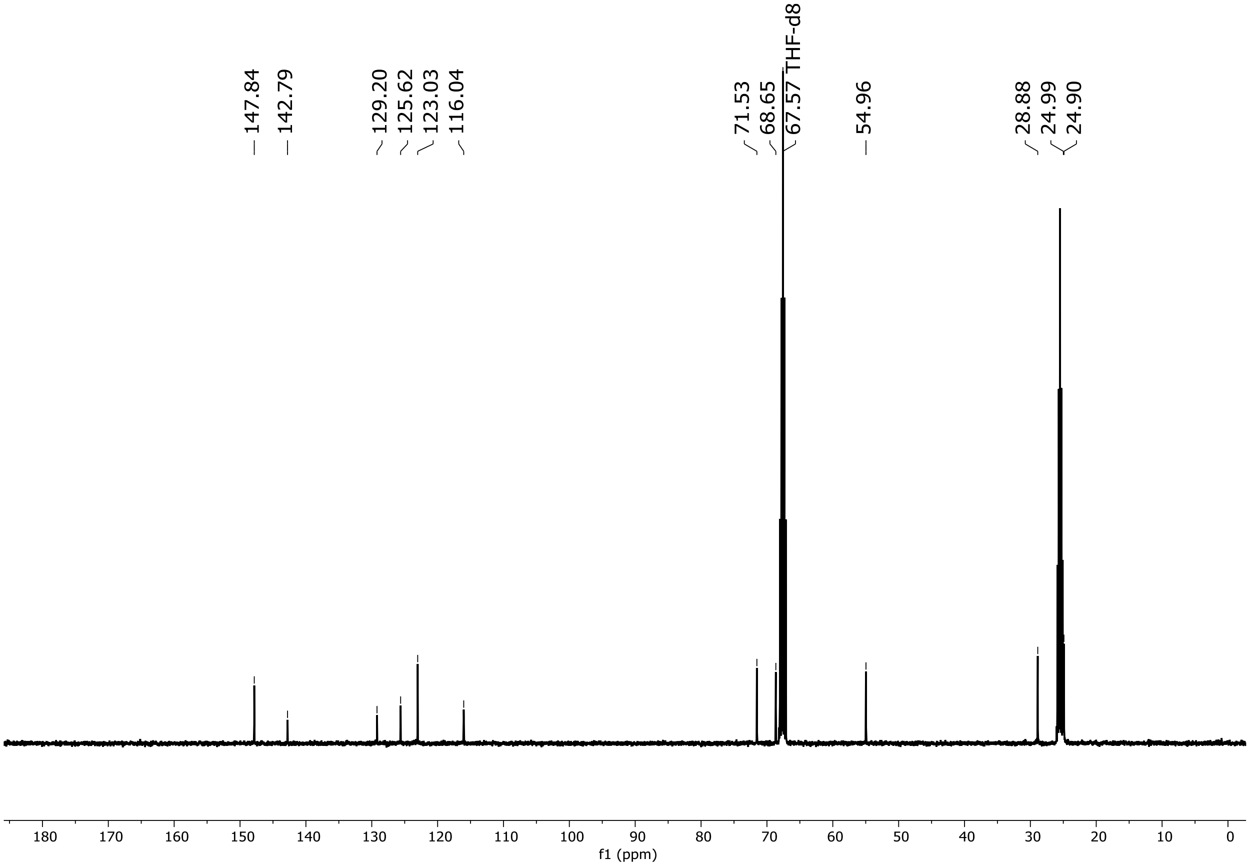

Figure S5. $^{13}C\{^1H\}$ NMR spectrum of compound **2.**

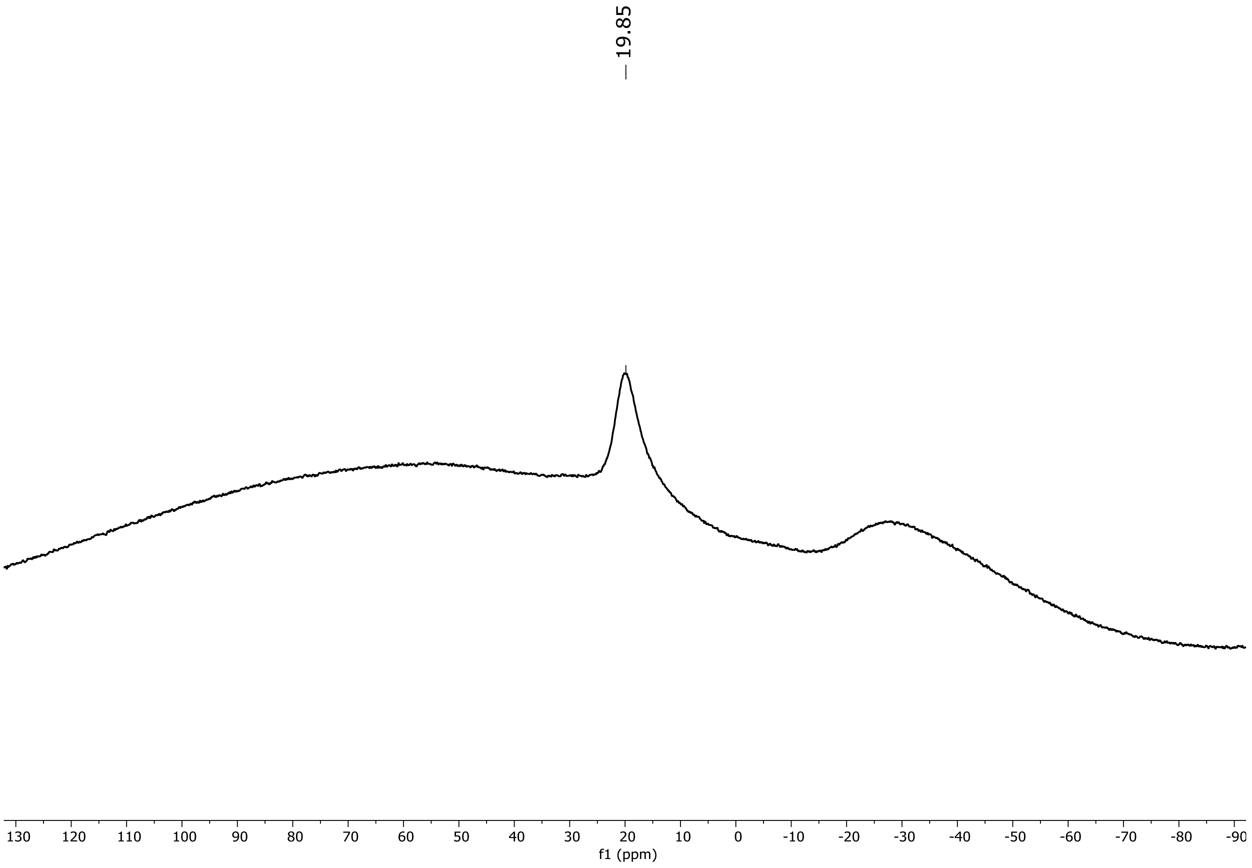

Figure S6. $^{11}B\{^{1}H\}$ NMR spectrum of compound **2.**

Preparation of [K(2.2.2-cryptand)][(HCDippN)$_2$BO]$_2$Al(C_6H_6)] [3]

A 2 mL C_6H_6 solution of **1** (100 mg, 0.1 mmol) was gradually added to [2.2.2]-cryptand (43.1 mg, 0.1 mmol) in 3 mL of C_6H_6. The solution was kept for two days without stirring, resulting in the formation of light-yellow crystals of compound **3**. The crystals were washed with hexane and allowed to dry under argon inside the glove box, with the sample vial kept open for five days (127 mg, 0.09 mmol, 84% yield). Alternatively, 25 mg of compound **2** was added to benzene, sonicated for 30 minutes, filtered, and left at room temperature without stirring. This process resulted in the formation of crystals of compound **3** after two days.

NB: Under high vacuum, **3** releases benzene and forms **2**. Compound **3** is insoluble in common organic solvents such as C_6H_6, toluene, hexane, etc., at 25 °C. However, upon sonication of **3** in C_6D_6, it shows partial solubility, suitable for ^{1}H NMR experiments. However, at 50 °C, **3** shows high solubility in C_6D_6, but a decrease in the (Al-C*H*) signal is observed in the ^{1}H spectra, suggesting an exchange between C_6H_6 and C_6D_6 (Figure S8). Intriguingly, addition of THF-d_8 to **3** leads to the de-coordination of the C_6H_6 moiety (Figure S9).

^{1}H NMR (400 MHz, C_6D_6, 297 K): δ = 1.42-1.47 (br, 48H, CH(CH_3)$_2$)), 1.99 (m,12H, NCH_2-crypt.), 2.54 (m, 2H, Al-C*H*), 2.97-3.04 [br, 24H, {(12H, NCH$_2$CH_2crypt)+ (12 H, OCH_2- crypt)}], 3.51-3.66 (br, 4H, $C^{2,3,5,6}$-*H*, C_6H_6), 3.79-3.88 (br, 8H, C*H*(CH$_3$)$_2$), 6.05 - 6.11 (s, 4H, NC*H*), 7.29 - 7.26 (Ar^{Dipp}-*H*, 12H). Due to the poor solubility of **3**, a suitable ^{13}C NMR of compound **3** was not observed.

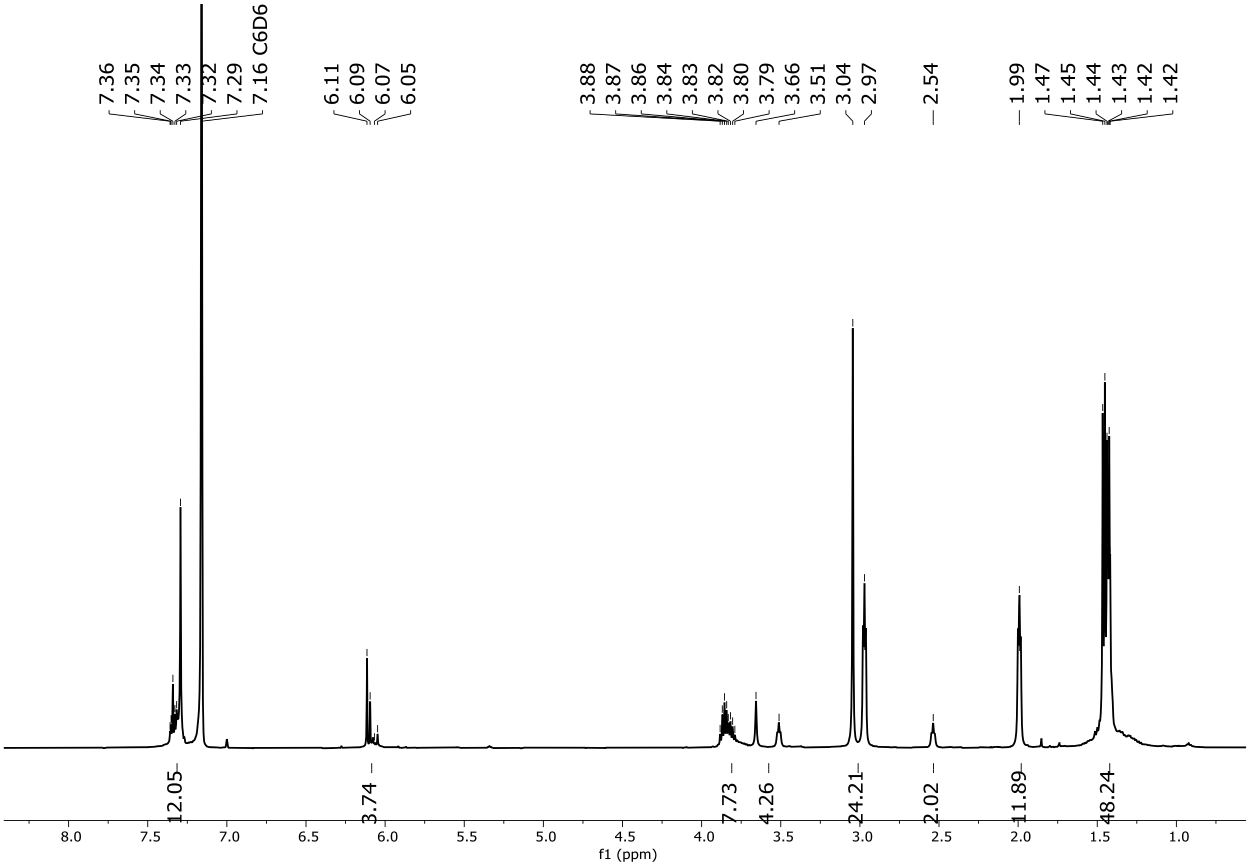

Figure S7. ^{1}H NMR spectrum of compound **3.**

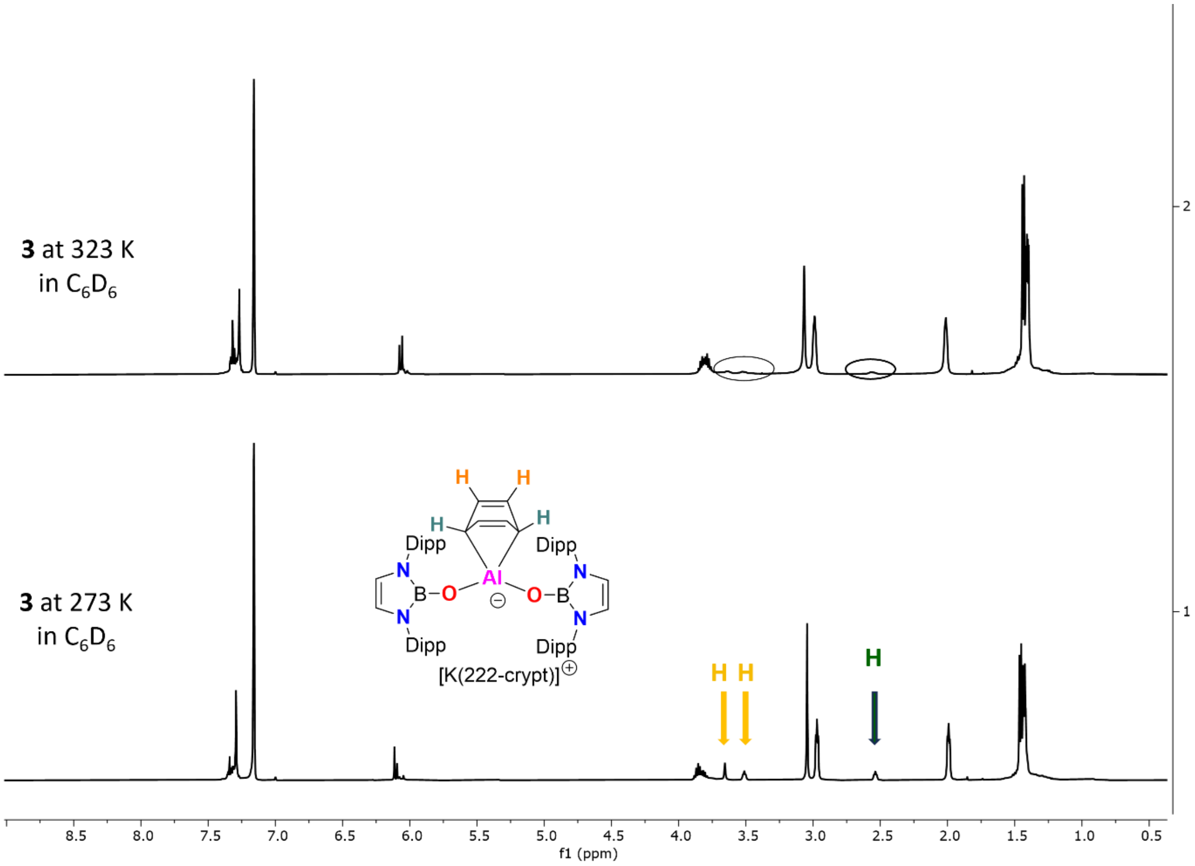

Figure S8. ^{1}H NMR spectrum of compound **3** at 273 K (bottom) and 323 K (top)**.**

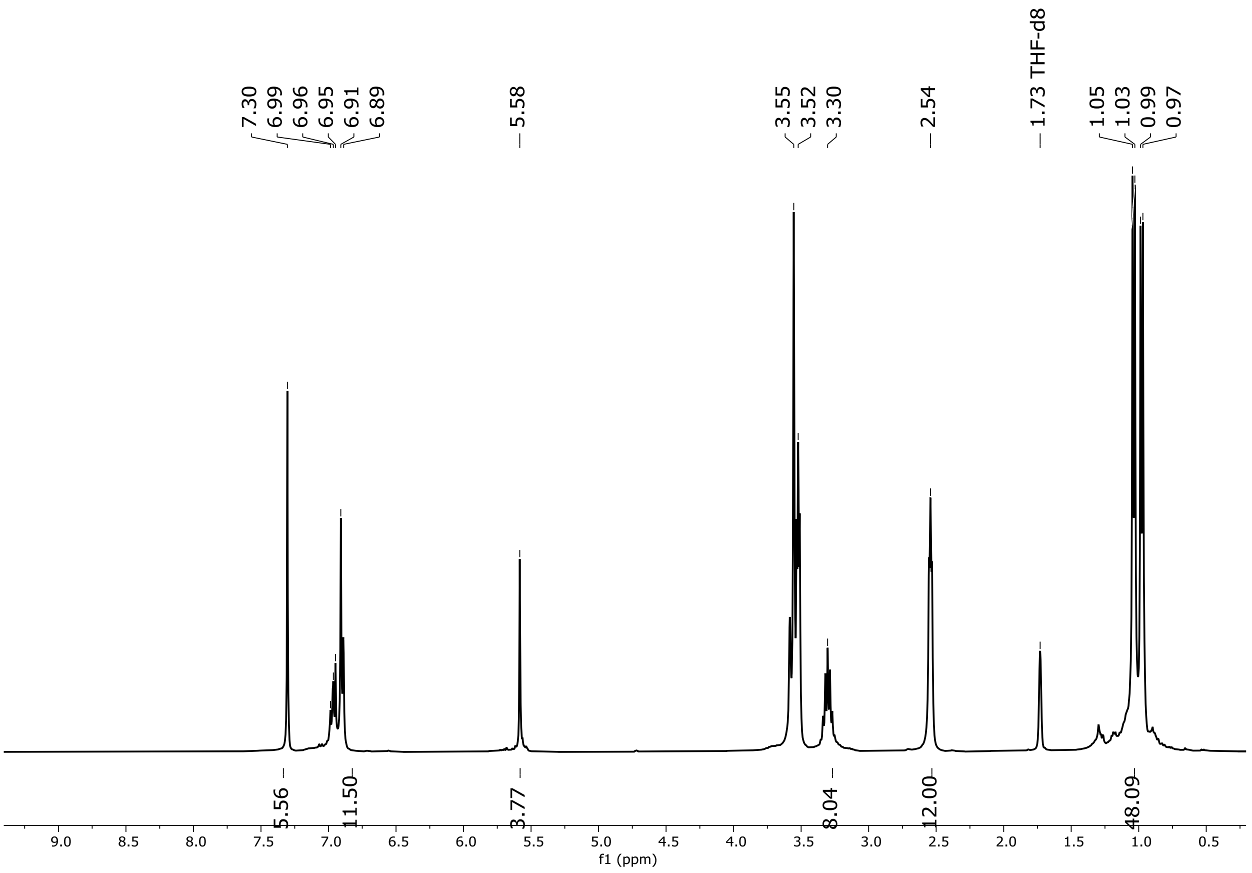

Figure S9. ^{1}H NMR spectrum of a sample of compound **3** in THF-d_8 (under these conditions benzene is displaced from compound **3**, yielding **2** and free benzene)

Preparation of [K(2.2.2-cryptand)][(HCDippN)$_2$BO]$_2$Al ($C_{12}H_6O_2$)] [4]

To a mixture of **3** (100 mg, 0.07 mmol) and acenaphthoquinone (13.6 mg, 0.07 mmol), benzene (3 mL) was added and stirred for five hours at room temperature to form a deep-blue solution. The solution was filtered, and all volatiles were removed under reduced pressure, yielding compound **4** as a deep-blue powder. The yield was 85 mg (0.54 mmol, 79%). Single crystals suitable for X-ray diffraction analysis were obtained from a concentrated solution (80 mg) of **4** in THF (1 mL) at -30 °C after 7 days. Alternatively, **4** could also be prepared from the reaction of **2** and acenaphthoquinone by mixing them 1:1 in benzene (Yield: 81%).

^{1}H NMR (400 MHz, THF-d_8, 297 K): δ = 0.96-1.0 (m, 48H, CH(C*H*$_3$)$_2$), 2.51 (m,12H, NCH$_2$-crypt.), 3.14 (br, 4H, C*H*(CH$_3$)$_2$),) 3.50-3.58 [br, (24H, NCH$_2$C*H*$_2$crypt, OC*H*$_2$crypt)+(4H, C*H*(CH$_3$)$_2$), spectrum overlap with THF-D8], 5.57 (s, 4H, NC*H*), 6.37-6.93 [m, (6H,Ar-H, acenaphthoquinone)+ (12H, Ar^{Dipp}-*H*)]. **^{13}C{^{1}H} NMR** (100 MHz, THF-d_8): δ = 24.1-24.9 (CH(*C*H$_3$)$_2$), 28.9 (*C*H(CH$_3$)$_2$), 54.8 (NCH$_2$-crypt.), 68.5 (NCH$_2$*C*H$_2$-crypt.), 71.3 (O*C*H$_2$-crypt.), 115.1 (Ar-C), 116.0 (N*C*H), 121.7 (Ar-*C*) 123.1 (Ar-*C*), 125.94 (Ar-*C*), 126.4 (Ar-*C*), 138.4 (Ar-*C*), 140.9 (Ar-*C*), 146.9 (Ar-*C*), 147.0 (Ar-*C*). **^{11}B{^{1}H} NMR** (128 MHz, THF-d_8): δ = 19.4. Anal. Calcd. [%] for $C_{82}H_{114}AlB_2KN_6O_{10}$: C, 68.80; H, 8.03; N, 5.87; Found: C, 68.72; H, 7.89; N, 5.74.

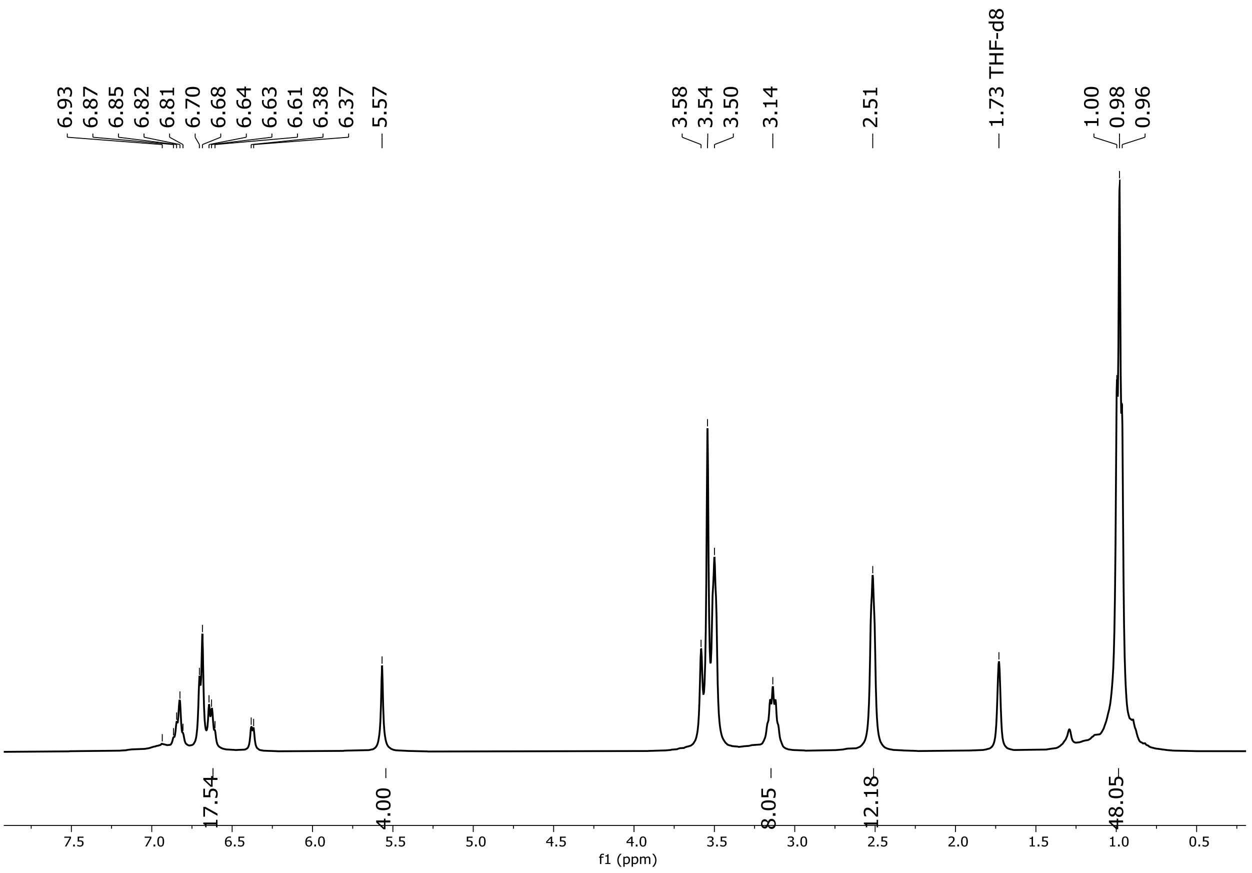

Figure S10. ^{1}H NMR spectrum of compound **4**.

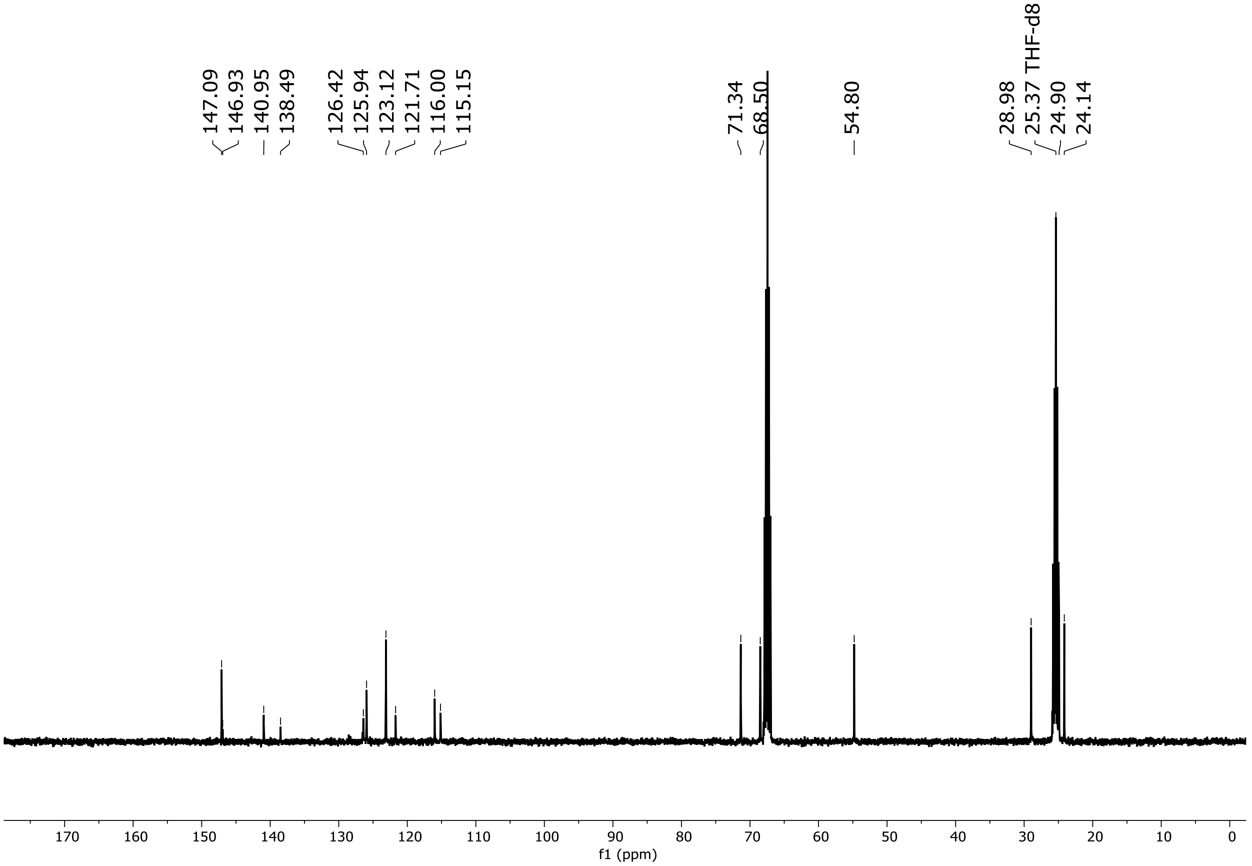

Figure S11. $^{13}C\{^{1}H\}$ NMR spectrum of compound **4**.

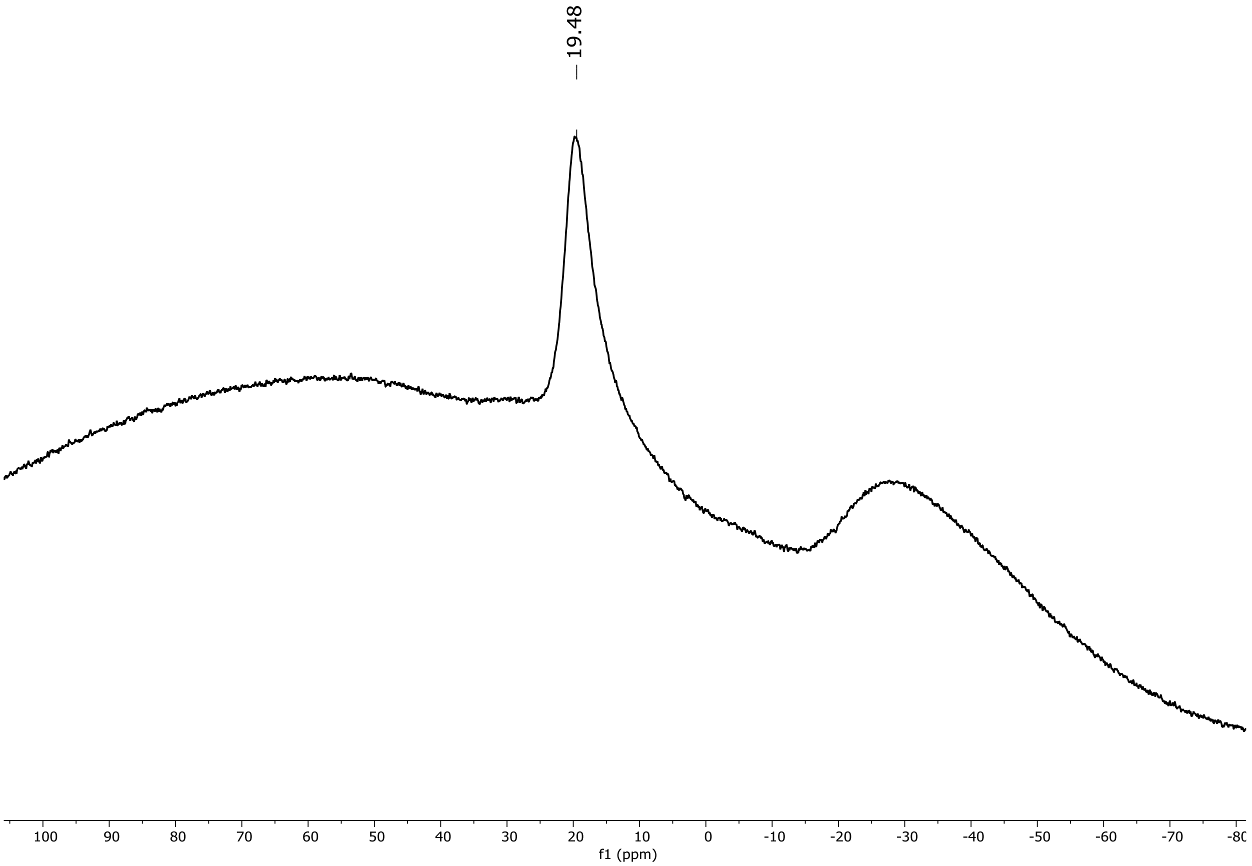

Figure S12. $^{11}B\{^1H\}$ NMR spectrum of compound **4**.

Preparation of [K(2.2.2-cryptand)][[(HCDippN)$_2$BO]$_2$Al(naphthalene)] [5]

To a mixture of **3** (100 mg, 0.07 mmol) and naphthalene (9.7 mg, 0.07 mmol), benzene (3 mL) was added and stirred for nine hours at room temperature. The solution was filtered, and all volatiles were removed under reduced pressure, yielding compound **5** as a colourless powder. The yield was 81 mg (0.05 mmol, 78%). X-ray quality crystals of **5** were obtained by recrystallizing this solid from a concentrated solution of warm benzene (60 mg in 0.5 mL benzene). **Alternative synthesis pathway:** To a solution of **2** (0.1 g, 0.08 mmol) in 2 mL of THF, a solution of naphthalene (10.2 mg, 0.08 mmol) in 1 mL of THF was added at room temperature. The reaction mixture was stirred for four hours at room temperature, after which volatiles were removed in vacuo, resulting in **5** as a colourless solid (Yield: 95 mg, 0.07 mmol, 86%).

^{1}H NMR (400 MHz, C_6D_6, 297 K): δ = 1.08-1.29 (br, 48H, CH(C*H*$_3$)$_2$), 1.67 (m,12H, NCH$_2$-crypt.), 2.69 (br, 2H, Al-C^{1,4}-*H*, naph), 2.64, 2.72 (br, 24H, OC*H*$_2$crypt.), 3.35-3.58 (br, 8H, C*H*(CH$_3$)$_2$), 5.18 (m, 2H, C^{2,3}-H, naph), 5.76-5.86 (s, 4H, NC*H*), 6.39-6.53 (m, 4H, C^{5,6,7,8}-H, naph), 6.95-7.18 (br, 12H, Ar^{Dipp}-*H*); **^{13}C{^{1}H} NMR** (100 MHz, C_6D_6): δ = 23.9-26.4 (CH(*C*H$_3$)$_2$), 28.4-28.5 (*C*H(CH$_3$)$_2$), 53.6 (N*C*H$_2$-crypt.), 67.4 (NCH$_2$*C*H$_2$-crypt.), 70.3 (O*C*H$_2$-crypt.), 116.7-117.0 (N*C*H), 119.5-119.7 (*C*^{5,6,7,8}-naph) 123.2-123.3 (Dipp-m-CH), 125.9-126.1 (C^2,^{3}-naph), 128.1 (Dipp-p-CH), 141.8-142.4 (Dipp-i-C), 147.9-148.0 (Dipp-o-C), 148.1 (C^{9,10}-naph). **^{11}B{^{1}H} NMR** (128 MHz, C_6D_6): δ = 19.1. Anal. Calcd. [%] for $C_{80}H_{116}AlB_2KN_6O_8$: C, 69.75; H, 8.49; N, 6.10. Found: C, 69.62; H, 8.36; N, 5.86.

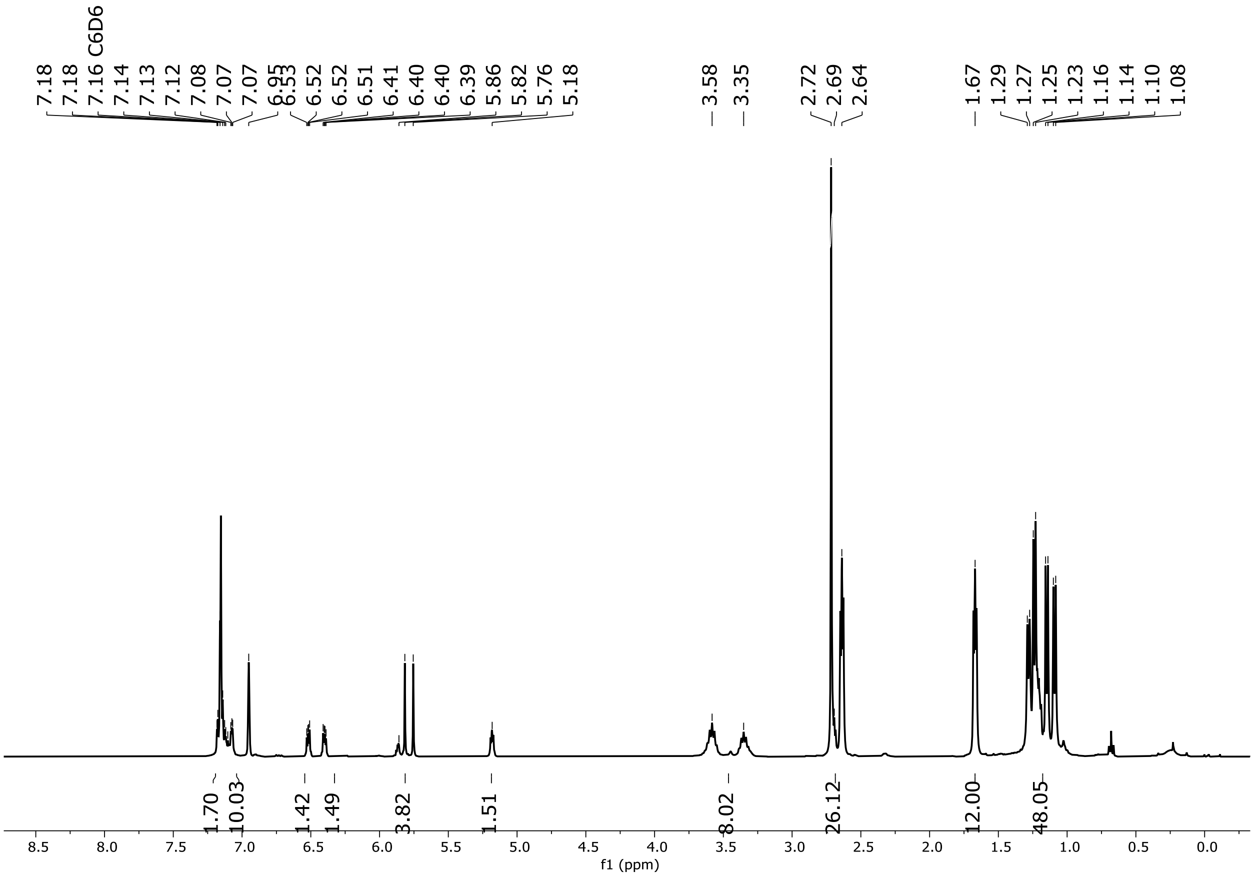

Figure S13. ^{1}H NMR spectrum of compound **5**.

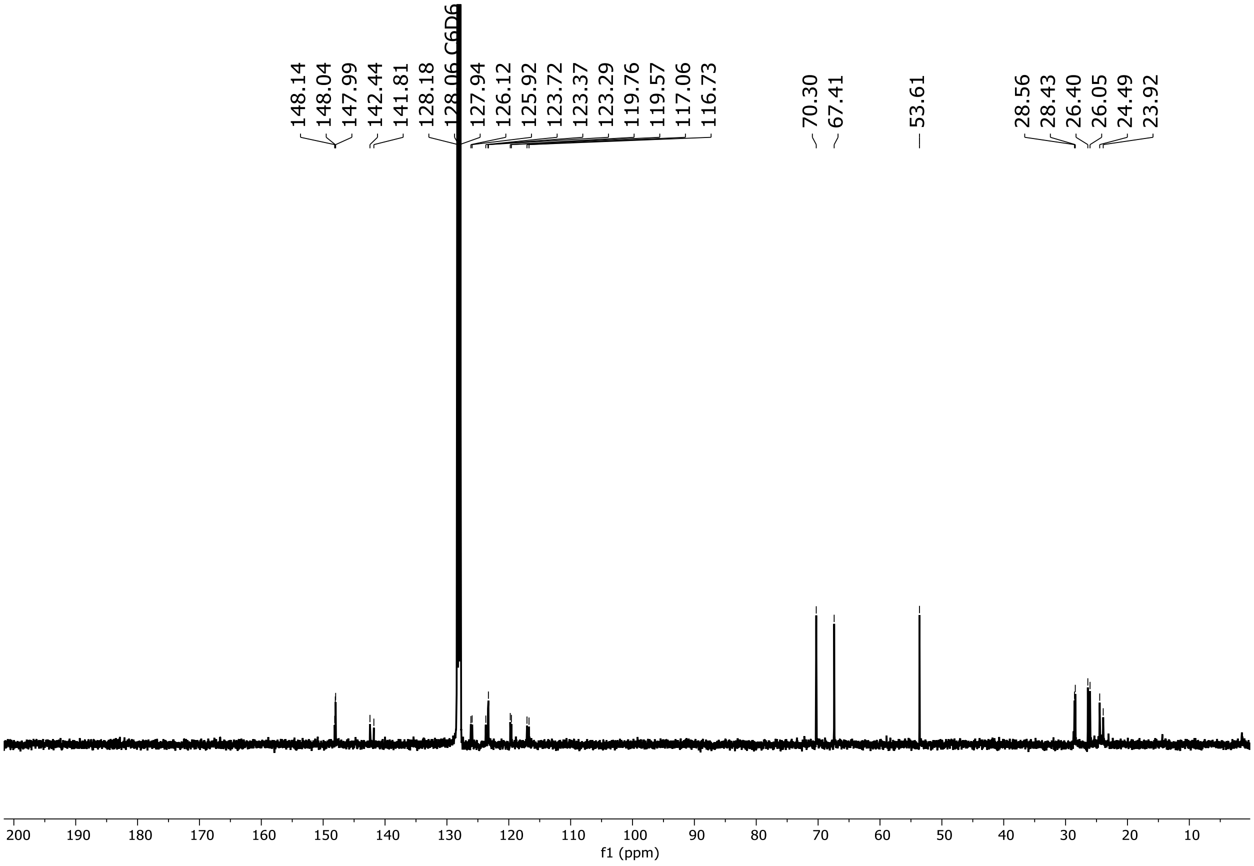

Figure S14. $^{13}C\{^{1}H\}$ NMR spectrum of compound **5**.

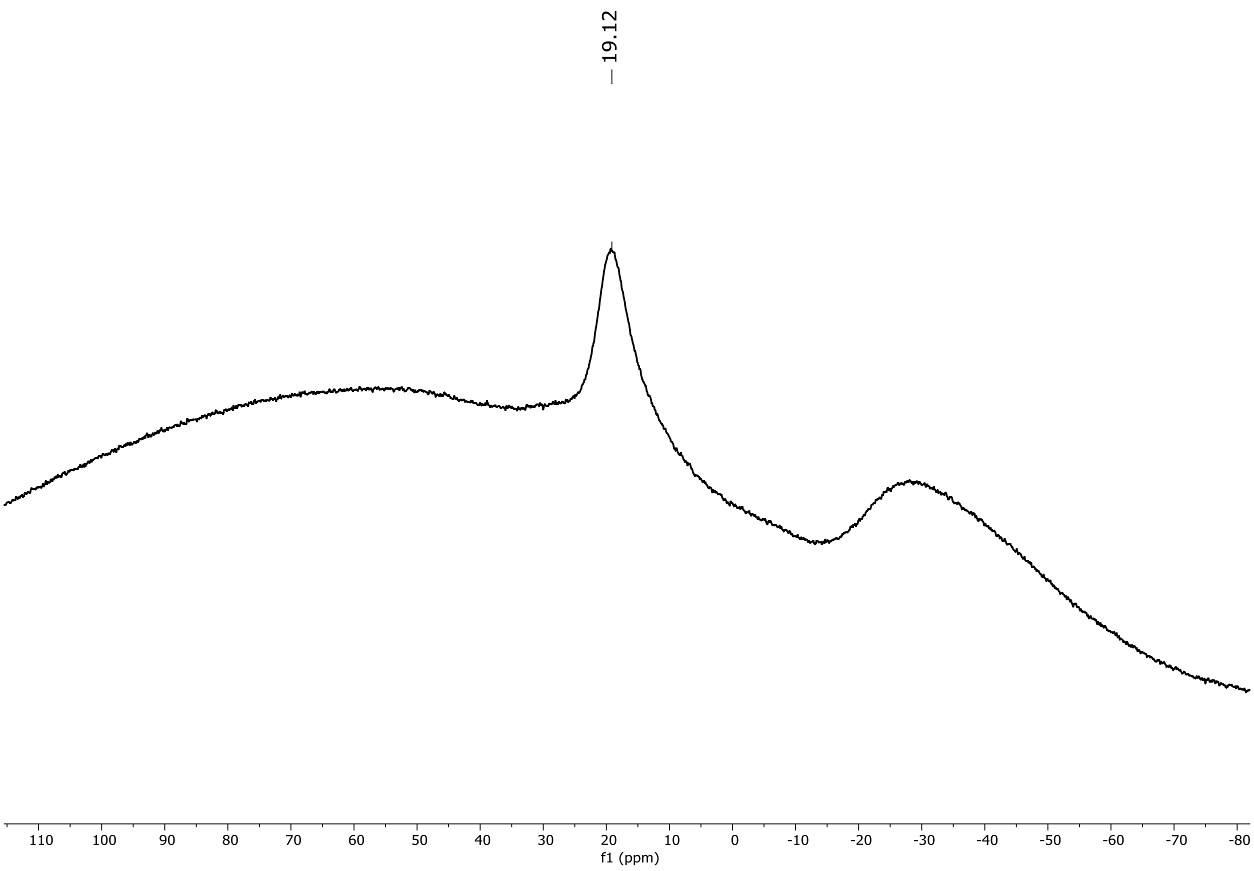

Figure S15. $^{11}B\{^1H\}$ NMR spectrum of compound **5**.

Preparation of $[[(HCDippN)_2BO]_2AlK(anthracene)]$ [6]

A benzene solution (2 mL) of **1** (0.1 g, 0.01 mmol) was added to anthracene (20.4 mg, 0.01 mmol) in 2 mL of benzene at room temperature. The colour of the solution rapidly changed from orange to light yellow. After stirring the solution for four hours, it was filtered via cannula filtration. The solution was then concentrated to 1 mL, and hexane (1 mL) was added to aid crystallization. After seven days, colourless crystals of compound **6** were obtained, which were suitable for X-ray diffraction analysis. For further analysis, the solvent was decanted, and the colourless crystals were washed once with hexane and dried in a high vacuum. Compound **6** was isolated as a colourless crystalline material (91 mg, 0.08 mmol, 75% yield).

NB: After crystallization, **6** is poorly soluble in common aromatic and aliphatic organic solvents. The addition of THF-d_8 to compound **6** or to a 1:1 mixture of **1** and anthracene leads to the rapid formation of the 1,4-activated product, presumably due to K+ sequestration by THF-d_8 (Figure S19).

^{1}H NMR (400 MHz, Toluene-d_8, 297 K): δ = 1.08-1.19 (m, 48H, CH(C*H*$_3$)$_2$), 2.98 (br 2H, C^{9,10}-*H*, anthra), 3.27 (br, 8H, C*H*(CH$_3$)$_2$), 5.76 (s, 4H, NC*H*), 6.28-6.37 (br, 8H, C^{1,2,3,4,5,6,7,8}-*H*, anthra), 6.96-7.07 (br, 12H, Ar^{Dipp}-*H*). **^{13}C{^{1}H} NMR** (100 MHz, C_6D_6): δ = 23.6-25.5 (CH(*C*H$_3$)$_2$), 26.2-28.5 (*C*H(CH$_3$)$_2$), 115.6-117.1 (N*C*H), 122.6-123.5 (Dipp-m-*C*H), 125.5-126.7 (*C*H, C^{1,4,5,8}-Anth), 127.5-128.0 (*C*H, C^{2,3,6,7}-Anth), 131.3-132.2 (Dipp-p-*C*H), 140.0 (Dipp-i-C), 141.1 (*C*, C^{4a,8a,9a,10a} -Anth), 147.8 (Dipp-o-C). **^{11}B{^{1}H} NMR** (128 MHz, C_6D_6): δ = 20.2. Anal. Calcd. [%] for $C_{66}H_{82}AlB_2KN_4O_2$: C, 75.42; H, 7.86; N, 5.33. Found: C, 74.98; H, 7.36; N, 5.13.

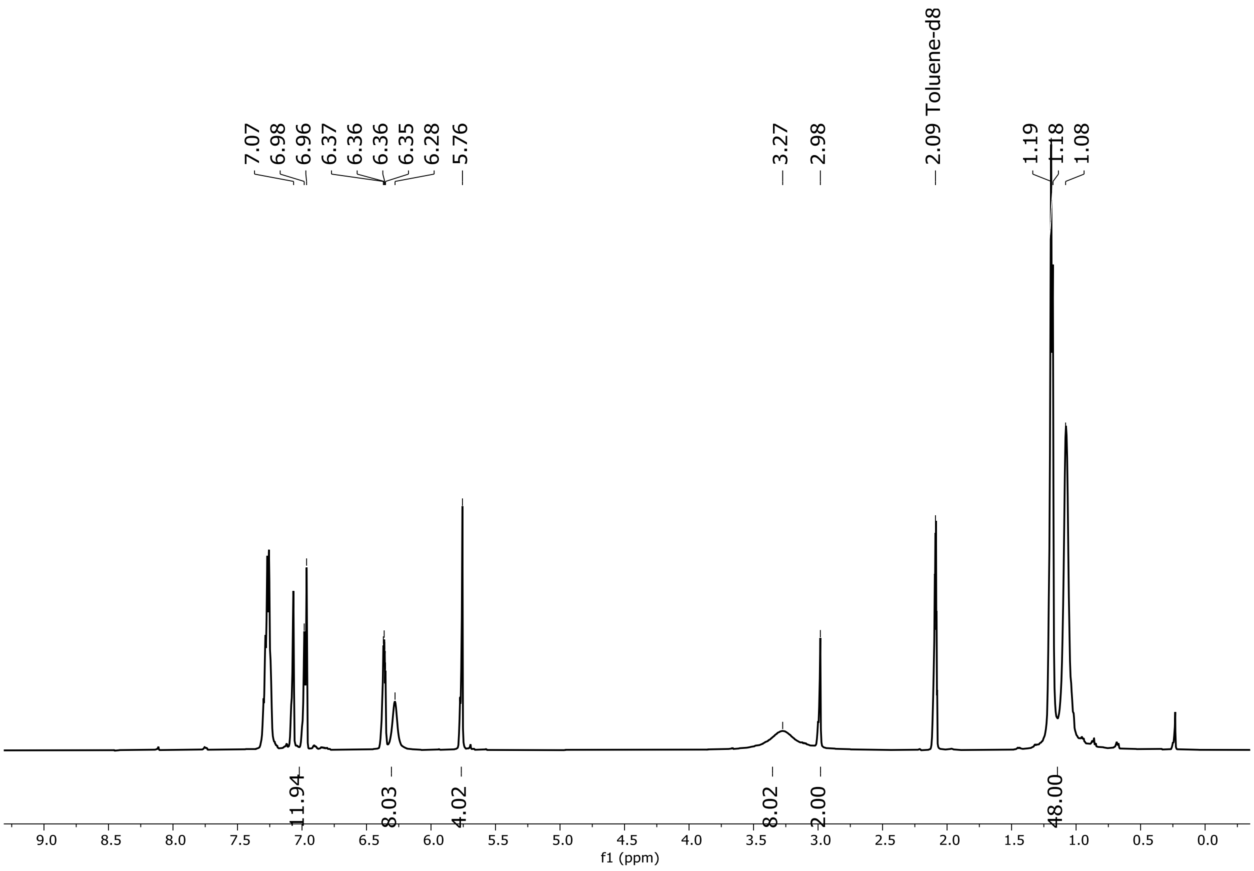

Figure S16. ^{1}H NMR spectrum of compound **6**.

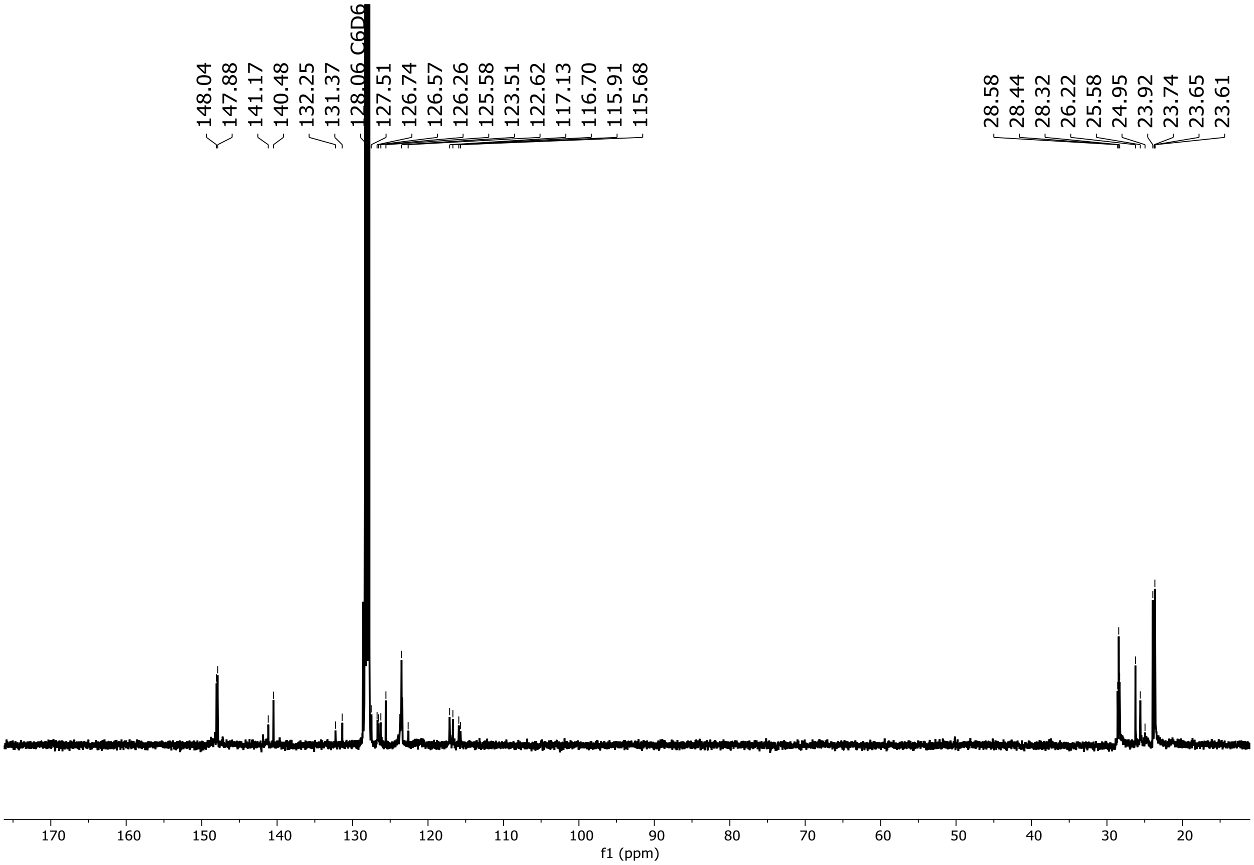

Figure S17. $^{13}C\{^{1}H\}$ NMR spectrum of compound **6**.

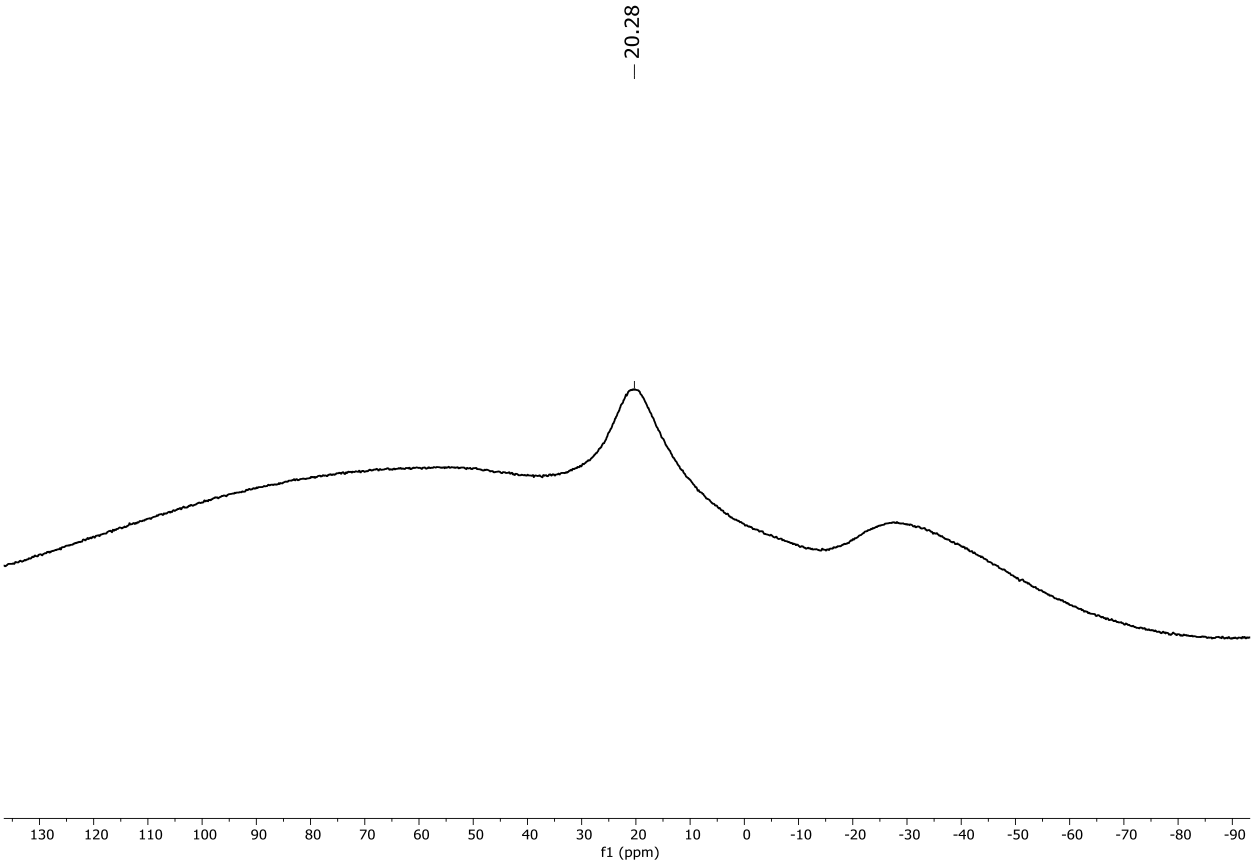

Figure S18. $^{11}B\{^{1}H\}$ NMR spectrum of compound **6**.

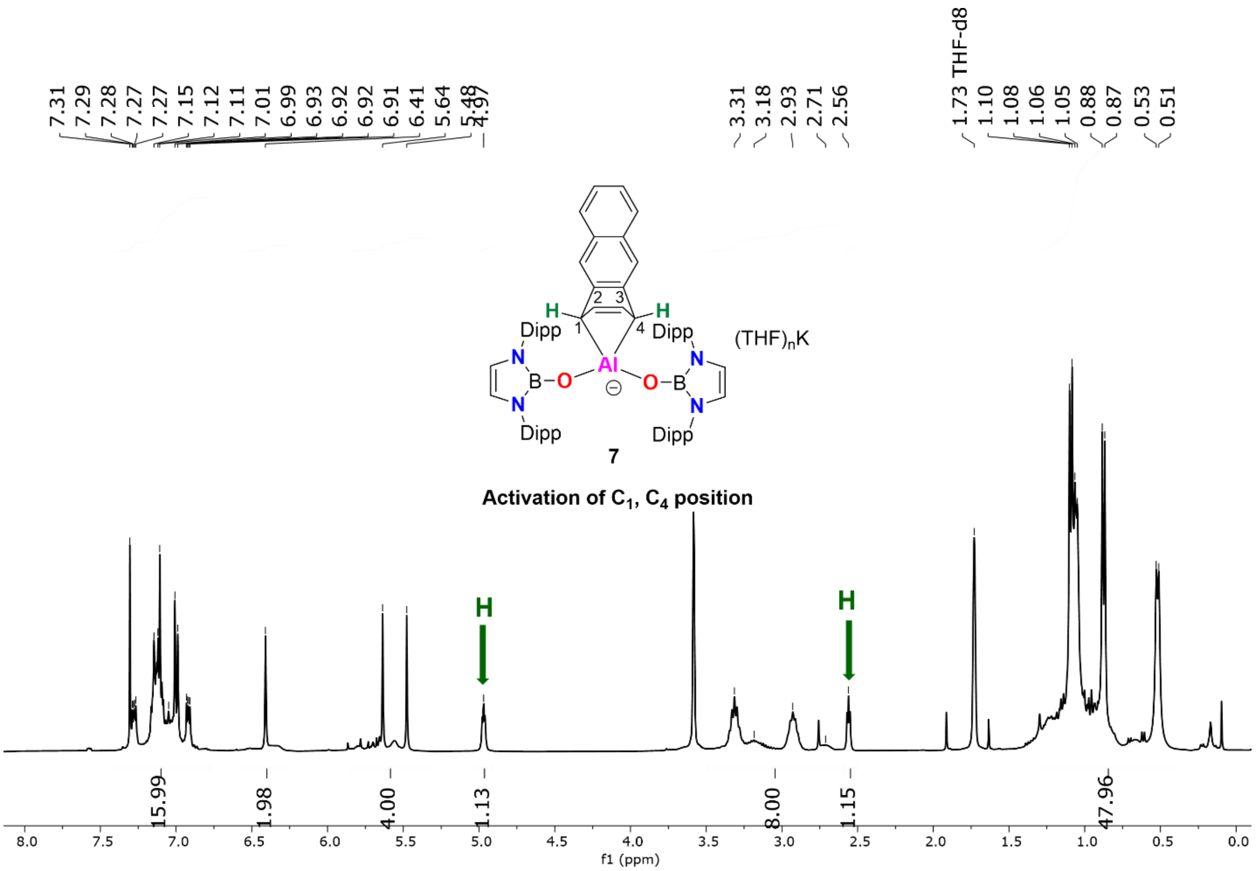

Figure S19. ^{1}H NMR spectrum of compound **6** in THF-d_8.

Preparation of [K(2.2.2-cryptand)][[(HCDippN)$_2$BO]$_2$Al(anthracene)] [7]

A THF (3 mL) solution of **2** (0.1 g, 0.08 mmol, 1.00 eq) was added to anthracene (14.2 mg, 0.08 mmol, 1.00 eq) in 2 mL of THF at room temperature. The color of the solution rapidly changed from orange to light yellow. After stirring the solution for one hour, it was filtered via cannula filtration. The solution was concentrated to 1 mL, and hexane (1 mL) was added to aid crystallization. After eight days, colorless crystals of compound **7** were obtained, which were suitable for X-ray diffraction analysis. For further analysis, the solvent was decanted, and the colorless crystals were washed once with hexane and dried in a high vacuum. Compound **7** was isolated as a colorless crystalline material (110 mg, 0.08 mmol, 75% yield). Alternatively compound **7** could be isolated via the treatment THF solution of **6** (1 eq) with 1 eq of [2.2.2-cryptand] or reaction of **3** (1 eq) with anthracene (1 eq).

^{1}H NMR (400 MHz, THF-d_8, 297 K): δ = 0.52-1.09 (m, 48H, CH(C*H*$_3$)$_2$), 2.36 (m,12H, NC*H*$_2$-crypt.), 2.45 (m, 2H, C^{1,4}-*H*, anthra), 2.94 (br, 4H, C*H*(CH$_3$)$_2$), 3.31-3.39 [br, (24H, OC*H*$_2$crypt, NCH$_2$C*H*$_2$crypt)+(4H, C*H*(CH$_3$)$_2$)], 4.85 (m, 2H, C^{2,3}-*H*, anthra), 5.44-5.60 (s, 4H, NC*H*), 6.31 (m, 2H, C^{9,10}-*H*, anthra), 6.78 (m, 2H, C^{5,8}-*H*, anthra), 7.06-7.14 (br, 12H, Ar^{Dipp}-*H*), 7.18 (m, 2H, C^{6,7}-*H*, anthra); **^{13}C{^{1}H} NMR** (100 MHz, THF-d_8): δ = 23.8-23.9 (CH(*C*H$_3$)$_2$), 28.4-28.7 (*C*H(CH$_3$)$_2$), 54.7 (NCH$_2$-crypt.), 68.4 (NCH$_2$*C*H$_2$-crypt.), 71.2 (O*C*H$_2$-crypt.), 114.9 (C^{9,10}-anthra), 116.6-117.0 (N*C*H), 120.1 (C^{5,8}-anthra), 123.2 (C^{2,3}-anthra), 125.9-126.2 (Dipp-m-CH), 126.6 (C^{6,7}-anthra), 132.4 (C^{4a,9a}-anthra), 141.7-142.5 (Dipp-i-C), 148.0 (Dipp-o-C), 150.9 (C^{8a,10a}-anthra). **^{11}B{^{1}H} NMR** (128 MHz, THF-d_8): δ = 21.4. Anal. Calcd. [%] for $C_{84}H_{118}AlB_2KN_6O_8$: C, 70.67; H, 8.33; N, 5.89. Found: C, 70.14; H, 8.09; N, 5.55.

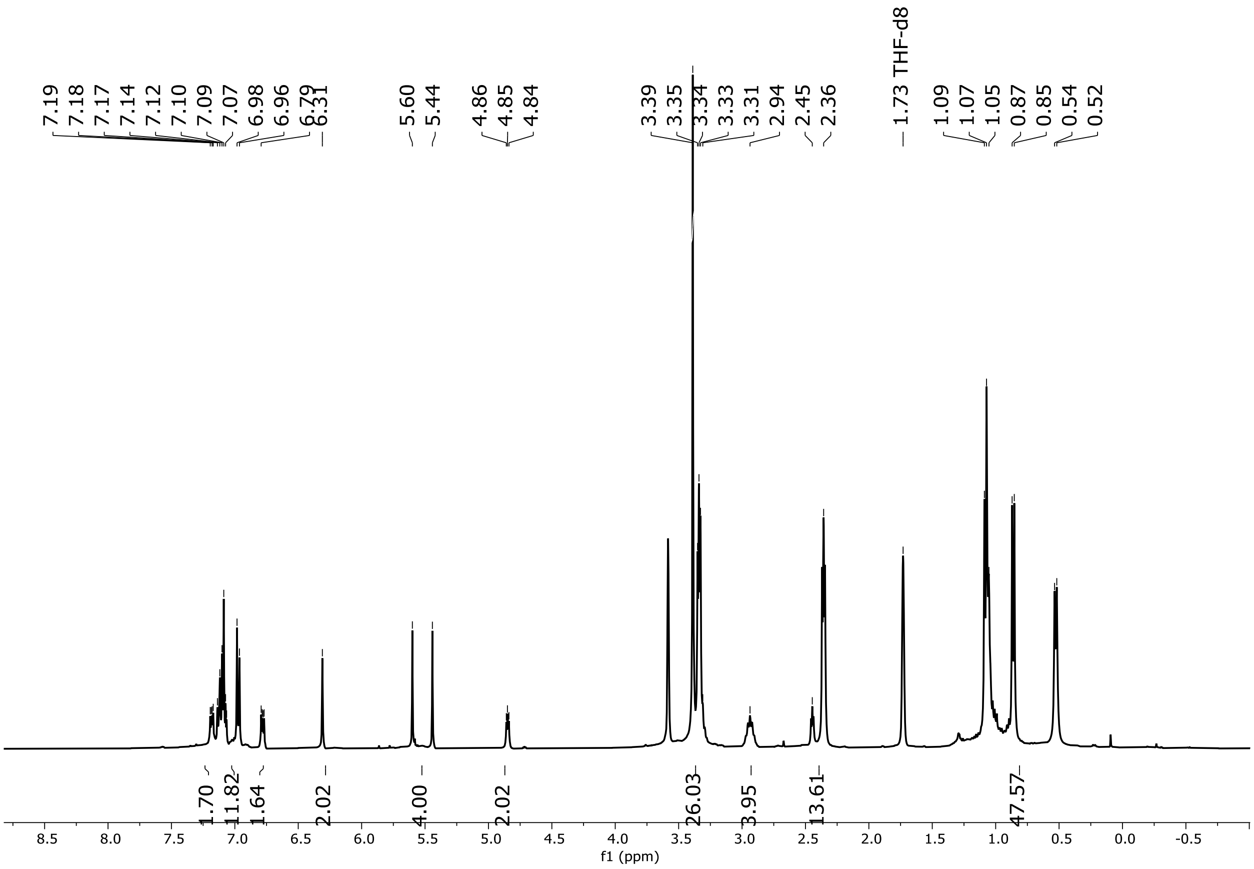

Figure S20. ^{1}H NMR spectrum of compound **7**.

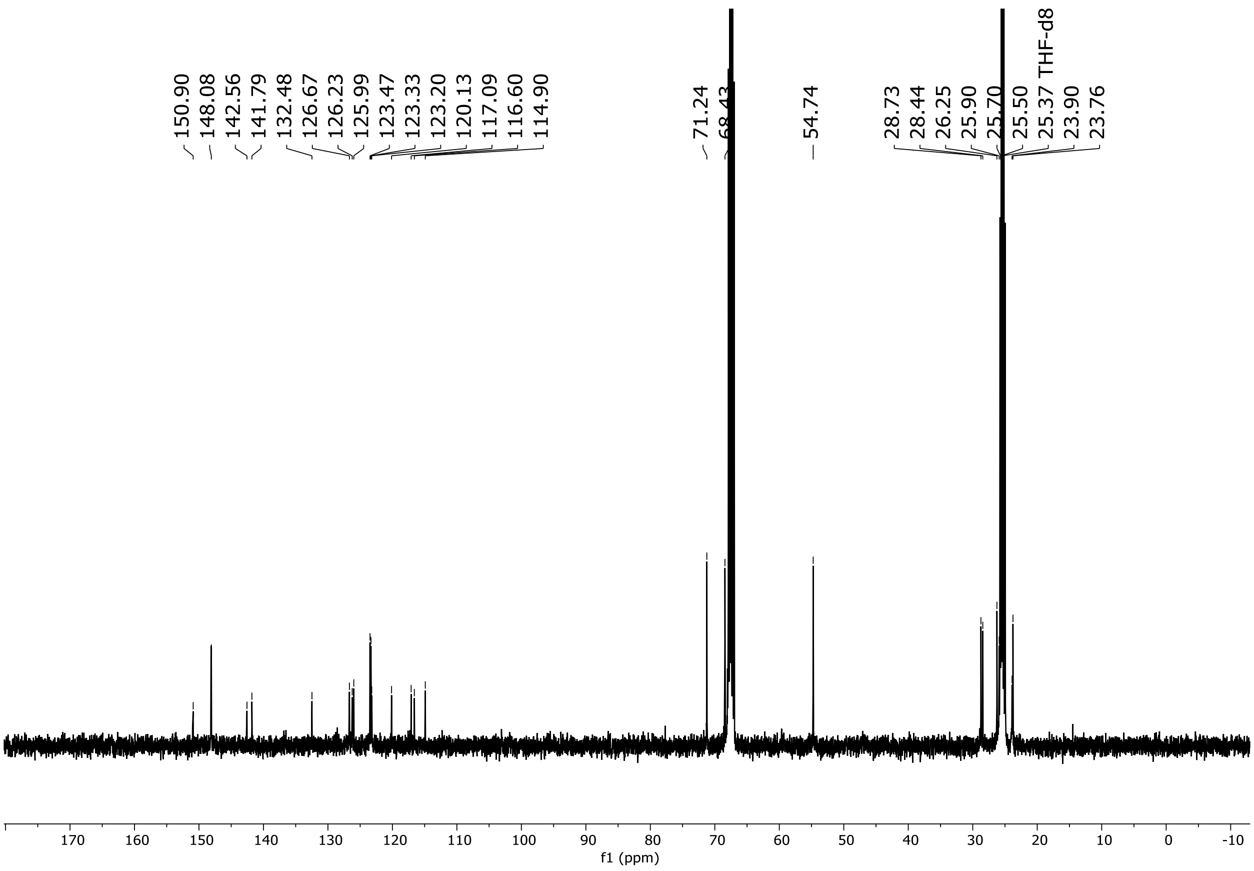

Figure S21. $^{13}C\{^{1}H\}$ NMR spectrum of compound **7**.

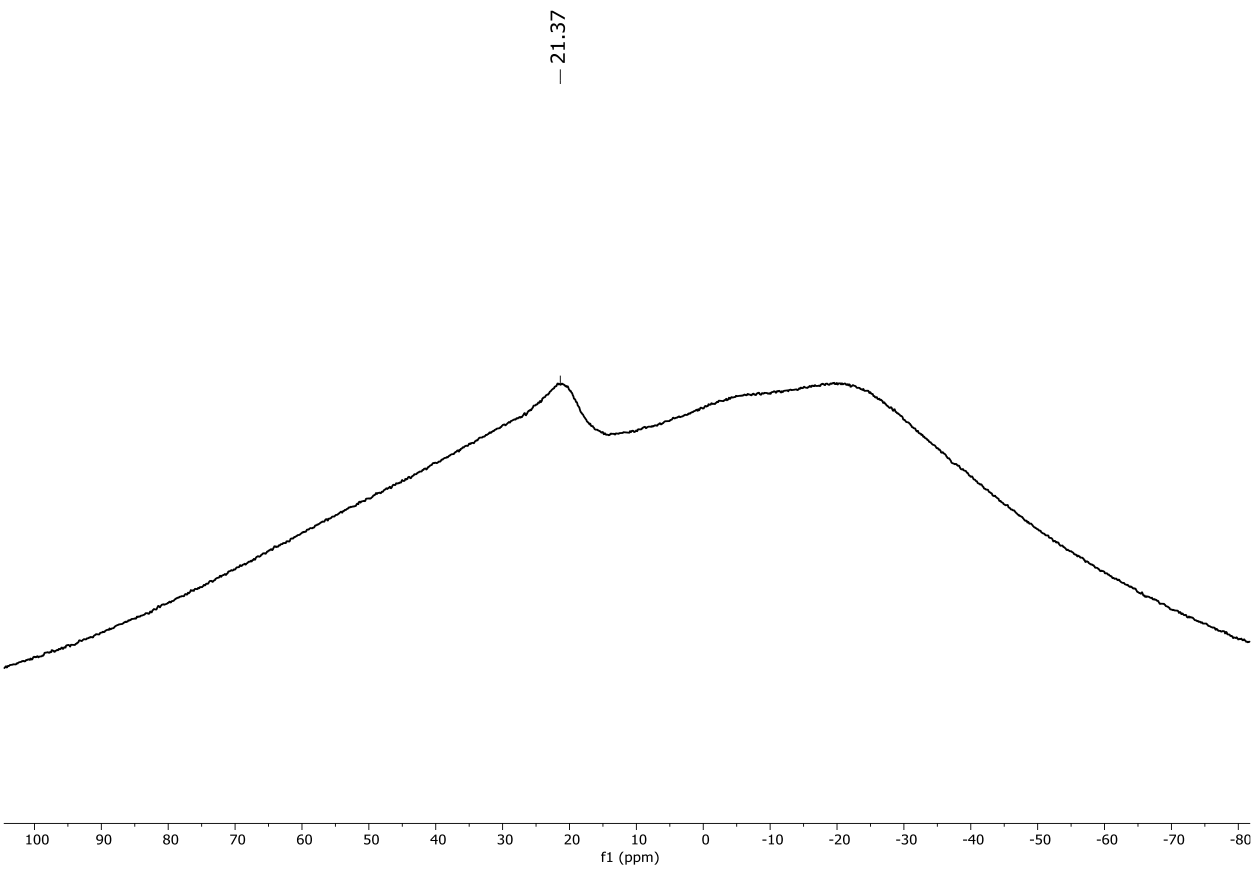

Figure S22. $^{11}B\{^{1}H\}$ NMR spectrum of compound **7**.

Details for DFT Calculations

All computational work reported here was carried out at the density functional theory (DFT) level, using Gaussian16 (Revision C.02).[3] The exchange correlation functional PBE1PBE[4-6] was employed in conjunction with the Def2-SVP[7, 8] basis set, Grimme's empirical dispersion correction (GD3BJ)[9] and an ultrafine integration grid for the optimization and frequency calculations for the mechanistic studies with benzene. The frontier orbital energies were obtained by optimizing the geometries of **1** and **2** using the method PBE1PBE-GD3BJ/Def2-TZVP. Single-point calculations were performed for the Def2-SVP optimized geometries at the PBE1PBE-GD3BJ/Def2-TZVP (SMD, solvent = thf)[6, 8, 10] level and corrected by 7.908 kJ/mol for the standard state in solution and thermal corrections. The given Gibbs free energies correspond to these corrected single point energies. The nature of the stationary points, minima and transition states, was confirmed by full frequency calculations at the Def2-SVP level, and are characterized by zero or one imaginary frequency, respectively.

Table S1. Calculated frontier molecular orbital energies for **1** and **2**. Calculated at the level PBE1PBE-GD3BJ/Def2-TZVP.

**Compound**	**HOMO, eV**	**LUMO, eV**	**LUMO+n (Al empty p), eV**	**HOMO-LUMO gap, eV**	**HOMO-LUMO+n gap, eV**	
**1**	-3.942	-0.709	0.401	3.233	4.343	LUMO+9
**2**	-2.772	-0.804	1.522	1.968	4.294	LUMO+25

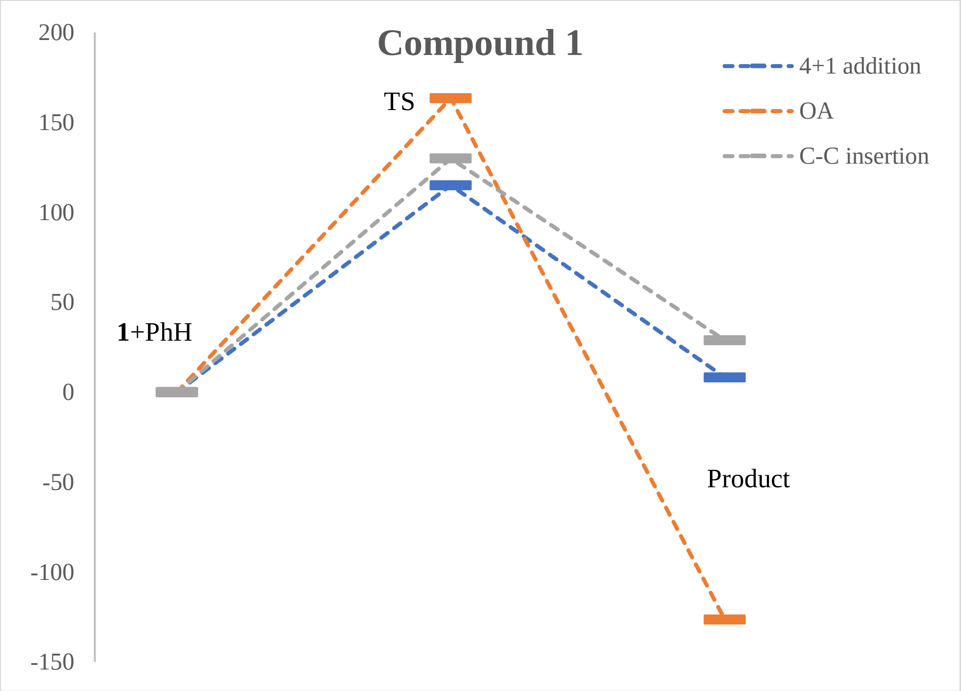

Figure S23. Gibbs free energies (kJ mol^{-1}) of activation of benzene by **1**. Calculated at the level PBE1PBE-GD3BJ/Def2-TZVP(SMD, solvent = thf)//PBE1PBE-GD3BJ/Def2-SVP.

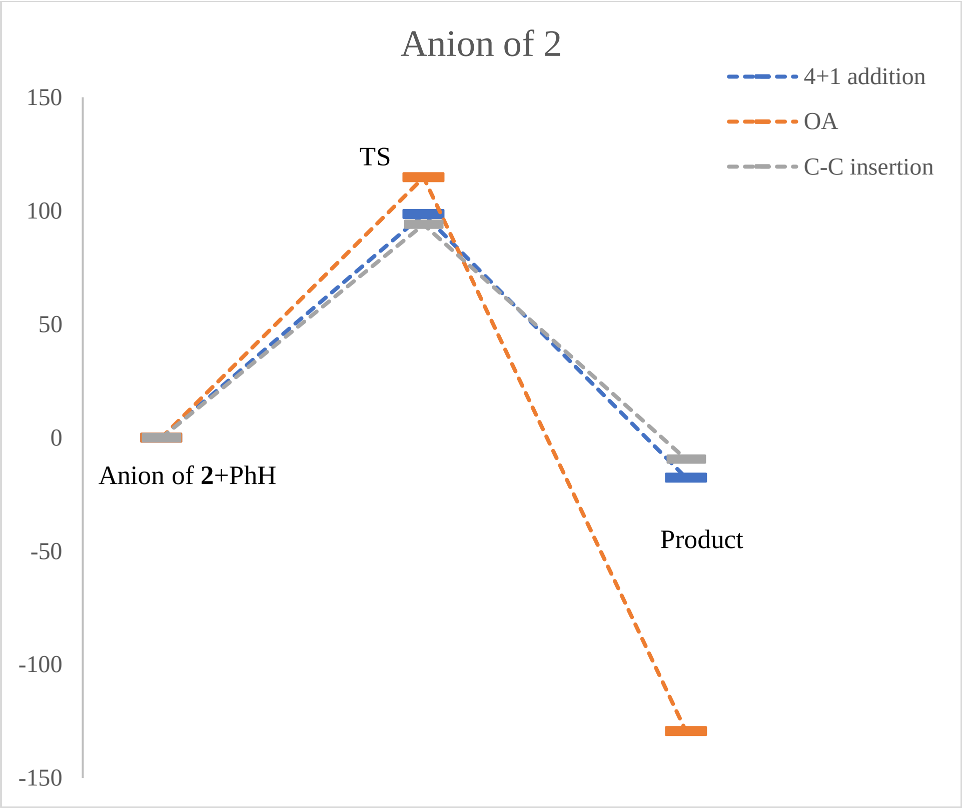

Figure S24. Gibbs free energies (kJ mol^{-1}) of activation of benzene by anion of **2**. Calculated at the level PBE1PBE-GD3BJ/Def2-TZVP(SMD, solvent = thf)//PBE1PBE-GD3BJ/Def2-SVP.

Table S2. Comparison of energies for the anthracene activation by **1** or the anion of **2**. Calculated at the level PBE1PBE-GD3BJ/Def2-TZVP(SMD, solvent = thf)//PBE1PBE-GD3BJ/Def2-SVP.

Compound	**1,4-activation, Gibbs free energy, G (a.u.)**	**9,10-activation, Gibbs free energy, G (a.u.)**	**$\Delta G_{9,10-1,4}$ (kJ mol^{-1})**
**1** + anthracene	-3822.657022	-3822.666444	-24.7
Anion of **2** + anthracene	-3222.944561	-3222.946739	-5.7

Optimised xyz-coordinates

Compound 1

134

scf done: -3284.704897

Al	-0.178434	0.772256	-1.200259
K	0.048265	-2.415372	0.102769
O	1.209685	-0.058565	-0.341041
N	3.497731	-0.924316	-0.802806
O	-1.389168	-0.185616	-0.220212
N	3.368043	1.259102	-0.168254
N	-3.447476	1.198984	0.295055
N	-3.474354	-1.002598	0.863173
C	3.176710	-2.267576	-1.074871
C	2.535388	-2.585001	-2.287423
C	2.968177	2.501620	0.385556
C	4.702571	0.897272	-0.336935
H	5.502341	1.604710	-0.186253
C	4.785300	-0.394279	-0.712520
H	5.662507	-0.971166	-0.959057
C	3.412424	-3.249863	-0.098318
C	2.126073	-3.900434	-2.490943
H	1.624607	-4.165518	-3.413471
C	3.002341	2.662973	1.775712
C	2.359310	-4.880523	-1.537845
H	2.042934	-5.901283	-1.721668
C	2.320341	-1.521843	-3.340288
H	2.034758	-0.602537	-2.820935

C	3.003839	-4.556914	-0.355362
H	3.184056	-5.329960	0.382212
C	4.002600	-2.871664	1.242508
H	4.645758	-2.002631	1.087752
C	-3.116593	2.489453	-0.189536
C	2.518779	3.528516	-0.458703
C	2.513357	3.352389	-1.959407
H	2.287816	2.299733	-2.156174
C	3.409352	1.533001	2.693365
H	3.793010	0.722461	2.070252
C	-3.142860	-2.360362	1.026024
B	2.540537	0.105936	-0.438689
C	2.599658	3.880904	2.313316
H	2.615427	4.022102	3.388558
C	2.110235	4.722539	0.124318
H	1.743354	5.523230	-0.505205
C	1.198394	-1.839041	-4.315897
H	1.443100	-2.682521	-4.968042
H	1.016553	-0.972345	-4.953848
H	0.261588	-2.061826	-3.798332
C	2.156379	4.903736	1.496220
H	1.834546	5.844432	1.929354
C	-2.569945	-2.791813	2.234692
B	-2.635204	0.005423	0.243600
C	4.848982	-3.970051	1.869353
H	4.246477	-4.828936	2.177230
H	5.346417	-3.590058	2.764413
H	5.617257	-4.326385	1.179900
C	-2.630379	3.451216	0.704053
C	-4.655636	0.890973	0.912977
H	-5.419650	1.637539	1.061402
C	-3.228856	2.752506	-1.562225
C	-3.311273	-3.242335	-0.053878
C	2.888053	-2.438414	2.194550
H	2.332875	-1.586222	1.794951
H	3.296883	-2.133395	3.160675
H	2.194850	-3.267518	2.378159
C	-4.679098	-0.413454	1.251368
H	-5.463861	-0.976448	1.730602
C	-2.140260	-4.112956	2.332405
H	-1.696090	-4.465415	3.256218
C	-2.272331	-4.985960	1.262218
H	-1.931266	-6.011253	1.353988
C	-2.416801	3.109078	2.160271
H	-3.127872	2.320604	2.420571
C	4.522935	1.937989	3.651899
H	4.200737	2.729579	4.333320
H	5.397749	2.301097	3.108240
H	4.830307	1.083914	4.261390
C	-2.857106	-4.552992	0.083009
H	-2.970440	-5.246555	-0.742819
C	-2.358638	-1.817063	3.370752
H	-3.071350	-1.001093	3.231100
C	-2.273455	4.697425	0.201405
H	-1.883699	5.454146	0.871428
C	3.895193	3.651580	-2.538599
H	4.177814	4.690440	-2.344514
H	3.895897	3.496824	-3.621017
H	4.659718	3.004603	-2.105409
C	-0.956844	-1.213675	3.292788
H	-0.192481	-1.990706	3.404325
H	-0.801226	-0.485311	4.091764
H	-0.799893	-0.692675	2.345330
C	-3.733338	1.690011	-2.511994
H	-3.385370	0.728174	-2.123429
C	-2.393710	4.978596	-1.147251
H	-2.103967	5.952785	-1.525138
C	3.625032	-1.242060	-4.084822
H	4.413131	-0.927330	-3.399371
H	3.477476	-0.444768	-4.817294
H	3.965787	-2.135155	-4.616658
C	-3.936725	-2.777931	-1.349186
H	-4.398456	-1.808000	-1.151833
C	2.193134	1.003542	3.448002
H	2.467746	0.159147	4.086331
H	1.418787	0.675430	2.752241
H	1.759454	1.779630	4.084325
C	-2.865236	4.013240	-2.019259
H	-2.930370	4.238780	-3.076149
C	1.441641	4.171504	-2.661449
H	0.455505	3.975939	-2.234973
H	1.406640	3.897921	-3.718651
H	1.646001	5.245004	-2.609913
C	-5.260368	1.655442	-2.530578
H	-5.667685	1.469589	-1.535486
H	-5.617900	0.864611	-3.196413
H	-5.660669	2.607304	-2.890999
C	-2.609895	-2.431518	4.740905
H	-3.593909	-2.902191	4.794091
H	-2.561540	-1.657220	5.509733
H	-1.860355	-3.185321	4.996479
C	-5.029104	-3.725439	-1.831605
H	-5.525291	-3.306576	-2.710040
H	-5.784115	-3.887035	-1.059371
H	-4.626399	-4.700481	-2.118631
C	-3.175079	1.826920	-3.920311
H	-3.566303	2.710921	-4.431632
H	-3.461564	0.957057	-4.517076
H	-2.084364	1.884014	-3.900266
C	-2.873766	-2.571987	-2.427134
H	-2.342120	-3.506163	-2.636318
H	-2.148762	-1.811630	-2.126956
H	-3.332171	-2.235257	-3.360227
C	-2.663963	4.280283	3.101173
H	-1.910849	5.063308	2.981362
H	-2.612706	3.941873	4.139043
H	-3.646623	4.728210	2.936656
C	-1.010026	2.548322	2.357009
H	-0.840308	1.673569	1.726595
H	-0.852446	2.254847	3.398772
H	-0.253074	3.291255	2.093806

Compound 2

196

scf done: -4552.158099

K	-6.18342	-0.03261	-0.10634
O	-4.71642	-0.79814	2.11559
O	-4.88511	-1.64749	-2.02553
O	-5.41200	1.93866	1.76484
O	-8.88499	0.92719	-0.48020
O	-5.11881	1.14070	-2.37293
O	-8.44765	-1.73644	0.43542
N	-5.76591	-2.98574	0.48005
N	-6.71233	2.93324	-0.71033
C	-4.82400	-2.10934	2.61694
H	-5.76516	-2.22014	3.17440
H	-4.00448	-2.30940	3.32074
C	-4.60088	0.16709	3.13824
H	-3.77376	-0.09458	3.81008
H	-5.52739	0.20214	3.72919

C	-9.85959	0.15661	0.17865
H	-10.86578	0.45067	-0.15277
H	-9.80258	0.31379	1.26534
C	-7.00570	-3.55991	0.97498
H	-7.13364	-3.26644	2.01875
H	-6.97078	-4.66393	0.95855
C	-4.30931	1.50945	2.53998
H	-4.11690	2.22068	3.35337
H	-3.40107	1.46529	1.92713
C	-8.23962	-3.11528	0.23122
H	-8.16765	-3.32530	-0.84468
H	-9.09979	-3.68279	0.61397
C	-4.71380	-3.09720	1.48104
H	-3.75380	-2.92753	0.99444
H	-4.67454	-4.11406	1.90917
C	-5.66748	2.32701	-2.89203
H	-6.62317	2.11215	-3.39164
H	-4.99529	2.75301	-3.64907
C	-5.81578	3.33974	-1.78296
H	-4.82371	3.50139	-1.36207
H	-6.13371	4.29985	-2.22625
C	-6.43085	3.68687	0.50438
H	-7.27700	3.58117	1.18607
H	-6.33129	4.76475	0.28751
C	-5.36248	-3.62325	-0.76712
H	-6.24518	-3.73748	-1.39970
H	-4.97156	-4.63955	-0.58345
C	-8.09879	3.08955	-1.11556
H	-8.19999	2.74495	-2.14684
H	-8.40255	4.15167	-1.10541
C	-4.33198	-2.86052	-1.55895
H	-3.42775	-2.65025	-0.97523
H	-4.02398	-3.47996	-2.41117
C	-4.59839	0.29191	-3.37004
H	-3.85756	0.83079	-3.97579
H	-5.40341	-0.05114	-4.03528
C	-9.65357	-1.29505	-0.13687
H	-10.50009	-1.86807	0.26789
H	-9.63748	-1.44101	-1.22652
C	-3.92610	-0.88362	-2.72662
H	-3.45465	-1.48874	-3.51216
H	-3.13292	-0.55081	-2.04361
C	-5.19859	3.23104	1.24490
H	-4.30407	3.23554	0.60907
H	-5.00479	3.93566	2.06579
C	-9.08368	2.30807	-0.28338
H	-8.99684	2.54806	0.78525
H	-10.10043	2.58634	-0.59539
Al	2.98936	0.05583	-1.43549
O	4.32605	0.18533	-0.25920
O	1.68372	-0.06698	-0.18828
N	0.22804	-2.12106	-0.21661
N	5.85651	2.17533	-0.24893
N	-0.63134	-0.22306	0.71903
N	6.78727	0.14117	0.18639
C	7.22395	2.32533	-0.03310
H	7.70617	3.28833	-0.09078
C	-0.74805	1.06925	2.77077
C	0.74322	-3.48493	-2.16632
C	5.11843	3.81696	-1.88007
C	-0.79842	1.01666	1.36420
C	1.03781	-3.08734	-0.85798
C	5.00836	3.24273	-0.60855
C	-0.40055	-2.87278	-2.94249
H	-0.99177	-2.27917	-2.24138

C	-1.05180	-2.37039	0.25068
H	-1.50110	-3.34824	0.15954
C	7.77215	1.12449	0.23608
H	8.80203	0.88728	0.45228
C	-1.56232	-1.25212	0.81287
H	-2.52791	-1.09619	1.26676
C	7.10329	4.28908	-3.35012
H	7.79862	3.85148	-4.07159
H	7.68154	4.65070	-2.49645
H	6.63238	5.15510	-3.82433
C	-0.30506	-0.14718	3.55247
H	-0.67344	-1.03493	3.03194
C	7.25109	-1.58142	1.82974
C	-0.96684	2.18360	0.60840
C	6.06422	3.25993	-2.91847
H	6.59558	2.42263	-2.46283
C	2.16006	-3.59064	-0.17851
C	4.29161	4.89013	-2.19915
H	4.36678	5.34504	-3.18122
C	2.45478	-3.15965	1.24011
H	2.26666	-2.08432	1.29161
C	1.57829	-4.41081	-2.78339
H	1.37570	-4.71697	-3.80391
C	-0.98116	2.28534	3.39962
H	-0.94571	2.34527	4.48096
C	4.04594	3.69335	0.31186
C	1.22446	-0.21169	3.54103
H	1.64153	0.62293	4.11092
H	1.62380	-0.15188	2.52710
H	1.57355	-1.14196	3.99597
C	2.68695	-4.91257	-2.13037
H	3.34212	-5.61540	-2.63288
C	2.97584	-4.49885	-0.84025
H	3.86144	-4.87784	-0.34617
C	7.12095	-0.55014	2.92874
H	7.44599	0.40998	2.52088
C	-1.18644	3.38407	1.28179
H	-1.31236	4.29674	0.70881
C	-0.82326	-0.19720	4.98189
H	-0.54541	-1.14770	5.44314
H	-1.91187	-0.10403	5.03152
H	-0.39339	0.59562	5.59966
C	3.35977	5.36237	-1.29563
H	2.71620	6.19415	-1.56169
C	7.02465	-1.21375	0.49567
C	5.29064	2.71232	-4.11474
H	4.75134	3.50847	-4.63685
H	4.56228	1.96130	-3.79675
H	5.97597	2.24769	-4.82961
C	3.92437	3.03637	1.66889
H	4.03275	1.96035	1.50852
B	0.57166	-0.72789	0.05817
C	3.23181	4.75641	-0.05550
H	2.47732	5.11392	0.63440
C	-1.21614	3.43613	2.66224
H	-1.38128	4.38087	3.16863
B	5.51477	0.76668	-0.13016
C	6.99132	-2.16964	-0.53013
C	7.50555	-2.91834	2.11438
H	7.69077	-3.22429	3.13722
C	0.12974	-1.92434	-4.01448
H	0.69970	-2.47138	-4.77074
H	0.79813	-1.17461	-3.58152
H	-0.69524	-1.41210	-4.52149
C	-1.31225	-3.93542	-3.54581

H	-2.14431	-3.47195	-4.08511
H	-1.72201	-4.59503	-2.77579
H	-0.77631	-4.56145	-4.26311
C	6.71829	-1.74742	-1.95590
H	5.96060	-0.96080	-1.91654
C	7.23292	-3.49812	-0.19682
H	7.20853	-4.25501	-0.97130
C	1.50917	-3.85595	2.21866
H	0.46343	-3.63753	1.99511
H	1.71310	-3.52817	3.24200
H	1.65006	-4.94031	2.17884
C	7.50222	-3.86987	1.10937
H	7.69469	-4.91038	1.34779
C	2.57205	3.24865	2.32975
H	2.52296	2.67118	3.25524
H	1.75267	2.91841	1.68881
H	2.40185	4.29651	2.59531
C	3.89952	-3.37268	1.65588
H	4.16195	-4.43302	1.71104
H	4.05871	-2.95043	2.64955
H	4.59395	-2.88081	0.97362
C	7.97260	-1.15118	-2.59201
H	8.34402	-0.30228	-2.01553
H	7.75520	-0.80297	-3.60569
H	8.76817	-1.90035	-2.65203
C	7.97753	-0.84191	4.15168
H	7.92129	-0.00584	4.85333
H	9.02610	-0.99006	3.88241
H	7.63699	-1.73348	4.68576
C	-2.17280	2.63505	-1.54343
H	-2.40152	3.67230	-1.27600
H	-3.01222	2.00716	-1.23802
H	-2.09382	2.58968	-2.63383
C	5.05082	3.49211	2.59485
H	4.98933	4.57049	2.77097
H	6.03056	3.26893	2.17021
H	4.97756	2.98585	3.56174
C	6.14539	-2.85634	-2.82397
H	6.87180	-3.65425	-3.00784
H	5.85608	-2.44579	-3.79393
H	5.25182	-3.29274	-2.37298
C	5.65114	-0.39580	3.32218
H	5.28745	-1.31044	3.79948
H	5.02330	-0.20041	2.45069
H	5.52739	0.42875	4.02997
C	0.30393	3.02282	-1.37485
H	0.41262	2.95048	-2.45948
H	1.23893	2.68089	-0.93217
H	0.17001	4.07716	-1.11528
C	-0.87135	2.17744	-0.89845
H	-0.68450	1.14849	-1.21060

Mechanistic studies:

Compound 1

134

scf done: -3284.74224982

Al	-0.090860	0.923064	-0.983654
K	-0.091260	-2.373434	0.166218
O	1.240189	-0.141135	-0.243945
N	3.428637	-1.155999	-0.862244
O	-1.359525	-0.045988	-0.030556
N	3.496767	1.017310	-0.153611
N	-3.385903	1.417726	0.399101
N	-3.536717	-0.798474	0.927841
C	2.965144	-2.447152	-1.179487
C	2.202991	-2.629780	-2.358909
C	3.192979	2.284757	0.406440
C	4.792107	0.566070	-0.412003
H	5.656619	1.213606	-0.281145
C	4.758042	-0.723592	-0.831069
H	5.582450	-1.360207	-1.146257
C	3.167194	-3.509242	-0.269751
C	1.646791	-3.891794	-2.600302
H	1.052948	-4.053514	-3.501732
C	3.216568	2.432808	1.807017
C	1.846943	-4.949455	-1.714360
H	1.412360	-5.929515	-1.926738
C	2.022706	-1.484662	-3.336228
H	1.884554	-0.570502	-2.737227
C	2.606776	-4.759023	-0.562935
H	2.756805	-5.593638	0.125085
C	3.875286	-3.259528	1.049044
H	4.614603	-2.462124	0.879688
C	-2.979730	2.661688	-0.147366
C	2.833642	3.356196	-0.438657
C	2.842535	3.195390	-1.945869
H	2.596473	2.140513	-2.150393
C	3.510453	1.253864	2.713646
H	3.947517	0.461610	2.087297
C	-3.265247	-2.173387	1.071446
B	2.572940	-0.067684	-0.411214
C	2.890614	3.677763	2.352749
H	2.897288	3.809639	3.437502
C	2.505662	4.579641	0.152692
H	2.212262	5.418501	-0.480543
C	0.788605	-1.613024	-4.219187
H	0.869760	-2.444258	-4.938408
H	0.650981	-0.688281	-4.798373
H	-0.125782	-1.758619	-3.622735
C	2.537665	4.743890	1.533688
H	2.276988	5.709405	1.974692
C	-2.696834	-2.646762	2.275373
B	-2.618521	0.189111	0.381099
C	4.620080	-4.474787	1.588650
H	3.933922	-5.277511	1.903116
H	5.211138	-4.193841	2.473369
H	5.308634	-4.893225	0.839180
C	-2.356397	3.611775	0.684720
C	-4.656439	1.145013	0.904067
H	-5.406293	1.926547	1.009350
C	-3.138139	2.885424	-1.529629
C	-3.484982	-3.041185	-0.021761
C	2.875747	-2.722747	2.077437
H	2.395376	-1.798599	1.722706
H	3.375696	-2.483361	3.028542
H	2.096644	-3.474546	2.295749
C	-4.751852	-0.169403	1.224092
H	-5.591742	-0.712384	1.652278
C	-2.321747	-3.994020	2.354377
H	-1.877973	-4.377942	3.275993
C	-2.508916	-4.853586	1.272929
H	-2.211468	-5.902460	1.350725
C	-2.148426	3.325877	2.158717
H	-2.936821	2.618517	2.461416
C	4.523107	1.580985	3.807299
H	4.136218	2.329437	4.516919
H	5.458457	1.974745	3.381767

H	4.766419	0.677631	4.388669
C	-3.090217	-4.379838	0.098371
H	-3.243526	-5.064272	-0.739353
C	-2.428777	-1.692205	3.422349
H	-3.104760	-0.834724	3.284501
C	-1.879879	4.789389	0.103168
H	-1.373636	5.531201	0.724007
C	4.241169	3.464405	-2.506047
H	4.550383	4.503086	-2.304969
H	4.257617	3.310636	-3.596856
H	4.991882	2.795703	-2.060923
C	-0.997494	-1.155245	3.340556
H	-0.261605	-1.970784	3.452619
H	-0.800959	-0.426587	4.141595
H	-0.817452	-0.641043	2.384763
C	-3.809583	1.853106	-2.413279
H	-3.764054	0.895702	-1.871054
C	-2.033354	5.026518	-1.258658
H	-1.650371	5.951154	-1.697470
C	3.289393	-1.294677	-4.173090
H	4.166609	-1.115711	-3.534932
H	3.178945	-0.428343	-4.843575
H	3.488645	-2.184075	-4.792648
C	-4.073878	-2.517009	-1.317108
H	-4.581406	-1.570835	-1.075050
C	2.204584	0.712166	3.297138
H	2.384828	-0.185057	3.910628
H	1.499749	0.451760	2.494386
H	1.717336	1.465995	3.935873
C	-2.664442	4.085722	-2.064142
H	-2.769271	4.277374	-3.134068
C	1.791467	4.043112	-2.651920
H	0.789851	3.852038	-2.236999
H	1.763837	3.787211	-3.722341
H	2.011276	5.120955	-2.581772
C	-5.287438	2.191511	-2.614506
H	-5.816270	2.260558	-1.652309
H	-5.787216	1.420330	-3.222770
H	-5.401629	3.157942	-3.131881
C	-2.715732	-2.302447	4.790332
H	-3.732636	-2.719801	4.841662
H	-2.622860	-1.535075	5.573705
H	-2.006557	-3.106896	5.043288
C	-5.111014	-3.459921	-1.920251
H	-5.594644	-2.984964	-2.787313
H	-5.894890	-3.716618	-1.191855
H	-4.660137	-4.399669	-2.277415
C	-3.091112	1.654795	-3.744572
H	-3.175627	2.539667	-4.395510
H	-3.532145	0.807183	-4.293108
H	-2.021166	1.448877	-3.581567
C	-2.963903	-2.192875	-2.320753
H	-2.387936	-3.098183	-2.579248
H	-2.273260	-1.435735	-1.919933
H	-3.384492	-1.789414	-3.254774
C	-2.278018	4.566088	3.037493
H	-1.451240	5.274570	2.870671
H	-2.247793	4.282999	4.101129
H	-3.223064	5.099008	2.852128
C	-0.802373	2.635828	2.382657
H	-0.731339	1.706109	1.801123
H	-0.662578	2.383167	3.446050
H	0.033789	3.281636	2.071773

Anion of 2

133

scf done: -2685.01652304

Al	0.001740	-0.000115	-1.626533
O	-1.320712	0.063368	-0.385164
N	-3.049871	1.879114	-0.311669
O	1.319930	-0.065316	-0.380701
N	-3.675765	-0.197504	0.427571
N	3.051279	-1.879035	-0.307775
N	3.675288	0.198545	0.430200
C	-2.319653	2.985580	-0.796976
C	-2.416300	3.341682	-2.157263
C	-3.670095	-1.517732	0.922021
C	-4.750541	0.686787	0.542419
H	-5.723200	0.361452	0.907583
C	-4.380756	1.914238	0.097906
H	-4.977148	2.822383	0.032084
C	-1.456252	3.679365	0.076501
C	-1.656841	4.419031	-2.621716
H	-1.718309	4.704186	-3.675256
C	-3.751711	-1.741360	2.313558
C	-0.801255	5.111000	-1.771199
H	-0.198428	5.938131	-2.155578
C	-3.313372	2.568195	-3.102174
H	-3.647163	1.673358	-2.555702
C	-0.697602	4.735768	-0.435674
H	-0.004833	5.265789	0.223173
C	-1.316191	3.269190	1.527709
H	-2.135591	2.565462	1.737819
C	2.321933	-2.986186	-0.792744
C	-3.525466	-2.592350	0.018607
C	-3.410345	-2.323160	-1.469092
H	-2.769252	-1.435671	-1.574473
C	-3.716884	-0.565568	3.272984
H	-4.259152	0.265978	2.797584
C	3.668095	1.518989	0.924079
B	-2.533134	0.527078	-0.127693
C	-3.752296	-3.059423	2.779033
H	-3.817179	-3.253241	3.851760
C	-3.509846	-3.892364	0.531731
H	-3.392541	-4.736046	-0.150872
C	-2.564340	2.089594	-4.343485
H	-2.228657	2.932011	-4.970820
H	-3.218813	1.456202	-4.964220
H	-1.680491	1.495727	-4.058102
C	-3.637152	-4.128935	1.897143
H	-3.626991	-5.153652	2.278288
C	3.747674	1.743236	2.315603
B	2.533170	-0.527468	-0.124057
C	-1.473069	4.452333	2.480900
H	-0.648733	5.175961	2.372991
H	-1.462508	4.102863	3.525859
H	-2.419147	4.989189	2.308350
C	1.456044	-3.677836	0.079980
C	4.382368	-1.912593	0.101296
H	4.979679	-2.820150	0.035681
C	2.421766	-3.345172	-2.152049
C	3.523670	2.593059	0.020027
C	0.000532	2.530261	1.765223
H	0.086607	1.643248	1.121790
H	0.068376	2.200212	2.813646
H	0.869395	3.176561	1.562026
C	4.751011	-0.684553	0.545171
H	5.723342	-0.358102	0.910219
C	3.746599	3.061484	2.780549

H	3.809892	3.255799	3.853286
C	3.631750	4.130523	1.898032
H	3.620259	5.155393	2.278731
C	1.313417	-3.265725	1.530380
H	2.130443	-2.559125	1.739995
C	-4.389987	-0.838314	4.612880
H	-3.838121	-1.584101	5.207928
H	-5.420597	-1.204872	4.484596
H	-4.428068	0.084899	5.212178
C	3.506358	3.893293	0.532552
H	3.389154	4.736586	-0.150552
C	3.712308	0.567723	3.275310
H	4.254997	-0.263880	2.800470
C	0.697911	-4.734706	-0.431995
H	0.003226	-5.262971	0.226264
C	-4.777134	-1.985399	-2.066922
H	-5.463441	-2.845483	-1.989226
H	-4.677527	-1.722842	-3.132619
H	-5.238184	-1.130583	-1.550560
C	2.266990	0.100494	3.469722
H	1.680793	0.865247	4.004019
H	2.231205	-0.831415	4.057197
H	1.770167	-0.082597	2.506560
C	3.320885	-2.573745	-3.096718
H	3.656662	-1.679647	-2.550278
C	0.804595	-5.112694	-1.766499
H	0.202164	-5.940183	-2.150731
C	-4.557386	3.379300	-3.465201
H	-5.122524	3.667876	-2.565600
H	-5.228746	2.796189	-4.116500
H	-4.286882	4.304287	-4.001493
C	3.410347	2.323047	-1.467642
H	2.770371	1.434753	-1.573314
C	-2.271744	-0.098246	3.468344
H	-2.236457	0.833794	4.055640
H	-1.774268	0.084727	2.505512
H	-1.685861	-0.862829	4.003218
C	1.662856	-4.423034	-2.616205
H	1.726819	-4.710340	-3.669025
C	-2.723290	-3.438004	-2.246038
H	-1.742809	-3.684155	-1.811621
H	-2.547479	-3.112151	-3.282466
H	-3.330179	-4.358720	-2.286544
C	4.563057	-3.387560	-3.459896
H	5.127488	-3.677724	-2.560349
H	5.235771	-2.805781	-4.110987
H	4.290598	-4.311779	-3.996517
C	4.384445	0.840913	4.615602
H	5.415155	1.207399	4.487921
H	4.422071	-0.082082	5.215264
H	3.832173	1.586932	5.209982
C	4.778109	1.986484	-2.063915
H	4.679886	1.723434	-3.129616
H	5.239511	1.132349	-1.546746
H	5.463430	2.847317	-1.985812
C	2.572725	-2.093467	-4.337959
H	2.234961	-2.935246	-4.965032
H	3.228467	-1.461606	-4.958925
H	1.690178	-1.497679	-4.052523
C	2.722899	3.436819	-2.245745
H	3.328874	4.358148	-2.286018
H	1.741748	3.682125	-1.812401
H	2.548435	3.110332	-3.282196
C	1.473700	-4.447117	2.485228
H	0.652279	-5.174035	2.377152
H	1.460663	-4.096469	3.529773
H	2.422010	-4.980556	2.314343
C	-0.005747	-2.530686	1.766137
H	-0.094115	-1.644854	1.121389
H	-0.075102	-2.199327	2.814045
H	-0.872549	-3.179945	1.563572

Benzene

12

scf done: -232.061998655

C	0.005863	-0.000000	0.003385
C	0.005965	-0.000000	1.398174
C	1.213795	-0.000000	2.095575
C	2.421721	0.000000	1.398181
C	2.421719	0.000000	0.003389
C	1.213836	0.000000	-0.693921
H	-0.940478	-0.000000	1.944643
H	1.213726	-0.000000	3.188409
H	3.368156	0.000000	1.944608
H	3.368105	0.000000	-0.543089
H	1.213869	0.000000	-1.786799
H	-0.940595	-0.000000	-0.543055

Compound 1+Benzene

146

scf done: -3516.81187762

C	-2.132044	-1.676496	-2.849925
C	-2.765727	-2.066978	-1.645023
C	-2.785754	-3.420731	-1.240354
C	-2.193316	-4.381095	-2.072252
C	-1.563516	-4.009502	-3.255956
C	-1.531053	-2.667329	-3.634707
N	-3.280614	-1.074758	-0.787345
C	-4.626781	-0.869458	-0.473821
C	-4.711109	0.006548	0.557159
N	-3.430832	0.397276	0.959898
C	-3.197788	1.117868	2.160836
C	-3.178225	0.408627	3.380158
C	-2.986169	1.123316	4.565029
C	-2.802441	2.501491	4.543442
C	-2.793119	3.183126	3.331998
C	-2.989515	2.511151	2.121074
C	-3.281292	-1.104756	3.394453
C	-4.077021	-1.652214	4.574560
C	-3.013575	3.264431	0.806709
C	-4.441067	3.680931	0.447095
C	-3.415332	-3.838111	0.074816
C	-2.471389	-4.682086	0.929990
C	-2.152962	-0.226214	-3.289603
C	-3.510565	0.101151	-3.915918
B	-2.462528	-0.287954	0.127230
O	-1.117909	-0.304462	0.172833
Al	0.092417	1.089970	0.014182
C	-0.594785	4.137394	-5.320074
C	-1.988954	4.096978	-5.356043
C	-2.706901	3.822086	-4.191402
C	-2.029950	3.580836	-2.995849
C	-0.636094	3.627794	-2.955798
C	0.079625	3.906661	-4.120353
O	1.499508	-0.121174	0.059127
B	2.793410	-0.068692	0.418439
N	3.552982	0.985453	1.058069
C	3.083816	2.283849	1.376572
C	2.242899	2.450589	2.495698

C	1.728188	3.723577	2.751126
C	2.035132	4.796951	1.921541
C	2.862821	4.614533	0.819151
C	3.397052	3.357775	0.522973
C	1.922061	1.272382	3.394285
C	0.545325	1.358770	4.038798
C	4.208734	3.127542	-0.737450
C	5.164500	4.271280	-1.060938
C	4.846394	0.523353	1.296792
C	4.968814	-0.751671	0.851569
N	3.751499	-1.157503	0.292253
C	3.463727	-2.445560	-0.195069
C	3.307709	-2.634198	-1.588535
C	2.966280	-3.910650	-2.052053
C	2.775370	-4.969064	-1.164751
C	2.905981	-4.763245	0.206751
C	3.238845	-3.501649	0.715756
C	3.497802	-1.464054	-2.535728
C	2.782181	-1.635571	-3.870381
C	3.237681	-3.226738	2.207906
C	3.673991	-4.416905	3.053756
C	3.020404	1.075199	4.440481
C	3.272977	2.836825	-1.912944
C	1.854439	-2.722523	2.635041
C	4.983593	-1.164440	-2.743490
C	-1.879417	-1.718919	3.337618
C	-2.059901	4.452738	0.776282
C	-4.742487	-4.560240	-0.156892
C	-1.011750	0.156937	-4.222230
K	0.273994	-2.389240	-0.555007
H	5.840290	-1.402965	0.871206
H	-5.602105	0.405228	1.037969
H	-5.430294	-1.336142	-1.039912
H	5.599243	1.155021	1.764362
H	3.053344	-0.587426	-2.037958
H	3.948852	-2.406506	2.389787
H	-1.096050	-4.767345	-3.889648
H	-2.586056	3.340775	-2.086855
H	-2.049864	0.389833	-2.381862
H	-0.119686	3.410562	-2.015063
H	3.082936	5.462398	0.166958
H	-2.968464	0.593044	5.519525
H	-2.650785	3.047011	5.478196
H	-1.034466	-2.385618	-4.564968
H	2.516632	-5.960004	-1.546100
H	2.844897	-4.082135	-3.123105
H	-3.632303	-2.916547	0.634820
H	4.818191	2.225550	-0.574941
H	-2.213339	-5.434067	-1.779251
H	-2.665883	2.552621	0.039527
H	-0.031670	4.350977	-6.232285
H	1.060618	3.875220	3.601069
H	2.735476	-5.595294	0.893127
H	1.171885	3.935630	-4.092636
H	1.615106	5.783950	2.130057
H	-2.036757	4.891571	-0.232854
H	-1.034464	4.138459	1.025005
H	-2.369086	5.252420	1.468569
H	1.923201	0.378247	2.751986
H	5.486734	-0.955716	-1.788981
H	5.110176	-0.282402	-3.390522
H	5.490751	-2.016493	-3.224103
H	-2.627863	4.261831	3.324166
H	-3.813026	-1.403278	2.477512
H	-2.517695	4.280526	-6.294790
H	2.584315	2.009895	-1.681955
H	3.844778	2.576605	-2.818739
H	2.651204	3.718079	-2.138299
H	-3.799345	3.792127	-4.216883
H	3.238901	-2.424223	-4.489741
H	2.834842	-0.699686	-4.446147
H	1.716446	-1.883089	-3.740703
H	-1.078133	-0.353270	-5.196815
H	-0.030280	-0.072299	-3.778126
H	-1.038578	1.238081	-4.416648
H	-3.544428	1.159689	-4.212683
H	-4.332639	-0.083558	-3.209324
H	-3.683749	-0.513643	-4.814058
H	4.627244	5.193702	-1.332675
H	5.801201	4.005289	-1.918989
H	5.820534	4.502972	-0.208031
H	-4.849621	4.373072	1.201188
H	-5.112229	2.811849	0.385592
H	-4.461997	4.194693	-0.527671
H	3.086815	1.951305	5.105520
H	4.003384	0.932253	3.968006
H	2.811170	0.190781	5.064186
H	0.314258	0.422582	4.569218
H	-0.244478	1.525161	3.290076
H	0.480260	2.171125	4.779558
H	-1.302380	-1.452456	4.237575
H	-1.322893	-1.349504	2.463090
H	-1.933850	-2.818251	3.285847
H	-5.073177	-1.188980	4.638536
H	-3.564027	-1.482965	5.534539
H	-4.212839	-2.740142	4.470419
H	1.089159	-3.504988	2.489412
H	1.556799	-1.827298	2.068172
H	1.844539	-2.449508	3.701514
H	-4.594988	-5.495916	-0.720245
H	-5.440620	-3.932075	-0.728834
H	-5.221377	-4.813966	0.801659
H	-2.217402	-5.640447	0.450362
H	-2.936986	-4.914096	1.900062
H	-1.530693	-4.150499	1.146103
H	3.766235	-4.116738	4.108344
H	4.647894	-4.811476	2.726781
H	2.944439	-5.241887	3.018167

Anion of 2+Benzene

145

scf done: -2917.08551356

C	5.168682	1.215896	-0.415281
C	4.510945	0.100645	0.141902
C	4.499403	-0.124528	1.534825
C	5.192567	0.772604	2.352760
C	5.876467	1.857597	1.812915
C	5.859381	2.079439	0.439588
N	3.823684	-0.792206	-0.706671
C	4.458586	-1.709690	-1.546485
C	3.529395	-2.481325	-2.162756
N	2.255048	-2.085292	-1.762791
C	1.063603	-2.773226	-2.076991
C	0.079809	-2.119683	-2.853395
C	-1.112974	-2.797326	-3.115483
C	-1.327032	-4.089878	-2.643566
C	-0.354878	-4.714912	-1.872896
C	0.840407	-4.062867	-1.554446
C	0.329378	-0.715192	-3.368735
C	1.328033	-0.724595	-4.527816

C	1.825293	-4.711640	-0.601424
C	1.169045	-5.016902	0.744781
C	3.755785	-1.316148	2.107194
C	4.530128	-2.615834	1.879639
C	5.024721	1.503534	-1.897729
C	3.670079	2.165378	-2.161353
B	2.380553	-0.981193	-0.811513
O	1.407839	-0.318355	-0.189965
Al	-0.086817	-0.952041	0.675348
O	-1.240273	0.334976	0.056997
B	-1.853218	1.424986	0.504566
N	-2.709053	2.359189	-0.234482
C	-2.916610	2.452213	-1.628941
C	-2.131028	3.349877	-2.382948
C	-2.389417	3.479380	-3.749983
C	-3.379273	2.720167	-4.365783
C	-4.116325	1.806711	-3.620661
C	-3.898602	1.655288	-2.247964
C	-0.964126	4.064852	-1.732850
C	0.270748	3.160722	-1.765449
C	-4.710958	0.676830	-1.425995
C	-5.098142	-0.581431	-2.194065
C	-3.091260	3.390754	0.628193
C	-2.560385	3.180246	1.856193
N	-1.812922	2.004240	1.848564
C	-0.971903	1.635834	2.924423
C	-1.367807	0.600198	3.798386
C	-0.549569	0.306704	4.890919
C	0.637607	1.000656	5.106856
C	1.045267	1.975329	4.204767
C	0.260307	2.298044	3.092975
C	-2.648205	-0.167836	3.538339
C	-2.604437	-1.602110	4.048974
C	0.765206	3.302047	2.076134
C	0.852184	4.709153	2.664364
C	-0.660854	5.430199	-2.339486
C	-5.935605	1.370501	-0.827292
C	2.095270	2.849613	1.474257
C	-3.866876	0.582638	4.076203
C	-0.942303	0.028494	-3.745007
C	2.475484	-5.951475	-1.212379
C	3.372679	-1.157609	3.571742
C	6.163309	2.329511	-2.483468
C	-3.110874	-3.537236	0.843349
C	-3.657649	-2.262974	0.696254
C	-4.927405	-1.985854	1.201565
C	-5.654929	-2.982831	1.854120
C	-5.110575	-4.259311	1.996754
C	-3.839319	-4.535329	1.489459
H	5.542793	-1.747621	-1.636423
H	-3.745258	4.194902	0.295332
H	-2.680333	3.772610	2.761600
H	3.681059	-3.287055	-2.878669
H	2.808216	-1.388698	1.550352
H	5.015982	0.534563	-2.420854
H	1.264621	0.754526	5.968021
H	-3.063565	-1.482941	0.212549
H	-2.751011	-0.223524	2.443690
H	-2.100777	-3.724288	0.469791
H	-0.542748	-5.715249	-1.472864
H	-1.792690	4.171599	-4.348045
H	-3.564166	2.827651	-5.437927
H	-0.836809	-0.492356	5.576469
H	6.415019	2.545537	2.470369
H	5.193505	0.621521	3.433791

H	0.027134	3.331690	1.261377
H	2.621499	-3.977634	-0.410819
H	2.003071	2.482788	4.349196
H	-4.065061	0.370464	-0.589873
H	-5.676646	-5.040619	2.511583
H	-1.894254	-2.303958	-3.695755
H	6.377947	2.948385	0.029046
H	-3.408228	-5.533011	1.609396
H	-2.269240	-4.600448	-2.858676
H	-5.565631	-1.311657	-1.517108
H	-4.213760	-1.061107	-2.640268
H	-5.819591	-0.369969	-3.000358
H	0.784046	-0.158965	-2.533780
H	4.748870	-2.776693	0.813968
H	3.942366	-3.476917	2.236369
H	5.486619	-2.603047	2.428978
H	-4.873871	1.195714	-4.115811
H	-1.226328	4.228915	-0.675837
H	-6.646238	-2.761389	2.259007
H	0.708352	-4.105509	1.161444
H	1.918078	-5.390129	1.462565
H	0.384982	-5.786843	0.651809
H	-5.344404	-0.980344	1.102441
H	4.250961	-1.173715	4.239589
H	2.711710	-1.985123	3.870744
H	2.817033	-0.224121	3.741485
H	-2.585864	-1.650885	5.150823
H	-1.719811	-2.123020	3.651357
H	-3.496875	-2.148646	3.708896
H	-4.791937	0.034581	3.833660
H	-3.943223	1.589259	3.639266
H	-3.810484	0.687310	5.172714
H	1.725601	-6.728973	-1.434161
H	3.214191	-6.386891	-0.519902
H	2.992537	-5.710407	-2.154077
H	-6.599591	1.747100	-1.622980
H	-5.637191	2.222481	-0.198617
H	-6.517550	0.673047	-0.202762
H	0.915308	-1.267987	-5.394308
H	2.275895	-1.202518	-4.241122
H	1.552534	0.305752	-4.849050
H	-0.709574	1.069584	-4.011189
H	-1.646974	0.060911	-2.902309
H	-1.451496	-0.420366	-4.614054
H	0.581313	2.966233	-2.805175
H	0.084702	2.188656	-1.284577
H	1.115073	3.637408	-1.245817
H	-1.553526	6.074966	-2.360247
H	-0.277054	5.349520	-3.369625
H	0.115577	5.940413	-1.748312
H	3.632243	3.164109	-1.697258
H	2.846421	1.572586	-1.739070
H	3.490595	2.283020	-3.242379
H	1.576116	4.754030	3.494930
H	-0.124275	5.041115	3.049681
H	1.179079	5.430456	1.897731
H	2.890001	2.777465	2.233262
H	2.438356	3.566768	0.711703
H	1.999471	1.863767	0.994343
H	6.051857	2.406699	-3.576326
H	7.145836	1.879389	-2.271416
H	6.170920	3.358349	-2.088108

Compound 1: TS, OA

146

scf done: -3516.75635605

C	1.923022	-1.367121	3.179637
C	0.552431	-1.512398	2.589267
C	-0.459111	-1.767319	3.655450
C	-0.083348	-2.609002	4.682989
C	1.264150	-2.982579	4.883433
C	2.245597	-2.206493	4.221455
Al	0.236273	-0.637462	0.950277
O	-1.142499	0.214270	0.296139
B	-2.442338	0.398631	0.643909
N	-2.962311	1.558454	1.334920
C	-2.262472	2.764476	1.561246
C	-1.334390	2.845987	2.625160
C	-0.616877	4.039522	2.786020
C	-0.809070	5.120224	1.927106
C	-1.715129	5.019838	0.873092
C	-2.443906	3.841735	0.662982
C	-1.133288	1.679698	3.568946
C	-2.247960	1.628399	4.614924
C	-3.319859	3.681394	-0.566612
C	-2.491535	3.105277	-1.719672
O	1.510617	0.018267	-0.041809
B	2.549030	-0.327238	-0.835844
N	2.860440	-1.593231	-1.437393
C	2.063304	-2.769939	-1.400622
C	1.003684	-2.894178	-2.320940
C	0.225989	-4.056316	-2.290525
C	0.496654	-5.064935	-1.376035
C	1.524574	-4.912497	-0.450314
C	2.318199	-3.762450	-0.428192
C	0.684821	-1.804173	-3.323949
C	-0.705108	-1.217921	-3.080727
C	3.418750	-3.590209	0.602502
C	4.797620	-3.862627	-0.005110
C	3.945359	-1.403985	-2.296024
C	4.351367	-0.111137	-2.251094
N	3.532789	0.593934	-1.358654
C	3.538098	1.984506	-1.133014
C	3.125236	2.864570	-2.158874
C	3.063089	4.236161	-1.877710
C	3.374979	4.721326	-0.610196
C	3.764623	3.840114	0.397997
C	3.860532	2.463668	0.160369
C	2.648664	2.325055	-3.495813
C	2.957122	3.244150	-4.672228
C	4.313493	1.500948	1.242067
C	4.165928	2.047920	2.656006
C	0.842133	-2.298207	-4.761200
C	3.217108	-4.438919	1.850985
C	5.748588	1.037153	0.980544
C	1.152874	2.004648	-3.425721
N	-3.594908	-0.420708	0.368161
C	-4.713298	0.222726	0.904355
C	-4.345934	1.397130	1.470892
C	-3.676365	-1.547289	-0.494260
C	-4.236900	-1.388808	-1.777643
C	-4.307976	-2.507378	-2.614840
C	-3.809703	-3.737936	-2.207046
C	-3.238877	-3.872201	-0.944953
C	-3.168276	-2.791274	-0.062584
C	-4.765555	-0.052821	-2.266311
C	-6.293678	-0.050672	-2.322233
C	-2.615347	-2.962490	1.337459
C	-3.745815	-2.993330	2.368361
C	-4.170226	0.347252	-3.615026
C	-1.708436	-4.172215	1.511127
C	0.239460	1.643862	4.225798
C	-4.025606	4.964605	-0.989399
K	0.502025	2.557171	-0.107331
H	5.196519	0.357310	-2.751096
H	-5.699720	-0.234153	0.868981
H	-4.958179	2.120330	2.005500
H	4.362555	-2.225958	-2.873554
H	3.665508	0.613446	1.173208
H	3.168074	1.371828	-3.673539
H	-0.249005	6.046161	2.080410
H	-1.467149	-1.341276	3.623091
H	-1.223141	0.757586	2.974649
H	1.697948	-5.694428	0.289436
H	-4.749839	-2.406896	-3.609349
H	-3.864562	-4.600306	-2.876249
H	0.100134	4.121840	3.604674
H	3.319225	5.793473	-0.406118
H	4.006949	4.232142	1.387043
H	-4.097053	2.940303	-0.325648
H	3.390139	-2.534990	0.925290
H	-1.851585	5.867649	0.198398
H	-2.008032	-2.071216	1.572261
H	3.272864	-2.204770	4.609464
H	-0.607400	-4.165773	-2.988510
H	2.756151	4.934103	-2.659554
H	2.647599	-0.639358	2.798839
H	-0.114933	-5.970412	-1.364155
H	-1.207853	-4.104649	2.488917
H	-0.929867	-4.215066	0.737107
H	-2.278223	-5.115581	1.482523
H	1.420306	-0.999236	-3.177661
H	5.846619	0.564711	-0.006976
H	6.053046	0.299114	1.738128
H	6.448329	1.886815	1.029397
H	-2.843790	-4.839660	-0.633361
H	-4.459712	0.708908	-1.533704
H	1.541186	-3.637373	5.712057
H	2.233889	-4.271904	2.314654
H	3.967998	-4.167428	2.606412
H	3.340015	-5.513597	1.637253
H	-0.836017	-2.917113	5.420747
H	4.874681	2.866391	2.861268
H	4.361823	1.250021	3.386583
H	3.147914	2.418903	2.850917
H	0.361052	2.434372	4.984457
H	1.050792	1.747370	3.487412
H	0.388077	0.664080	4.703426
H	-2.112824	0.739942	5.249728
H	-3.240555	1.565530	4.145288
H	-2.224657	2.523527	5.257361
H	4.860111	-4.900519	-0.370567
H	5.581862	-3.725946	0.755787
H	5.025954	-3.190767	-0.843367
H	-4.405239	-3.857583	2.188272
H	-4.358584	-2.081159	2.335707
H	-3.321828	-3.085221	3.379546
H	0.124581	-3.101321	-4.992467
H	1.854352	-2.691900	-4.938023
H	0.664383	-1.478554	-5.475785
H	-0.913662	-0.411125	-3.801512
H	-0.802003	-0.802780	-2.066109
H	-1.496209	-1.975382	-3.192924

H	-4.456617	-0.353505	-4.414644
H	-3.072121	0.375263	-3.574300
H	-4.531056	1.344410	-3.913957
H	-6.732773	-0.293335	-1.343741
H	-6.663644	-0.795339	-3.045115
H	-6.674083	0.936176	-2.630715
H	0.560400	2.918380	-3.243330
H	0.932684	1.266262	-2.639422
H	0.796744	1.570597	-4.372301
H	-3.322047	5.723342	-1.367838
H	-4.586069	5.409987	-0.154006
H	-4.737458	4.753896	-1.801653
H	-1.677135	3.796814	-2.000212
H	-3.110838	2.954310	-2.615937
H	-2.061828	2.123834	-1.462566
H	2.704829	2.743598	-5.619085
H	4.023274	3.513942	-4.704396
H	2.372858	4.177527	-4.637130
H	0.584928	-2.485427	1.890032

Compound 1: TS, C-C insertion

146

scf done: -3516.77140668

C	2.054907	3.539645	-0.416179
C	2.732041	2.887237	0.644040
C	2.743934	3.431738	1.951798
C	2.083926	4.647517	2.174990
C	1.414713	5.299037	1.142087
C	1.401974	4.746236	-0.136929
N	3.351084	1.648499	0.398428
B	2.684832	0.387286	0.091088
O	1.349546	0.238978	-0.005592
Al	-0.068663	-0.344761	-0.890819
C	-0.325824	0.008704	-2.755854
C	0.656719	-0.047204	-3.784969
C	1.653688	-0.988187	-3.914822
C	1.601143	-2.281352	-3.313042
C	0.536532	-2.728669	-2.565468
C	-0.436230	-1.923130	-1.904709
C	3.383081	2.669824	3.102183
C	2.456028	1.554671	3.593116
C	2.075410	2.960138	-1.818761
C	3.445827	3.175499	-2.465268
C	4.717904	1.399451	0.566579
C	4.953195	0.081549	0.375470
N	3.764720	-0.584509	0.075242
C	3.693540	-1.974086	-0.196709
C	4.433913	-2.531887	-1.262701
C	4.302402	-3.899916	-1.513461
C	3.452122	-4.697496	-0.758064
C	2.744358	-4.137774	0.298153
C	2.866459	-2.782125	0.611032
C	5.400127	-1.715005	-2.103211
C	6.845991	-2.029280	-1.707978
C	2.183986	-2.240629	1.851990
C	2.948576	-2.663002	3.107109
O	-1.388901	0.152838	0.220987
B	-2.698584	-0.193823	0.215380
N	-3.755961	0.288263	-0.656008
C	-3.638709	1.196929	-1.732720
C	-3.821101	0.753769	-3.057036
C	-3.662868	1.682886	-4.091400
C	-3.310981	3.000267	-3.828831
C	-3.132834	3.424149	-2.514116
C	-3.304776	2.539154	-1.448789
C	-4.188801	-0.683593	-3.373019
C	-3.301869	-1.306343	-4.450151
C	-3.214813	3.008499	-0.008395
C	-4.608389	3.296735	0.553048
N	-3.388284	-1.063557	1.147672
C	-2.861133	-1.812351	2.224040
C	-2.479655	-1.139563	3.404110
C	-2.012746	-1.893742	4.482358
C	-1.924684	-3.281135	4.396645
C	-2.279347	-3.926949	3.219235
C	-2.739547	-3.209751	2.109577
C	-2.624978	0.367077	3.497381
C	-4.034917	0.760298	3.939642
C	-3.096340	-3.932371	0.825218
C	-4.343466	-4.797514	1.008589
C	-4.748100	-1.077396	0.823799
C	-4.964953	-0.275405	-0.244219
C	-5.665828	-0.785332	-3.761237
C	-2.284897	4.198884	0.195189
C	0.713872	-2.625641	1.933766
C	5.212058	-1.907268	-3.605649
C	-1.565350	1.020374	4.380254
C	-1.926043	-4.750466	0.280732
C	3.820201	3.555938	4.262119
C	0.958011	3.475698	-2.715603
H	-5.462953	-1.664976	1.395479
H	-5.901779	-0.048471	-0.747169
H	-3.795851	1.357413	-5.125671
H	-3.173429	3.704737	-4.652488
H	-4.040986	-1.264855	-2.450030
H	-2.865781	4.464962	-2.320885
H	-2.812890	2.161778	0.569143
H	-3.331316	-3.161987	0.075800
H	-2.501124	0.749166	2.471623
H	-1.718495	-1.396250	5.408956
H	-2.185940	-5.013862	3.152028
H	-3.424955	-0.804496	-5.423175
H	-3.574793	-2.363317	-4.594191
H	-2.239161	-1.265563	-4.172674
H	-1.563565	-3.858645	5.250881
H	-5.090510	4.113845	-0.006516
H	-4.552897	3.593254	1.613286
H	-5.251844	2.408147	0.484257
H	5.902364	-0.444106	0.428097
H	-1.293648	4.034094	-0.255411
H	-2.154501	4.412221	1.269947
H	-2.688422	5.120188	-0.252309
H	5.430789	2.196603	0.765698
H	2.090160	5.090671	3.172532
H	0.905277	6.246890	1.332509
H	2.228232	-1.146148	1.801266
H	-4.249388	0.369893	4.947083
H	-4.787942	0.356647	3.247896
H	-4.150548	1.856129	3.964167
H	0.881738	5.269361	-0.940615
H	4.281703	2.172047	2.709512
H	2.092542	-4.769575	0.906199
H	-4.170747	-5.596959	1.747322
H	-4.627152	-5.275818	0.058146
H	-5.200098	-4.201475	1.357364
H	1.531017	1.974508	4.025915
H	2.938715	0.959628	4.382978
H	2.201669	0.853292	2.783293
H	5.209556	-0.652245	-1.893192
H	3.347031	-5.760331	-0.988562

H	-6.325351	-0.375092	-2.982165
H	-5.951993	-1.835094	-3.931515
H	-5.866133	-0.227277	-4.690248
H	1.921879	1.874434	-1.729866
H	-1.603743	2.119761	4.290211
H	-0.550306	0.670544	4.129815
H	-1.720933	0.796140	5.446558
H	4.867203	-4.346604	-2.335224
H	-1.049206	-4.117042	0.087156
H	-2.208126	-5.229711	-0.669595
H	-1.626086	-5.550049	0.977195
H	7.020994	-1.901022	-0.629090
H	7.551885	-1.379797	-2.249765
H	7.096013	-3.074519	-1.951096
H	4.461113	4.383075	3.921890
H	4.391023	2.963915	4.993036
H	2.963003	3.990774	4.801083
H	3.477198	2.684436	-3.449718
H	4.252734	2.749417	-1.851955
H	3.649566	4.249231	-2.607697
H	5.402097	-2.946388	-3.917136
H	5.917604	-1.267298	-4.158545
H	4.194128	-1.637558	-3.910507
H	1.064498	4.548251	-2.947006
H	-0.037471	3.306477	-2.276197
H	0.976374	2.927866	-3.667578
H	2.957632	-3.759456	3.215497
H	2.479787	-2.239586	4.010751
H	3.994622	-2.322334	3.069198
H	0.182556	-2.371068	1.004907
H	0.213179	-2.108179	2.765657
H	0.578641	-3.704634	2.102834
H	0.381461	-3.815955	-2.539766
H	-1.443414	-2.376093	-1.858504
H	2.313622	-3.027900	-3.675103
H	2.376064	-0.850667	-4.726601
H	0.554126	0.669719	-4.612106
H	-1.282983	0.446173	-3.078769
K	-0.118568	1.991676	1.508923

Compound 1: TS, [4+1] cycloaddition

146

scf done: -3516.77829162

C	-1.631283	-3.837217	0.418014
C	-1.818215	-3.303604	-0.879797
C	-1.140712	-3.842757	-1.997611
C	-0.300858	-4.946179	-1.800813
C	-0.117486	-5.488351	-0.530919
C	-0.772449	-4.932956	0.567080
N	-2.648220	-2.181557	-1.045129
C	-3.868115	-2.222517	-1.727970
C	-4.426017	-0.991701	-1.706760
N	-3.601433	-0.098593	-1.021652
C	-4.019509	1.220457	-0.719293
C	-3.277684	2.308440	-1.227994
C	-3.687000	3.600885	-0.893627
C	-4.819159	3.818912	-0.114300
C	-5.555474	2.738356	0.354439
C	-5.163297	1.423554	0.081491
C	-2.115936	2.071270	-2.175907
C	-2.628919	1.687591	-3.566259
C	-5.970367	0.271881	0.657090
C	-6.158932	0.375980	2.170603
C	-1.249639	-3.160225	-3.349392
C	-0.356715	-1.916344	-3.377606
C	-2.340033	-3.209850	1.604642
C	-3.826554	-3.572888	1.615380
B	-2.401652	-0.808781	-0.593249
O	-1.274645	-0.389422	0.002631
Al	-0.419370	1.018344	0.860371
O	1.270155	0.401052	0.402785
B	2.549963	0.805325	0.446874
N	3.176762	2.038772	-0.000129
C	2.652607	3.090653	-0.800045
C	2.768200	2.985875	-2.202252
C	2.344147	4.058162	-2.990866
C	1.802507	5.197063	-2.407310
C	1.660557	5.270457	-1.026501
C	2.079611	4.226339	-0.195147
C	3.261439	1.701178	-2.839737
C	2.079151	0.754176	-3.063488
C	1.938553	4.316466	1.312821
C	0.864499	5.303442	1.750101
C	-1.771599	2.852942	2.636034
C	-0.696662	2.701955	3.556874
C	-0.297484	1.428196	3.869707
C	-0.981825	0.340389	3.238292
C	-2.363315	0.547898	2.916821
C	-2.751365	1.824243	2.589259
N	3.679726	-0.015462	0.893599
C	3.648383	-1.396989	1.174729
C	4.064033	-2.319746	0.185916
C	3.978993	-3.690651	0.463682
C	3.467922	-4.143049	1.674272
C	3.045735	-3.226103	2.635655
C	3.132442	-1.846688	2.415317
C	4.579829	-1.855462	-1.163409
C	6.099442	-1.999560	-1.251440
C	2.695927	-0.847680	3.471241
C	1.879139	-1.472814	4.595275
C	4.860193	0.708058	0.697543
C	4.558103	1.918381	0.175005
C	4.048274	1.912250	-4.128345
C	3.274244	4.648293	1.981828
C	-1.139453	3.234479	-2.253426
C	-7.327767	0.151313	-0.038424
C	3.904291	-2.574785	-2.330074
C	3.895938	-0.095744	4.053777
C	-0.946456	-4.074156	-4.529699
C	-1.690730	-3.530243	2.945324
H	-4.257376	-3.142248	-2.159600
H	5.230847	2.735510	-0.077626
H	5.830577	0.317023	0.995547
H	-5.369337	-0.670118	-2.140973
H	-2.266910	-2.119637	1.465614
H	-2.286784	-2.807872	-3.457165
H	3.396817	-5.214949	1.874076
H	-2.038391	3.848172	2.274337
H	2.055578	-0.109945	2.958557
H	-3.742617	2.043166	2.191640
H	-6.441848	2.916296	0.968665
H	2.429456	3.998362	-4.077734
H	1.472812	6.029100	-3.034247
H	2.647599	-3.595965	3.581219
H	0.544692	-6.346622	-0.393780
H	-0.611476	-5.361677	1.557516
H	4.340880	-0.785187	-1.249391
H	-5.409094	-0.654396	0.464552
H	4.304876	-4.413598	-0.288198
H	1.620793	3.317122	1.658282

H	-0.646521	-0.678989	3.449258
H	-3.115159	4.455127	-1.258159
H	0.227747	-5.381695	-2.650704
H	-3.038867	-0.303445	2.796934
H	-5.128017	4.838734	0.127787
H	0.740014	5.264925	2.841784
H	-0.105744	5.065776	1.289519
H	1.130844	6.342069	1.496834
H	-1.552667	1.210215	-1.783974
H	-4.319715	-3.279459	0.677923
H	-4.339988	-3.057377	2.442164
H	-3.963025	-4.657492	1.753198
H	1.211625	6.161198	-0.585886
H	3.943519	1.220779	-2.120858
H	0.585355	1.246185	4.487687
H	-5.199143	0.437219	2.701871
H	-6.698044	-0.508288	2.545863
H	-6.750816	1.261481	2.450972
H	-0.148846	3.574092	3.918414
H	-1.806818	-4.590206	3.222994
H	-2.160362	-2.931962	3.740017
H	-0.613717	-3.297026	2.948929
H	2.488514	-2.151824	5.213184
H	1.014607	-2.040425	4.219880
H	1.492966	-0.688987	5.262248
H	3.554967	0.648715	4.789850
H	4.461481	0.436727	3.278050
H	4.578275	-0.792124	4.567281
H	-7.942894	1.047322	0.143214
H	-7.885458	-0.720816	0.338195
H	-7.222396	0.043382	-1.127915
H	3.642998	5.631830	1.647814
H	4.044375	3.898853	1.754129
H	3.156321	4.685373	3.076494
H	-3.204402	2.515320	-4.011124
H	-3.279222	0.801310	-3.529497
H	-1.785046	1.462757	-4.238803
H	-0.264837	2.957012	-2.858665
H	-0.769454	3.511062	-1.254068
H	-1.586544	4.125799	-2.722483
H	1.365480	1.185606	-3.783920
H	1.532990	0.574249	-2.125399
H	2.420090	-0.212189	-3.467567
H	4.875628	2.623436	-3.984490
H	3.413731	2.292406	-4.944499
H	4.475776	0.957530	-4.472939
H	0.706570	-2.197542	-3.292858
H	-0.606537	-1.216189	-2.566482
H	-0.472401	-1.367460	-4.324428
H	6.401941	-3.056246	-1.171453
H	6.601055	-1.445442	-0.445936
H	6.472034	-1.611770	-2.212316
H	4.141943	-3.649749	-2.351553
H	4.238794	-2.146741	-3.287254
H	2.807655	-2.477532	-2.299918
H	-1.163016	-3.552808	-5.474010
H	-1.553833	-4.991521	-4.502398
H	0.114653	-4.369668	-4.562893
K	0.857362	-2.092002	-0.078066

Compound 1: product, OA

146

scf done: -3516.86631843

C	1.780999	2.810413	-2.631754
C	2.541204	2.665447	-1.448542
C	2.596644	3.702823	-0.488995
C	1.858266	4.870286	-0.721921
C	1.074509	5.007538	-1.866377
C	1.041955	3.986071	-2.812752
N	3.175783	1.433876	-1.194508
C	4.508419	1.123293	-1.489976
C	4.715176	-0.195214	-1.256556
N	3.534890	-0.792530	-0.811693
C	3.398687	-2.176000	-0.528055
C	2.654936	-2.982747	-1.417698
C	2.526298	-4.341067	-1.126218
C	3.109187	-4.889209	0.012078
C	3.813385	-4.077672	0.891589
C	3.960139	-2.707906	0.648759
C	2.013411	-2.379469	-2.653867
C	3.045431	-2.165170	-3.763029
C	4.703711	-1.835276	1.640595
C	4.271451	-2.096079	3.081874
C	3.457674	3.563630	0.750049
C	4.798463	4.272554	0.546887
C	1.742113	1.712297	-3.675382
C	0.382101	1.016948	-3.696697
B	2.500319	0.217823	-0.763282
O	1.204451	0.123097	-0.418507
Al	0.230689	-1.007501	0.604927
O	-1.361047	-0.182540	0.394529
B	-2.688763	-0.358981	0.433808
N	-3.515065	-1.500147	0.088803
C	-3.116977	-2.730685	-0.495437
C	-3.248281	-2.895753	-1.888890
C	-2.861181	-4.113882	-2.453263
C	-2.337950	-5.130104	-1.661680
C	-2.191371	-4.942357	-0.291828
C	-2.579148	-3.746406	0.317845
C	-3.706075	-1.737997	-2.755555
C	-2.511969	-0.833830	-3.067904
C	-2.444907	-3.540596	1.814282
C	-1.388130	-4.431030	2.455873
C	-4.855434	-1.129281	0.222428
C	-4.943069	0.159294	0.631097
N	-3.653184	0.674481	0.789700
C	-3.339518	2.020925	1.052660
C	-2.743256	2.360023	2.292951
C	-2.369051	3.691467	2.505696
C	-2.581126	4.667345	1.530431
C	-3.159883	4.319039	0.314683
C	-3.532748	2.993866	0.048205
C	-2.532255	1.295406	3.352994
C	-1.504276	1.675395	4.408992
C	-4.082802	2.624255	-1.316219
C	-5.424276	3.304445	-1.583185
C	-4.417874	-2.165117	-4.033745
C	-3.796158	-3.705452	2.513708
C	-3.071017	2.923112	-2.422803
C	-3.863658	0.910077	4.002714
C	0.813022	-3.167271	-3.163412
C	6.217926	-1.995073	1.491503
C	2.768197	4.052984	2.020847
C	2.133540	2.228572	-5.058480
K	-0.305888	2.264622	0.099848
C	0.865194	-0.585206	2.456327
C	0.453932	-1.299720	3.596872
C	0.919969	-0.995482	4.876973
C	1.819344	0.054827	5.062429
C	2.248372	0.787603	3.957320

C	1.778070	0.462514	2.682626
H	5.207167	1.870440	-1.859204
H	-5.659450	-1.837743	0.032401
H	-5.830509	0.745583	0.860663
H	5.629014	-0.770242	-1.386518
H	3.667752	2.489174	0.867582
H	2.490110	0.962643	-3.378928
H	-2.290598	5.703210	1.722836
H	-0.243909	-2.135138	3.485813
H	-2.143371	0.406407	2.831143
H	4.245326	-4.510475	1.796756
H	-2.953598	-4.265540	-3.530249
H	-2.029853	-6.073851	-2.118388
H	-1.906384	3.973731	3.452680
H	0.495140	5.920453	-2.025589
H	1.891354	5.683485	0.007046
H	-4.255368	1.538340	-1.316390
H	4.447254	-0.791059	1.402766
H	-3.311966	5.085020	-0.450521
H	-2.128689	-2.493631	1.960375
H	2.963304	1.604998	4.087734
H	1.945706	-4.982063	-1.790880
H	0.434888	4.105350	-3.713991
H	2.162506	1.030861	1.827771
H	2.996765	-5.955672	0.222702
H	-1.265752	-4.167722	3.517731
H	-0.412969	-4.318391	1.958816
H	-1.675725	-5.494024	2.425215
H	1.633836	-1.389898	-2.357261
H	5.326190	3.882594	-0.335181
H	5.450718	4.129795	1.422413
H	4.654424	5.355482	0.401791
H	-1.760122	-5.741118	0.312711
H	-4.422007	-1.144851	-2.164814
H	2.189335	0.295172	6.062500
H	3.180806	-2.022264	3.193314
H	4.728876	-1.356190	3.756417
H	4.587117	-3.093028	3.428718
H	0.583228	-1.582576	5.736305
H	2.573200	5.136614	1.997568
H	3.404539	3.858675	2.897173
H	1.810322	3.538693	2.198123
H	-1.840306	2.520934	5.031096
H	-0.529567	1.929059	3.964889
H	-1.330532	0.823018	5.079843
H	-3.709187	0.101896	4.734270
H	-4.591779	0.555548	3.260099
H	-4.301106	1.770497	4.534443
H	6.524458	-3.031045	1.709484
H	6.751612	-1.331571	2.190586
H	6.556978	-1.756759	0.473021
H	-4.175417	-4.732041	2.385686
H	-4.547420	-3.010972	2.112132
H	-3.700154	-3.510933	3.593933
H	3.464414	-3.128417	-4.095968
H	3.877878	-1.530494	-3.426072
H	2.581363	-1.679080	-4.636970
H	0.323391	-2.614730	-3.980492
H	0.069228	-3.328988	-2.369181
H	1.103080	-4.148697	-3.572112
H	-1.767260	-1.373877	-3.672765
H	-2.008823	-0.505527	-2.147087
H	-2.826448	0.058823	-3.631688
H	-5.251718	-2.852305	-3.824404
H	-3.735476	-2.666332	-4.738433
H	-4.824893	-1.284070	-4.553647
H	-0.420743	1.710113	-3.997058
H	0.138155	0.601058	-2.708646
H	0.383117	0.182388	-4.414405
H	-5.325762	4.401704	-1.609369
H	-6.157531	3.053532	-0.802369
H	-5.836470	2.982683	-2.552097
H	-2.809873	3.992621	-2.468204
H	-3.476476	2.638410	-3.405939
H	-2.141224	2.349425	-2.285824
H	2.182605	1.394097	-5.774843
H	3.118672	2.718313	-5.039435
H	1.402609	2.954649	-5.449395
H	0.176883	-2.531300	0.153290

Compound 1: product, C-C insertion

146

scf done: -3516.81155654

C	2.937897	3.047290	1.280550
C	3.155952	1.785636	1.881704
C	3.038691	1.613160	3.278313
C	2.764595	2.733663	4.072067
C	2.595525	3.986993	3.497950
C	2.670253	4.134571	2.115088
N	3.491551	0.680450	1.068256
B	2.656629	-0.045754	0.120799
O	1.340306	0.124406	-0.078319
Al	-0.010676	0.163942	-1.285657
C	-0.029645	1.903037	-2.170342
C	-0.004543	2.166955	-3.502175
C	-0.010521	1.281099	-4.652759
C	-0.018903	-0.072765	-4.825803
C	0.019535	-1.218315	-3.934541
C	0.043821	-1.295151	-2.579090
C	3.205254	0.246907	3.920599
C	2.108963	-0.063739	4.943115
C	3.002464	3.199137	-0.228612
C	4.446064	3.153961	-0.735608
C	4.774618	0.119340	1.046156
C	4.801675	-0.908238	0.165645
N	3.547474	-1.050193	-0.424180
C	3.191787	-2.132651	-1.262695
C	3.547651	-2.100616	-2.625596
C	3.146055	-3.166545	-3.432628
C	2.418547	-4.231940	-2.907987
C	2.090302	-4.255512	-1.557913
C	2.473087	-3.209515	-0.712369
C	4.314026	-0.914075	-3.180284
C	5.118011	-1.243163	-4.432684
C	2.136518	-3.256292	0.765052
C	2.932411	-4.346951	1.480970
O	-1.339371	-0.111117	-0.070559
B	-2.666817	0.010606	0.112291
N	-3.529793	1.140752	-0.176503
C	-3.157329	2.389719	-0.728168
C	-3.528263	2.702276	-2.052321
C	-3.124629	3.932800	-2.572851
C	-2.378581	4.828343	-1.810652
C	-2.031036	4.509596	-0.504047
C	-2.415735	3.289059	0.059584
C	-4.306230	1.697404	-2.882604
C	-3.389624	0.596818	-3.418885
C	-2.054184	2.963142	1.494220
C	-2.812092	3.854170	2.477040
N	-3.554977	-0.938954	0.778093

C	-3.247148	-2.214320	1.303351
C	-3.323349	-2.437354	2.699213
C	-2.999796	-3.708540	3.186951
C	-2.625666	-4.733591	2.325776
C	-2.574749	-4.504627	0.955684
C	-2.878834	-3.251690	0.416027
C	-3.747590	-1.328381	3.653550
C	-4.237746	-1.830225	5.006718
C	-2.858739	-3.016450	-1.084118
C	-4.277652	-2.862595	-1.638926
C	-4.835609	-0.376535	0.839793
C	-4.815382	0.854684	0.278019
C	-5.102273	2.332294	-4.016932
C	-0.548149	3.023854	1.729845
C	0.637297	-3.412067	1.002398
C	3.386547	0.276202	-3.430565
C	-2.646808	-0.284171	3.842085
C	-2.105898	-4.085036	-1.866961
C	4.578784	0.089963	4.575701
C	2.293604	4.442338	-0.752000
H	-5.684919	-0.927480	1.237977
H	-5.633705	1.561777	0.157985
H	-3.387884	4.196646	-3.598251
H	-2.067902	5.783079	-2.241859
H	-5.029742	1.214328	-2.206174
H	-1.453403	5.222039	0.091156
H	-2.389614	1.931059	1.671935
H	-2.325134	-2.067430	-1.251953
H	-4.587390	-0.796058	3.181250
H	-3.052645	-3.906872	4.258420
H	-2.294982	-5.321092	0.288721
H	-2.638713	1.005052	-4.109647
H	-3.976523	-0.164629	-3.956178
H	-2.843089	0.090053	-2.612407
H	-2.382886	-5.721287	2.725180
H	-2.540016	4.913854	2.346559
H	-2.581363	3.574930	3.518141
H	-3.899045	3.766698	2.331742
H	5.633173	-1.559013	-0.097521
H	0.007393	2.419520	0.996675
H	-0.281320	2.689270	2.747737
H	-0.165661	4.051465	1.646592
H	5.588899	0.534811	1.634750
H	2.684288	2.621877	5.156318
H	2.389747	4.854917	4.129032
H	2.457112	-2.292299	1.191517
H	-3.423988	-2.269114	5.606181
H	-5.024569	-2.591140	4.897393
H	-4.653823	-0.994496	5.589334
H	2.510388	5.120250	1.675370
H	3.146266	-0.498386	3.109107
H	1.529625	-5.102447	-1.153169
H	-4.852287	-3.791920	-1.494827
H	-4.240114	-2.648808	-2.718170
H	-4.824046	-2.041891	-1.154455
H	2.226000	0.534740	5.859104
H	2.143946	-1.122955	5.242270
H	1.096533	0.155928	4.567983
H	5.030944	-0.607209	-2.401323
H	2.107856	-5.052267	-3.559502
H	-5.748273	3.149186	-3.659939
H	-5.742653	1.576106	-4.495846
H	-4.443176	2.736988	-4.801366
H	2.470793	2.331872	-0.652338
H	-2.973296	0.515996	4.523718
H	-2.411254	0.191349	2.878641
H	-1.742992	-0.743499	4.280688
H	3.397288	-3.163813	-4.494423
H	-1.086236	-4.239266	-1.484475
H	-2.015740	-3.772960	-2.917463
H	-2.631615	-5.053844	-1.852520
H	5.771715	-2.116581	-4.284403
H	5.751235	-0.386548	-4.709395
H	4.464322	-1.449365	-5.295067
H	5.388403	0.233033	3.848251
H	4.692359	-0.913642	5.014947
H	4.712033	0.831345	5.379680
H	4.463102	3.211571	-1.834957
H	4.955893	2.226123	-0.441324
H	5.023532	4.005269	-0.340126
H	2.643727	0.047211	-4.207823
H	3.966592	1.154695	-3.754553
H	2.828042	0.556498	-2.527831
H	2.824909	5.368444	-0.477931
H	1.260202	4.513991	-0.381853
H	2.245264	4.402240	-1.849960
H	2.680354	-5.345388	1.089346
H	2.717119	-4.346688	2.561980
H	4.013935	-4.197188	1.347403
H	0.052104	-2.657375	0.455359
H	0.386807	-3.356831	2.076670
H	0.272103	-4.392343	0.664015
H	0.059393	-2.163806	-4.497342
H	0.117804	-2.332561	-2.223206
H	-0.036549	-0.370581	-5.881262
H	-0.021936	1.834634	-5.599600
H	-0.003202	3.224038	-3.809691
H	-0.047011	2.821262	-1.565954
K	0.077398	-0.302180	2.098848

Compound 1: product, [4+1] cycloaddition

146

scf done: -3516.82317995

C	1.766515	-3.701310	-0.471927
C	2.052054	-3.174482	0.811274
C	1.496411	-3.747464	1.978784
C	0.671279	-4.871399	1.844393
C	0.383100	-5.400449	0.588738
C	0.922558	-4.815769	-0.555777
N	2.850194	-2.021554	0.914890
C	4.113421	-1.995075	1.514196
C	4.595487	-0.733054	1.466025
N	3.676227	0.115019	0.845622
C	3.992934	1.465922	0.557583
C	3.199931	2.492732	1.112854
C	3.524704	3.816514	0.810265
C	4.620051	4.125029	0.009424
C	5.397330	3.103451	-0.519826
C	5.090429	1.760729	-0.277505
C	2.059325	2.161787	2.057331
C	2.600898	1.773027	3.435462
C	5.918562	0.679704	-0.949420
C	5.985804	0.866260	-2.465291
C	1.708312	-3.082922	3.327587
C	0.764778	-1.885968	3.470900
C	2.365592	-3.061931	-1.711950
C	3.860211	-3.373157	-1.820868
B	2.502894	-0.667359	0.487486
O	1.306025	-0.319776	-0.015539
Al	0.397881	0.714153	-1.171188

O	-1.239340	0.431702	-0.490946
B	-2.551614	0.711281	-0.459388
N	-3.255953	1.886722	0.029473
C	-2.782419	2.963679	0.829011
C	-2.752074	2.793313	2.229492
C	-2.372581	3.875279	3.027033
C	-2.014364	5.089543	2.452836
C	-2.029007	5.236589	1.070721
C	-2.418391	4.185998	0.232766
C	-3.062768	1.442941	2.846018
C	-1.783331	0.609727	2.957066
C	-2.491019	4.364903	-1.270287
C	-1.541182	5.434675	-1.795801
C	1.032070	2.509540	-1.883325
C	0.054672	2.555242	-3.024361
C	-0.096840	1.382798	-3.679170
C	0.743961	0.263085	-3.140675
C	2.168689	0.727342	-3.020561
C	2.319839	1.904009	-2.369224
N	-3.628544	-0.223074	-0.801117
C	-3.505059	-1.601223	-1.083189
C	-3.812646	-2.550989	-0.078272
C	-3.623840	-3.911519	-0.355869
C	-3.118954	-4.325539	-1.582620
C	-2.820227	-3.382710	-2.564019
C	-3.017561	-2.014096	-2.347806
C	-4.335376	-2.129688	1.283973
C	-5.842810	-2.368386	1.387943
C	-2.744956	-0.994467	-3.437328
C	-1.970501	-1.560469	-4.620262
C	-4.853130	0.396765	-0.533787
C	-4.629166	1.637159	-0.045849
C	-3.779629	1.528103	4.188832
C	-3.924071	4.663305	-1.718782
C	1.018414	3.264841	2.173539
C	7.326299	0.602269	-0.356717
C	-3.616957	-2.818069	2.444809
C	-4.045161	-0.351522	-3.929527
C	1.568773	-4.030392	4.512342
C	1.639204	-3.430078	-2.999113
H	4.586539	-2.892940	1.906055
H	-5.355721	2.389961	0.252644
H	-5.801124	-0.083085	-0.765486
H	5.546208	-0.358259	1.836507
H	2.262981	-1.971998	-1.589841
H	2.732431	-2.680813	3.340830
H	-2.961672	-5.388477	-1.780891
H	1.146183	3.438556	-1.315440
H	-2.127926	-0.198853	-2.987539
H	3.280842	2.388466	-2.185456
H	6.246650	3.350411	-1.161701
H	-2.346104	3.764868	4.113061
H	-1.713425	5.926670	3.087355
H	-2.434263	-3.722881	-3.525461
H	-0.268772	-6.272808	0.499483
H	0.681999	-5.236514	-1.533056
H	-4.163095	-1.047057	1.377694
H	5.419053	-0.282784	-0.765128
H	-3.862940	-4.655451	0.407925
H	-2.193581	3.403180	-1.718534
H	0.615211	-0.701981	-3.639429
H	2.914855	4.624923	1.215561
H	0.236757	-5.332589	2.733218
H	3.001288	0.146673	-3.430373
H	4.862423	5.168045	-0.207762
H	-1.547745	5.436677	-2.896104
H	-0.506603	5.260722	-1.465375
H	-1.841091	6.444942	-1.474281
H	1.543699	1.282367	1.644175
H	4.408548	-3.045748	-0.926409
H	4.293464	-2.853424	-2.689467
H	4.026225	-4.454569	-1.952094
H	-1.736881	6.192314	0.633195
H	-3.740754	0.921712	2.152462
H	-0.741419	1.265718	-4.555725
H	4.982095	0.937245	-2.905816
H	6.506383	0.014352	-2.930873
H	6.538879	1.777749	-2.741706
H	-0.445908	3.478468	-3.323335
H	1.772386	-4.491523	-3.263977
H	2.036274	-2.832468	-3.832388
H	0.558959	-3.225932	-2.933543
H	-2.568979	-2.291407	-5.188145
H	-1.034257	-2.047045	-4.311654
H	-1.704348	-0.749276	-5.312517
H	-3.820859	0.412180	-4.689920
H	-4.596544	0.139888	-3.117445
H	-4.702585	-1.106678	-4.390081
H	7.877899	1.540493	-0.529027
H	7.901861	-0.214532	-0.820404
H	7.306792	0.429577	0.729517
H	-4.287571	5.599961	-1.265651
H	-4.615850	3.857223	-1.439354
H	-3.968309	4.776153	-2.813556
H	3.128672	2.621892	3.899215
H	3.303717	0.929382	3.371412
H	1.776889	1.479841	4.106480
H	0.174893	2.925653	2.789153
H	0.609727	3.547891	1.192148
H	1.420064	4.171015	2.654417
H	-1.082750	1.058029	3.679537
H	-1.263603	0.552250	1.988948
H	-2.011794	-0.409400	3.307690
H	-4.685341	2.149448	4.123409
H	-3.135987	1.950237	4.976673
H	-4.079682	0.522686	4.524057
H	-0.287403	-2.218344	3.478300
H	0.906480	-1.156741	2.659103
H	0.942724	-1.351479	4.416288
H	-6.076612	-3.442105	1.306346
H	-6.388482	-1.846376	0.590254
H	-6.228790	-2.008658	2.354444
H	-3.805714	-3.902445	2.467019
H	-3.966011	-2.406670	3.404032
H	-2.524859	-2.677175	2.417074
H	1.857268	-3.516058	5.441180
H	2.211081	-4.917186	4.402549
H	0.531421	-4.376975	4.646892
K	-0.755042	-2.041722	0.336361

Anion of 2: TS, OA

145

scf done: -2917.04338170

C	-4.542412	-1.251258	-2.215085
C	-4.225565	-0.793654	-0.917163
C	-4.241878	-1.668216	0.192444
C	-4.558699	-3.010860	-0.030255
C	-4.874142	-3.476399	-1.302476
C	-4.873986	-2.599512	-2.381288
N	-3.825245	0.547305	-0.723722

C	-4.605717	1.673109	-1.000294
C	-3.860183	2.794303	-0.839702
N	-2.564266	2.446773	-0.460257
C	-1.526405	3.367686	-0.178374
C	-0.401521	3.413340	-1.030240
C	0.625433	4.309975	-0.721779
C	0.537397	5.144098	0.387098
C	-0.571418	5.079221	1.222686
C	-1.614754	4.185629	0.967144
C	-0.333879	2.544839	-2.273005
C	-1.224977	3.124537	-3.374057
C	-2.757352	4.011583	1.948900
C	-2.486862	2.795169	2.836638
C	-3.963397	-1.146158	1.590673
C	-5.137690	-0.306963	2.099219
C	-4.441809	-0.326344	-3.416472
C	-2.998697	-0.243650	-3.914115
B	-2.498956	1.004252	-0.350775
O	-1.455112	0.247963	0.006579
Al	0.033781	0.068067	0.859107
O	1.340188	-0.161766	-0.263242
B	2.403780	-0.976100	-0.143350
N	3.772725	-0.684260	-0.503731
C	4.242175	0.495090	-1.127275
C	4.579192	0.468785	-2.495538
C	5.003567	1.655593	-3.101628
C	5.068014	2.842151	-2.380059
C	4.708965	2.858359	-1.035397
C	4.296001	1.693869	-0.382739
C	4.369168	-0.788322	-3.319022
C	2.975227	-0.765102	-3.949363
C	3.934609	1.694227	1.086576
C	3.501552	3.044890	1.637345
C	4.534889	-1.832983	-0.272943
C	3.738925	-2.819613	0.208959
N	2.427153	-2.355295	0.315267
C	1.286820	-3.147416	0.598401
C	1.043616	-3.631213	1.904313
C	-0.119133	-4.373894	2.129509
C	-1.036461	-4.613672	1.112697
C	-0.793186	-4.125444	-0.164589
C	0.371872	-3.407225	-0.448959
C	2.013138	-3.392028	3.043723
C	1.318119	-3.017959	4.350484
C	0.648270	-2.986091	-1.878058
C	0.995030	-4.204672	-2.734545
C	5.450319	-1.017615	-4.370007
C	5.067629	1.100710	1.924781
C	-0.494861	-2.178288	-2.480565
C	2.917490	-4.611620	3.242553
C	1.081750	2.307899	-2.782019
C	-3.038526	5.249457	2.792889
C	-3.595379	-2.230830	2.594049
C	-5.388754	-0.693879	-4.553969
C	0.343042	0.296038	2.708455
C	1.713480	0.149350	3.269876
C	1.970041	0.641143	4.530847
C	0.931675	1.057143	5.396471
C	-0.377994	0.674663	5.033402
C	-0.676731	0.180765	3.779554
H	-5.658374	1.591080	-1.262929
H	5.609696	-1.848380	-0.443212
H	4.014927	-3.831306	0.495529
H	-4.158582	3.832612	-0.970408
H	-3.090401	-0.482519	1.514746
H	-4.716038	0.684718	-3.082235
H	-1.950827	-5.176096	1.318323
H	2.514821	-0.325295	2.694799
H	2.642807	-2.537692	2.754401
H	-0.612876	5.716637	2.107321
H	5.264060	1.655522	-4.162676
H	5.390229	3.764199	-2.871403
H	-0.320608	-4.746795	3.135804
H	-5.121679	-4.530453	-1.453706
H	-4.561607	-3.705819	0.810576
H	1.537242	-2.341463	-1.865309
H	-3.668556	3.797920	1.368138
H	-1.520884	-4.305794	-0.959623
H	3.068640	1.029519	1.206250
H	-1.168780	0.691393	5.796023
H	1.513322	4.348680	-1.354723
H	-5.118588	-2.974900	-3.376911
H	-1.669712	-0.227537	3.571092
H	1.352090	5.835974	0.615238
H	3.096259	2.900947	2.650591
H	2.703442	3.490938	1.026828
H	4.338447	3.761434	1.701672
H	-0.738057	1.558528	-1.998728
H	-5.366350	0.524178	1.416694
H	-4.899335	0.122936	3.084843
H	-6.043510	-0.926983	2.204019
H	4.747862	3.795397	-0.476821
H	4.397205	-1.645546	-2.630251
H	1.151899	1.422715	6.402403
H	-2.355643	1.882114	2.241627
H	-3.322846	2.621485	3.533091
H	-1.566203	2.915313	3.427416
H	3.001765	0.614038	4.908787
H	-4.441582	-2.905859	2.805760
H	-3.293774	-1.771215	3.546620
H	-2.745893	-2.833209	2.240962
H	0.746094	-3.864793	4.766413
H	0.649873	-2.152812	4.217087
H	2.068761	-2.725964	5.100450
H	3.657609	-4.418020	4.035464
H	3.464127	-4.880045	2.326150
H	2.324386	-5.491797	3.542225
H	-2.222187	5.452476	3.504057
H	-3.950862	5.096626	3.389972
H	-3.182063	6.148179	2.172191
H	5.973230	1.726810	1.857566
H	5.326299	0.084612	1.592410
H	4.760033	1.044339	2.980112
H	-0.870915	4.126112	-3.668718
H	-2.270102	3.213769	-3.043034
H	-1.206081	2.479910	-4.267371
H	1.058587	1.588275	-3.613540
H	1.729872	1.889507	-1.998650
H	1.548859	3.229196	-3.165761
H	2.891378	0.054079	-4.682146
H	2.200450	-0.606741	-3.186028
H	2.760204	-1.713768	-4.467489
H	6.455699	-1.012728	-3.921282
H	5.431060	-0.248840	-5.159324
H	5.301408	-1.990593	-4.864347
H	-2.654245	-1.223274	-4.279973
H	-2.318579	0.071026	-3.111231
H	-2.911270	0.483172	-4.737318
H	0.145905	-4.905080	-2.794807
H	1.853648	-4.751989	-2.316216

H	1.251891	-3.896663	-3.761320
H	-1.417727	-2.773294	-2.574253
H	-0.224791	-1.823180	-3.487754
H	-0.728582	-1.302920	-1.859499
H	-5.365349	0.086134	-5.330928
H	-6.427402	-0.795929	-4.202978
H	-5.102878	-1.639889	-5.041493
H	0.272996	1.449376	2.231147

Anion of 2: TS, C-C insertion

145

scf done: -2917.05475270

C	3.856991	-4.209350	-1.674440
C	4.077146	-3.005336	-0.998733
C	3.130505	-2.591245	-0.038272
C	1.972283	-3.357268	0.218798
C	1.783454	-4.536920	-0.502135
C	2.719143	-4.968491	-1.437508
N	3.316254	-1.375283	0.656105
C	4.452992	-1.083455	1.407860
C	4.458667	0.227666	1.743615
N	3.324755	0.852586	1.218487
B	2.528037	-0.151032	0.515320
O	1.350087	0.019962	-0.067146
Al	-0.052560	-0.218499	-1.084223
O	-1.492007	-0.100787	-0.070771
B	-2.712418	0.339534	0.204885
N	-3.755167	-0.349263	0.960050
C	-4.878359	0.478808	1.028746
C	-4.637416	1.628873	0.354527
N	-3.342444	1.606648	-0.161814
C	-2.893613	2.493441	-1.169270
C	-3.261607	2.249206	-2.507781
C	-2.773547	3.105643	-3.499120
C	-1.929882	4.161804	-3.176214
C	-1.581606	4.395804	-1.848879
C	-2.066500	3.580473	-0.822825
C	-4.174917	1.092924	-2.868551
C	-5.586306	1.594777	-3.176400
C	-1.710143	3.829960	0.629085
C	-0.521360	2.974003	1.062337
C	0.990811	-2.927585	1.293280
C	-0.421368	-3.452579	1.081980
C	5.313492	-2.184837	-1.324125
C	5.387267	-1.794886	-2.798200
C	-3.744853	-1.662452	1.480815
C	-3.601317	-1.852266	2.869750
C	-3.639173	-3.154888	3.374931
C	-3.794007	-4.243835	2.523403
C	-3.895120	-4.045277	1.150609
C	-3.869038	-2.759294	0.603431
C	-3.306542	-0.660791	3.757690
C	-1.816821	-0.320493	3.671784
C	-3.980397	-2.532691	-0.891915
C	-3.498885	-3.711876	-1.728395
C	3.075390	2.231485	1.380127
C	2.832525	2.743100	2.673383
C	2.633253	4.118541	2.821269
C	2.648960	4.964385	1.717693
C	2.856317	4.441790	0.445319
C	3.073096	3.074555	0.246971
C	2.691655	1.793740	3.849746
C	2.954695	2.439840	5.204585
C	3.303040	2.502491	-1.139961
C	2.833902	3.416043	-2.264066
C	-3.633207	0.230438	-4.006036
C	-1.451014	5.300199	0.942780
C	-5.407259	-2.137362	-1.279078
C	-3.746726	-0.840972	5.205366
C	1.523264	-3.295331	2.679655
C	1.314664	1.128798	3.819609
C	6.586022	-2.905584	-0.877866
C	4.766495	2.103076	-1.343803
H	-5.772933	0.179665	1.572092
H	-5.292948	2.485987	0.212180
H	-3.040364	2.926358	-4.543810
H	-1.536346	4.808198	-3.964980
H	-4.245336	0.452571	-1.977669
H	-0.921796	5.231345	-1.606748
H	-2.583165	3.505883	1.219897
H	-3.317882	-1.684941	-1.126598
H	-3.859600	0.198048	3.345895
H	-3.530629	-3.322793	4.448383
H	-3.985880	-4.909253	0.489823
H	-3.566623	0.791231	-4.952498
H	-4.303306	-0.626444	-4.182182
H	-2.631232	-0.160459	-3.776831
H	-3.815477	-5.257811	2.931339
H	-0.513052	5.652974	0.484817
H	-1.341091	5.437786	2.029697
H	-2.270147	5.948634	0.593566
H	5.183278	-1.849926	1.658551
H	-0.720495	1.903453	0.933374
H	-0.278142	3.155976	2.119886
H	0.373882	3.217873	0.472999
H	5.206496	0.780995	2.308139
H	2.443994	4.534203	3.812922
H	2.483350	6.037159	1.847880
H	0.925822	-1.831847	1.250408
H	-3.154437	-1.613422	5.722253
H	-4.808476	-1.124455	5.277639
H	-3.605075	0.097664	5.763349
H	2.845485	5.111827	-0.415704
H	3.434311	0.991828	3.721328
H	0.884337	-5.130616	-0.329002
H	-6.113084	-2.949341	-1.037455
H	-5.474158	-1.938860	-2.360710
H	-5.732592	-1.230429	-0.749314
H	0.522406	1.872933	3.997272
H	1.234053	0.352106	4.596710
H	1.115038	0.660151	2.845759
H	5.248350	-1.246811	-0.755732
H	2.551346	-5.895271	-1.992525
H	-5.999499	2.163303	-2.330021
H	-6.264012	0.750103	-3.381480
H	-5.588250	2.254414	-4.060104
H	2.693039	1.588815	-1.214262
H	-1.590244	0.596155	4.237617
H	-1.497039	-0.168852	2.630766
H	-1.207806	-1.138439	4.090511
H	4.581319	-4.540707	-2.423843
H	-2.484260	-4.023226	-1.439860
H	-3.469585	-3.427364	-2.791181
H	-4.168210	-4.583892	-1.641342
H	6.557075	-3.150284	0.195197
H	7.475789	-2.281603	-1.062293
H	6.720776	-3.852297	-1.426579
H	3.930746	2.949161	5.232658
H	2.946526	1.673959	5.995771
H	2.180233	3.179305	5.465762

H	4.889020	1.602168	-2.317110
H	5.111225	1.409460	-0.563328
H	5.421029	2.990848	-1.326523
H	5.450875	-2.676966	-3.456127
H	6.280156	-1.175483	-2.983401
H	4.498644	-1.219067	-3.088822
H	3.433125	4.340207	-2.329864
H	1.774221	3.689838	-2.145689
H	2.926732	2.887124	-3.222977
H	1.615356	-4.389034	2.786708
H	0.839526	-2.932063	3.464395
H	2.513264	-2.848901	2.857046
H	-0.787145	-3.194097	0.077467
H	-1.109442	-2.999135	1.809531
H	-0.489056	-4.545593	1.209461
C	-0.258915	-1.550145	-2.465342
C	-0.061851	0.543318	-2.858500
C	0.760476	-2.211446	-3.197720
C	1.928249	-1.647216	-3.666162
C	2.083583	-0.250388	-3.898321
C	1.073039	0.672995	-3.700161
H	0.577876	-3.257065	-3.483816
H	-1.268908	-1.969154	-2.628788
H	2.669651	-2.313728	-4.118525
H	2.933381	0.054498	-4.520985
H	1.110153	1.568250	-4.338595
H	-0.945170	1.085439	-3.230577

Anion of 2: TS, [4+1] cycloaddition

145

scf done: -2917.05243818

C	-2.986681	2.046941	2.488899
C	-3.289315	1.749000	1.142848
C	-3.322550	2.760074	0.162984
C	-3.076771	4.080808	0.550642
C	-2.792494	4.390996	1.873709
C	-2.742552	3.379956	2.829647
N	-3.551930	0.414294	0.756402
C	-4.838384	-0.030376	0.452324
C	-4.775110	-1.251426	-0.129941
N	-3.441801	-1.650158	-0.234060
C	-3.062953	-2.840547	-0.889098
C	-3.173422	-2.914920	-2.293199
C	-2.861240	-4.121905	-2.925136
C	-2.421640	-5.216813	-2.188831
C	-2.262956	-5.111026	-0.810733
C	-2.572468	-3.926415	-0.135779
C	-3.505293	-1.665425	-3.086121
C	-4.164586	-1.941120	-4.432036
C	-2.375401	-3.783155	1.362318
C	-3.708455	-3.792373	2.113507
C	-3.596881	2.431706	-1.290521
C	-2.502330	2.975464	-2.201480
C	-2.950439	0.943238	3.529158
C	-4.361686	0.488362	3.906901
B	-2.586908	-0.591774	0.303405
O	-1.260009	-0.555586	0.308366
Al	0.121522	-0.140263	1.402595
O	1.345837	0.253866	0.154448
B	2.417961	0.626259	-0.519761
N	3.585879	-0.172617	-0.923462
C	3.905802	-1.505791	-0.595781
C	3.025932	-2.546030	-0.979149
C	3.356540	-3.858126	-0.633925
C	4.532688	-4.156094	0.047935
C	5.386676	-3.127353	0.420891
C	5.082993	-1.792991	0.131845
C	1.778994	-2.250929	-1.790812
C	0.679608	-3.290279	-1.626566
C	5.990623	-0.699989	0.668293
C	7.331087	-0.664506	-0.066745
C	4.447080	0.649150	-1.653108
C	3.925217	1.892869	-1.754004
N	2.700668	1.947718	-1.088707
C	1.844308	3.066615	-1.163284
C	1.482311	3.747567	0.018775
C	0.652942	4.867759	-0.090871
C	0.216032	5.322668	-1.329754
C	0.564383	4.631584	-2.484965
C	1.354350	3.480634	-2.423534
C	2.017705	3.289666	1.362567
C	1.195374	3.778416	2.546851
C	1.590814	2.623610	-3.655423
C	1.476208	3.381859	-4.972252
C	2.144446	-2.052923	-3.262972
C	6.213090	-0.831685	2.175898
C	0.650485	1.415471	-3.641285
C	3.491550	3.670117	1.518213
C	-2.239778	-0.818572	-3.255787
C	-1.426393	-4.820368	1.949537
C	-4.980930	2.914704	-1.722059
C	-2.151947	1.313592	4.769854
C	1.480787	0.164682	4.072856
C	0.626634	-0.831439	4.629516
C	0.470733	-2.005777	3.942883
C	1.161939	-2.144501	2.694995
C	2.417062	-1.465655	2.582149
C	2.557649	-0.275245	3.257254
H	4.352345	2.768911	-2.238570
H	-5.590067	-1.898581	-0.449482
H	-5.718570	0.560158	0.699839
H	5.382588	0.274593	-2.061105
H	1.949947	2.191059	1.372407
H	2.615597	2.227113	-3.597616
H	-2.589109	5.425509	2.163177
H	1.018144	-3.059189	2.112108
H	-2.429876	0.090526	3.059215
H	3.160094	-1.809249	1.862338
H	6.295699	-3.357919	0.982776
H	-2.943690	-4.202061	-4.011061
H	-2.176608	-6.153923	-2.695753
H	-2.494932	3.634363	3.861113
H	-0.421151	6.208940	-1.392989
H	0.346129	5.397011	0.812176
H	-3.585605	1.335735	-1.380462
H	5.483066	0.260512	0.495485
H	-3.086524	4.873163	-0.202146
H	-1.910085	-2.794978	1.515753
H	1.541076	1.144520	4.553826
H	2.679380	-4.667181	-0.912958
H	0.183190	4.973591	-3.449048
H	3.422859	0.373068	3.089988
H	4.773874	-5.191728	0.301079
H	-1.209451	-4.577932	3.000372
H	-0.468111	-4.848831	1.409545
H	-1.859697	-5.834332	1.929961
H	1.363426	-1.308051	-1.411221
H	4.100119	3.264303	0.696888
H	3.892735	3.273577	2.465166
H	3.615068	4.766175	1.528387

H	-1.881953	-5.966685	-0.250852
H	-4.215183	-1.073055	-2.487280
H	0.044437	-0.616485	5.528811
H	5.261605	-0.904964	2.721355
H	6.762480	0.043578	2.558629
H	6.807781	-1.725815	2.423573
H	-0.249039	-2.764197	4.264527
H	1.259999	4.872066	2.678187
H	1.567607	3.312678	3.471273
H	0.137052	3.496712	2.440959
H	-2.625243	2.131003	5.339197
H	-1.127493	1.607529	4.496950
H	-2.084064	0.444944	5.441061
H	-4.316400	-0.337366	4.635515
H	-4.921703	0.131576	3.031576
H	-4.928546	1.315630	4.366118
H	7.884948	-1.606208	0.081611
H	7.962025	0.158617	0.306170
H	7.200586	-0.527877	-1.150380
H	-4.218469	-4.762315	1.989054
H	-4.380614	-3.000330	1.755419
H	-3.541985	-3.630546	3.190700
H	2.579368	-2.974004	-3.685956
H	2.875734	-1.239944	-3.387096
H	1.248735	-1.796788	-3.850983
H	-0.243255	-2.948860	-2.116154
H	0.437022	-3.447623	-0.565793
H	0.941965	-4.264872	-2.070562
H	-1.496895	-1.351370	-3.872037
H	-1.768318	-0.592335	-2.287626
H	-2.471577	0.135333	-3.755501
H	-5.054959	-2.580752	-4.327113
H	-3.474677	-2.434368	-5.136004
H	-4.477511	-0.994854	-4.900347
H	-0.392743	1.734209	-3.783152
H	0.703391	0.872222	-2.686960
H	0.905964	0.711526	-4.448949
H	-5.062881	4.011366	-1.640212
H	-5.770705	2.470212	-1.098193
H	-5.181040	2.638907	-2.770402
H	-2.482651	4.076378	-2.212886
H	-2.658426	2.637370	-3.238700
H	-1.510825	2.635321	-1.872244
H	1.766296	2.728315	-5.809777
H	2.126863	4.270290	-4.991510
H	0.443725	3.714507	-5.167474

Anion of 2: product, OA

145

scf done: -2917.13863625

C	2.925407	1.414382	-3.382473
C	3.088031	1.509408	-1.983592
C	2.978465	2.748909	-1.318295
C	2.736026	3.891549	-2.087468
C	2.604443	3.815921	-3.470530
C	2.692852	2.584609	-4.110343
N	3.374498	0.340478	-1.246302
C	4.594037	-0.327567	-1.379955
C	4.583529	-1.451908	-0.626070
N	3.354966	-1.572647	0.019301
C	3.065134	-2.626343	0.915573
C	2.112639	-3.599261	0.552153
C	1.858863	-4.641806	1.445996
C	2.521478	-4.717657	2.665945
C	3.437392	-3.734173	3.024313
C	3.717286	-2.667856	2.165173
C	1.389902	-3.500280	-0.777760
C	2.284791	-3.976606	-1.923567
C	4.616734	-1.522790	2.594130
C	3.768710	-0.367262	3.129873
C	3.120697	2.822493	0.190473
C	4.571162	2.606344	0.629234
C	2.907865	0.051680	-4.049968
C	1.530296	-0.590673	-3.879302
B	2.516167	-0.429411	-0.341446
O	1.275402	-0.151464	0.037483
Al	-0.009958	-0.486230	1.235734
O	-1.530618	-0.104842	0.424968
B	-2.666016	0.179673	-0.180324
N	-3.650583	-0.731402	-0.763389
C	-3.601785	-2.139492	-0.823377
C	-3.493769	-2.780270	-2.076353
C	-3.493837	-4.177236	-2.115210
C	-3.558300	-4.924246	-0.943936
C	-3.613074	-4.280057	0.287458
C	-3.640568	-2.885341	0.374688
C	-3.258129	-1.956250	-3.328104
C	-1.784946	-1.548529	-3.399890
C	-3.736353	-2.177254	1.712913
C	-3.222398	-3.003423	2.884627
C	-4.702880	0.028948	-1.280653
C	-4.467110	1.345944	-1.058129
N	-3.253367	1.496441	-0.392505
C	-2.728996	2.722433	0.074694
C	-2.890530	3.065820	1.432394
C	-2.342818	4.271441	1.879351
C	-1.642670	5.103474	1.012354
C	-1.469538	4.739635	-0.320343
C	-2.004596	3.545220	-0.810917
C	-3.647048	2.141311	2.367078
C	-3.203083	2.234635	3.821765
C	-1.760974	3.080089	-2.232487
C	-1.528109	4.216658	-3.221228
C	-3.699923	-2.639925	-4.616331
C	-5.160341	-1.677148	1.963782
C	-0.597156	2.087736	-2.268143
C	-5.156596	2.357966	2.240409
C	0.042736	-4.209334	-0.795503
C	5.688758	-1.925324	3.600566
C	2.577311	4.113138	0.789958
C	3.322320	0.074258	-5.516356
C	0.225241	0.773455	2.770176
C	0.310344	0.307205	4.094067
C	0.474915	1.170261	5.180126
C	0.564923	2.546356	4.966990
C	0.478198	3.043484	3.666135
C	0.306765	2.165972	2.593493
H	5.395155	0.070138	-2.000252
H	-5.568931	-0.436397	-1.747664
H	-5.088234	2.200903	-1.319689
H	5.367632	-2.196931	-0.503078
H	2.517125	1.994623	0.597883
H	3.628177	-0.586839	-3.515969
H	-1.210631	6.037123	1.382784
H	0.249495	-0.771809	4.280641
H	-3.428932	1.116689	2.028679
H	3.929286	-3.787706	3.997743
H	-3.415883	-4.691142	-3.075473
H	-3.544091	-6.016355	-0.989932
H	-2.442194	4.550410	2.929732

H	2.415162	4.722448	-4.051549
H	2.642227	4.860448	-1.594847
H	-2.667187	2.539214	-2.550273
H	5.135239	-1.153587	1.695599
H	-0.898496	5.389454	-0.986675
H	-3.077992	-1.299615	1.644248
H	0.527925	4.121705	3.485934
H	1.116818	-5.399654	1.189658
H	2.561905	2.531088	-5.193017
H	0.215445	2.586179	1.586795
H	2.307182	-5.540437	3.353414
H	-3.202106	-2.382817	3.793810
H	-2.195424	-3.352895	2.700550
H	-3.863629	-3.875286	3.098222
H	1.184652	-2.430616	-0.937623
H	4.966341	1.644400	0.274364
H	4.639866	2.614030	1.728508
H	5.218069	3.410644	0.240489
H	-3.635413	-4.874319	1.202583
H	-3.841760	-1.027991	-3.232426
H	0.695124	3.229130	5.811956
H	3.020176	-0.037643	2.396551
H	4.399473	0.499942	3.383285
H	3.218532	-0.667473	4.035091
H	0.535270	0.770398	6.197462
H	3.185472	4.987027	0.501191
H	2.593781	4.045390	1.887207
H	1.536259	4.301221	0.485764
H	-3.478432	3.200748	4.276923
H	-2.117125	2.100823	3.923069
H	-3.698649	1.447832	4.411896
H	-5.704897	1.655451	2.888834
H	-5.501479	2.200038	1.208607
H	-5.427474	3.383552	2.542039
H	5.255510	-2.194324	4.577521
H	6.376730	-1.083563	3.776573
H	6.281417	-2.783392	3.246053
H	-5.865921	-2.521766	2.035637
H	-5.500295	-1.013066	1.155497
H	-5.209796	-1.110599	2.907989
H	2.537187	-5.043332	-1.803031
H	3.223358	-3.404410	-1.966275
H	1.770099	-3.856789	-2.890809
H	-0.491092	-3.987482	-1.731268
H	-0.596161	-3.870051	0.032733
H	0.145019	-5.305739	-0.733900
H	-1.141702	-2.432180	-3.540297
H	-1.461832	-1.044718	-2.478237
H	-1.607477	-0.861675	-4.242306
H	-4.749188	-2.970509	-4.564471
H	-3.079453	-3.520522	-4.849225
H	-3.602152	-1.945647	-5.465512
H	0.769279	-0.026912	-4.441326
H	1.220323	-0.603682	-2.824803
H	1.530144	-1.627670	-4.250854
H	-0.570765	4.728298	-3.032332
H	-2.333461	4.967504	-3.185214
H	-1.475550	3.817823	-4.246301
H	0.349123	2.589803	-2.021218
H	-0.490476	1.651255	-3.272702
H	-0.727644	1.271062	-1.546153
H	3.397121	-0.954037	-5.903233
H	4.298668	0.563898	-5.657388
H	2.586337	0.600853	-6.145574
H	-0.038683	-2.016267	1.744723

Anion of 2: product, C-C insertion

145

scf done: -2917.09452930

C	2.751479	-4.216399	-2.407581
C	3.283231	-3.104230	-1.753408
C	2.820414	-2.805935	-0.453706
C	1.847098	-3.603851	0.176451
C	1.334041	-4.702916	-0.521309
C	1.780405	-5.009208	-1.800546
N	3.307171	-1.658063	0.207583
C	4.557310	-1.559996	0.812659
C	4.704619	-0.323431	1.348948
N	3.557559	0.434775	1.101828
B	2.600465	-0.391123	0.368613
O	1.380734	-0.066131	-0.023380
Al	0.060070	-0.075836	-1.212195
O	-1.393943	-0.043807	-0.185654
B	-2.578015	0.415831	0.185307
N	-3.634586	-0.305404	0.896408
C	-4.721453	0.554557	1.071558
C	-4.439457	1.762715	0.526584
N	-3.153196	1.743678	-0.008096
C	-2.617492	2.789369	-0.793419
C	-2.913448	2.835041	-2.168731
C	-2.334467	3.846906	-2.940423
C	-1.489037	4.787419	-2.365440
C	-1.215136	4.737972	-1.000426
C	-1.775304	3.746774	-0.190571
C	-3.824714	1.806114	-2.809177
C	-5.042665	2.465899	-3.454550
C	-1.465578	3.660614	1.290942
C	-0.283115	2.726472	1.549384
C	1.356252	-3.296497	1.576400
C	-0.131345	-2.956506	1.597286
C	4.280624	-2.183186	-2.431257
C	3.569725	-0.984084	-3.061976
C	-3.621852	-1.626464	1.390990
C	-3.611771	-1.840114	2.786180
C	-3.656217	-3.153808	3.261246
C	-3.681925	-4.230082	2.380849
C	-3.643912	-4.005888	1.008454
C	-3.609981	-2.709540	0.486284
C	-3.446149	-0.661970	3.728727
C	-1.976843	-0.234818	3.771193
C	-3.562438	-2.455074	-1.008792
C	-3.081122	-3.651432	-1.819723
C	3.375931	1.741137	1.602025
C	3.177023	1.924915	2.986468
C	3.040465	3.227173	3.475199
C	3.073732	4.318285	2.613198
C	3.222692	4.119293	1.244130
C	3.369183	2.834846	0.711028
C	3.007146	0.714363	3.885296
C	3.372958	0.967751	5.342723
C	3.500969	2.601589	-0.782111
C	3.007483	3.767478	-1.629490
C	-3.075785	0.915149	-3.798054
C	-1.225229	5.021172	1.937779
C	-4.910945	-1.955565	-1.532326
C	-3.983031	-0.910183	5.133137
C	1.684647	-4.437882	2.538960
C	1.577958	0.177707	3.763204
C	5.162299	-2.889802	-3.455344
C	4.932978	2.224472	-1.166778

H	-5.635808	0.224797	1.561479
H	-5.068202	2.649280	0.467996
H	-2.534295	3.883537	-4.014584
H	-1.031319	5.563113	-2.985099
H	-4.195418	1.157892	-2.002137
H	-0.548765	5.482198	-0.558992
H	-2.352062	3.211468	1.768085
H	-2.820118	-1.655951	-1.159859
H	-4.012175	0.182423	3.306082
H	-3.652792	-3.340438	4.337070
H	-3.636823	-4.858338	0.326984
H	-2.650420	1.497098	-4.631079
H	-3.755803	0.159998	-4.224725
H	-2.239035	0.391223	-3.316073
H	-3.712144	-5.252380	2.767209
H	-0.279764	5.472176	1.595690
H	-1.143792	4.908734	3.030206
H	-2.040661	5.730135	1.723669
H	5.257640	-2.393371	0.819449
H	-0.455544	1.721741	1.144040
H	-0.090906	2.633672	2.629422
H	0.634707	3.115449	1.084689
H	5.559138	0.092026	1.880093
H	2.887364	3.391144	4.543912
H	2.960808	5.331316	3.008566
H	1.906975	-2.409065	1.920375
H	-3.391806	-1.668140	5.672391
H	-5.030965	-1.248562	5.116461
H	-3.933032	0.016484	5.725988
H	3.214783	4.980951	0.574422
H	3.680493	-0.071359	3.508843
H	0.565876	-5.323462	-0.051482
H	-5.686306	-2.731181	-1.414668
H	-4.837126	-1.706630	-2.603046
H	-5.245339	-1.054266	-0.998886
H	0.857572	0.896210	4.185289
H	1.467412	-0.773931	4.307997
H	1.295850	0.006970	2.714274
H	4.943162	-1.790312	-1.643694
H	1.363528	-5.866682	-2.335500
H	-5.597323	3.079039	-2.727210
H	-5.730008	1.701914	-3.852423
H	-4.753258	3.120363	-4.293225
H	2.850119	1.744011	-1.016836
H	-1.852884	0.687841	4.360819
H	-1.580561	-0.049253	2.763195
H	-1.356561	-1.019568	4.233063
H	3.084218	-4.457660	-3.418975
H	-2.126741	-4.042234	-1.436224
H	-2.911264	-3.345924	-2.862840
H	-3.816777	-4.473703	-1.825472
H	5.655002	-3.777959	-3.028982
H	5.944700	-2.204308	-3.817074
H	4.586630	-3.212562	-4.337841
H	4.387974	1.383304	5.442918
H	3.331882	0.026134	5.912264
H	2.672174	1.666604	5.827902
H	4.994423	2.009285	-2.245367
H	5.275449	1.331089	-0.625741
H	5.626984	3.052032	-0.942931
H	2.890823	-1.300575	-3.867202
H	4.302822	-0.276282	-3.481234
H	2.956674	-0.443765	-2.328522
H	3.670415	4.645999	-1.551864
H	1.989746	4.074850	-1.345638
H	2.973388	3.465689	-2.686830
H	1.150052	-5.362742	2.266391
H	1.384679	-4.173610	3.565981
H	2.762875	-4.661668	2.542825
H	-0.357609	-2.081413	0.972985
H	-0.463113	-2.726202	2.621315
H	-0.748388	-3.795652	1.239874
C	-0.036111	-1.697240	-2.320379
C	0.287485	1.496298	-2.369161
C	0.032854	-1.825279	-3.670187
C	0.250850	-0.833108	-4.709566
C	0.441139	0.518257	-4.725154
C	0.458055	1.561390	-3.714094
H	-0.062083	-2.837661	-4.095929
H	-0.181639	-2.665893	-1.817867
H	0.283092	-1.282745	-5.710723
H	0.595499	0.918463	-5.735834
H	0.605739	2.556103	-4.166103
H	0.314715	2.489797	-1.895958

Anion of 2: product, [4+1] cycloaddition

145

scf done: -2917.10116582

Al	0.029649	-0.432198	-1.081091
O	-1.383869	0.008776	-0.114294
O	1.372788	-0.164781	0.035843
N	3.745520	-0.669067	0.608997
N	3.344287	1.350188	-0.413780
N	-3.496817	-1.290248	0.346714
N	-3.498295	0.959792	0.809909
C	2.745685	2.536786	-0.899095
C	-3.189129	2.319513	1.013136
C	3.622322	-1.853333	1.370902
C	1.887464	3.264470	-0.040596
C	-3.264808	-2.560564	-0.222617
C	3.299861	-1.760404	2.740528
C	-3.184120	2.840552	2.326975
C	2.990760	2.982892	-2.214760
C	-2.224067	-3.368321	0.288005
C	-4.732283	0.406451	1.155905
H	-5.541096	1.014872	1.555566
C	-2.888322	3.135143	-0.099725
C	4.934542	0.055635	0.518969
H	5.872809	-0.333163	0.909827
C	3.804572	-3.107769	0.754397
C	4.698353	1.242602	-0.089015
H	5.403869	2.041887	-0.301389
C	-4.730965	-0.918084	0.879122
H	-5.529760	-1.642667	1.024399
C	-2.882216	2.548957	-1.499629
H	-2.386923	1.570224	-1.427197
C	-3.334463	1.904931	3.514876
H	-4.116354	1.172174	3.264243
C	1.507813	4.866478	-1.824652
H	1.012506	5.769411	-2.191383
C	0.026483	-2.170824	-2.192855
H	-0.059660	-3.191257	-1.804900
C	3.903583	2.223402	-3.157023
H	3.976563	1.197160	-2.763737
C	-1.118430	-1.728720	-3.055528
H	-1.937539	-2.407216	-3.307217
C	-3.821897	-4.266210	-1.837672
H	-4.426724	-4.614102	-2.679931
C	3.212091	-2.936983	3.490405
H	2.957063	-2.879773	4.551766

C	3.426211	-4.177715	2.901250
H	3.350892	-5.089366	3.499941
C	2.370509	4.159181	-2.650583
H	2.550450	4.512575	-3.668486
C	-2.678138	5.028932	1.408917
H	-2.482501	6.093530	1.562567
C	-2.629859	4.490452	0.127233
H	-2.390016	5.137413	-0.718279
C	1.700488	2.836584	1.402125
H	1.604303	1.741850	1.399608
C	-5.120981	-2.109628	-1.933677
H	-5.136410	-1.162270	-1.377709
C	1.266150	4.414920	-0.530862
H	0.577960	4.967423	0.110424
C	4.098189	-3.194446	-0.729058
H	3.656761	-2.294563	-1.185162
C	0.200860	0.273190	-3.006672
H	0.275372	1.330931	-3.286933
C	-2.016564	-4.622553	-0.288505
H	-1.210202	-5.255320	0.086055
C	-4.058415	-2.995107	-1.304833
C	-2.947384	4.207163	2.498671
H	-2.945609	4.633598	3.503462
C	-1.031096	-0.446500	-3.481568
H	-1.777001	0.031887	-4.126571
C	-2.811414	-5.077028	-1.337053
H	-2.629006	-6.061758	-1.775151
C	3.454763	-4.406809	-1.393271
H	3.590748	-4.357031	-2.484604
H	2.373890	-4.445386	-1.192020
H	3.904016	-5.354901	-1.054370
C	-1.379698	-2.887925	1.454361
H	-1.205388	-1.814040	1.296809
C	-4.309270	2.313923	-1.999862
H	-4.881047	1.682393	-1.304134
H	-4.293070	1.807724	-2.978440
H	-4.847023	3.269909	-2.116451
C	3.710144	-4.258870	1.542105
H	3.847878	-5.238852	1.080276
C	2.971421	-0.423425	3.371349
H	3.338565	0.353874	2.685810
C	1.409203	-0.565963	-3.309735
H	2.279710	-0.179493	-3.844694
C	-4.785192	-1.758212	-3.382182
H	-3.818504	-1.238963	-3.438014
H	-5.558184	-1.095493	-3.804516
H	-4.726586	-2.655221	-4.020093
C	1.314267	-1.846919	-2.886973
H	2.095265	-2.594067	-3.053423
C	-2.074294	3.359830	-2.504105
H	-2.537263	4.336777	-2.724832
H	-2.006917	2.806788	-3.453068
H	-1.047163	3.532387	-2.149773
C	3.348651	2.148817	-4.579381
H	3.407332	3.124095	-5.089597
H	2.299216	1.823885	-4.588524
H	3.933361	1.434517	-5.180231
C	5.308842	2.830078	-3.185472
H	5.971172	2.250807	-3.849350
H	5.769526	2.853866	-2.188668
H	5.274623	3.866208	-3.561235
C	-6.512333	-2.730189	-1.815447
H	-6.578653	-3.681549	-2.368415
H	-7.278687	-2.052580	-2.225563
H	-6.769876	-2.940826	-0.765778
C	5.604271	-3.158404	-0.994058
H	6.106470	-4.022033	-0.526527
H	6.058200	-2.241357	-0.592299
H	5.809656	-3.188839	-2.076397
B	-2.641933	-0.103676	0.282043
B	2.667511	0.129937	0.036827
C	-2.144801	-3.037470	2.771020
H	-2.346458	-4.099663	2.988535
H	-3.106975	-2.505147	2.736270
H	-1.557256	-2.623063	3.606477
C	-0.007351	-3.543221	1.533917
H	0.600157	-3.060238	2.312417
H	0.541025	-3.437852	0.586566
H	-0.066127	-4.614851	1.786971
C	1.453982	-0.257407	3.482436
H	1.021436	-1.020052	4.151561
H	0.978639	-0.353755	2.495228
H	1.197935	0.732754	3.891256
C	3.662681	-0.207860	4.714318
H	4.752803	-0.337286	4.630103
H	3.298012	-0.906923	5.484580
H	3.467824	0.811819	5.082941
C	-2.044549	1.108607	3.732504
H	-1.237693	1.767964	4.087143
H	-1.700439	0.632966	2.803383
H	-2.195044	0.318250	4.485225
C	2.930617	3.210720	2.233243
H	3.066949	4.304901	2.251478
H	3.848702	2.756825	1.832977
H	2.811015	2.867880	3.274114
C	0.433846	3.379046	2.042829
H	0.451428	4.475500	2.151646
H	0.320376	2.959810	3.053059
H	-0.460897	3.106676	1.466514
C	-3.764870	2.598323	4.801465
H	-3.955098	1.850565	5.587094
H	-4.684854	3.187463	4.661952
H	-2.984301	3.275716	5.184383

Anthracene activation:
Compound 1: 1,4-activation

158
scf done: -3823.88188842

C	2.471051	1.290485	3.864193
C	2.728033	0.344927	2.846265
C	2.289224	-0.995367	2.984519
C	1.506103	-1.331923	4.090667
C	1.208688	-0.395312	5.076006
C	1.713070	0.893496	4.971969
N	3.409940	0.707114	1.661481
C	4.743099	1.127261	1.613626
C	5.136617	1.256590	0.325790
N	4.077424	0.935918	-0.525110
C	4.248900	0.796953	-1.924073
C	4.560543	-0.472025	-2.453634
C	4.740720	-0.593643	-3.834418
C	4.625730	0.509441	-4.672915
C	4.317457	1.755219	-4.138253
C	4.120010	1.919997	-2.764274
C	4.650819	-1.692067	-1.561713
C	5.910180	-2.516681	-1.813255
C	3.796917	3.284159	-2.191701
C	5.057233	4.141890	-2.072073
C	2.746775	-2.062325	2.006628

C	1.933628	-3.343831	2.069946
C	3.024960	2.703190	3.821647
C	4.276450	2.822703	4.696511
B	2.932244	0.556316	0.284157
O	1.741481	0.124494	-0.154138
Al	0.040127	0.478089	0.029848
C	-0.367723	1.725944	1.660340
C	-1.735948	2.123208	1.256085
C	-1.838792	2.446851	-0.148569
C	-0.565244	2.269981	-0.884771
C	0.537500	2.978879	-0.185023
C	0.645361	2.688339	1.141397
C	-2.850564	2.243945	2.066781
C	-4.102647	2.675889	1.543147
C	-4.189833	3.034436	0.157738
C	-3.033857	2.910460	-0.662607
C	-5.433227	3.484480	-0.355641
C	-6.546216	3.569019	0.451307
C	-6.463199	3.206322	1.814613
C	-5.268429	2.772571	2.345691
O	-0.896834	-0.965990	-0.191967
B	-1.755416	-1.823604	-0.751088
N	-2.503974	-1.681949	-1.996813
C	-2.657125	-0.517977	-2.782711
C	-1.533618	-0.014811	-3.474454
C	-1.687255	1.144678	-4.239797
C	-2.918961	1.781592	-4.335945
C	-4.019372	1.269675	-3.656182
C	-3.917769	0.124599	-2.859393
C	-0.209067	-0.757531	-3.441706
C	0.996242	0.111084	-3.774872
C	-5.129418	-0.401972	-2.099033
C	-6.443099	0.262851	-2.494324
C	-3.275750	-2.831601	-2.173798
C	-3.080792	-3.676795	-1.136781
N	-2.168084	-3.120683	-0.237402
C	-1.688289	-3.827429	0.889031
C	-1.947205	-3.329881	2.182689
C	-1.493353	-4.068281	3.280161
C	-0.814647	-5.268916	3.107139
C	-0.552469	-5.739598	1.824700
C	-0.963797	-5.025875	0.696187
C	-2.769031	-2.070342	2.376459
C	-2.405333	-1.288260	3.632519
C	-0.574655	-5.472764	-0.702804
C	-0.264222	-6.960146	-0.815926
C	-0.264156	-1.987774	-4.350719
C	-4.947942	-0.336299	-0.581736
C	0.594102	-4.633324	-1.222380
C	-4.259807	-2.411810	2.346267
C	3.385597	-2.540041	-1.691310
C	2.710586	4.007655	-2.984288
C	4.234942	-2.359439	2.215503
C	2.005518	3.762746	4.247318
H	1.289862	3.555115	-0.728280
H	1.503735	2.996504	1.738091
H	-3.530059	-4.652193	-0.963021
H	6.112922	1.539652	-0.061862
H	5.331589	1.277823	2.514264
H	-3.897189	-2.975789	-3.054316
H	-2.556776	-1.409042	1.525073
H	-1.427540	-5.269269	-1.367095
H	0.596023	-0.679280	5.935078
H	-0.640277	2.425603	-1.964946
H	3.322655	2.908775	2.780656

H	-4.979223	1.780458	-3.741735
H	4.973494	-1.572488	-4.260734
H	4.771537	0.396615	-5.750012
H	1.505735	1.615229	5.765596
H	-0.476788	-5.837310	3.977240
H	-1.686859	-3.702076	4.290210
H	2.616321	-1.660282	0.992945
H	-5.228288	-1.468622	-2.353393
H	1.129679	-2.350806	4.189601
H	3.417145	3.108022	-1.173373
H	-0.247697	1.413402	2.703179
H	-0.829751	1.546538	-4.782017
H	0.001316	-6.671607	1.700990
H	-3.025330	2.679419	-4.950533
H	2.418759	4.941268	-2.476397
H	1.812705	3.381782	-3.098361
H	3.053841	4.286047	-3.993104
H	-0.064893	-1.123324	-2.414793
H	-4.531619	-2.919421	1.409200
H	-4.871490	-1.499100	2.425917
H	-4.523283	-3.078352	3.183670
H	4.223694	2.616937	-4.803509
H	4.700105	-1.330696	-0.524248
H	-4.056868	-0.883985	-0.252371
H	-5.817932	-0.783182	-0.077275
H	-4.855746	0.702540	-0.240949
H	-2.656432	-1.835316	4.555730
H	-2.970535	-0.344356	3.655443
H	-1.332776	-1.043767	3.657777
H	1.802316	3.717953	5.328437
H	1.044826	3.634803	3.726285
H	2.391356	4.772628	4.034211
H	4.719637	3.828482	4.618748
H	5.044823	2.088913	4.416593
H	4.025303	2.642223	5.753944
H	-6.472772	1.321429	-2.190851
H	-7.278540	-0.239906	-1.984235
H	-6.629058	0.208073	-3.577853
H	5.508024	4.320707	-3.061774
H	5.813831	3.651273	-1.442809
H	4.826696	5.121524	-1.622767
H	-0.408351	-1.689592	-5.401803
H	-1.087670	-2.659049	-4.067383
H	0.676953	-2.556414	-4.282666
H	1.926226	-0.450849	-3.612632
H	1.045904	1.003234	-3.134046
H	0.997153	0.440006	-4.826333
H	3.272600	-2.931738	-2.715704
H	2.490886	-1.949233	-1.448429
H	3.423460	-3.400808	-1.006413
H	6.817845	-1.900590	-1.724527
H	5.910558	-2.976035	-2.814684
H	5.981228	-3.335335	-1.080067
H	1.511669	-4.857650	-0.658087
H	0.397048	-3.556696	-1.122752
H	0.788742	-4.847017	-2.285126
H	4.410290	-2.767398	3.224191
H	4.856812	-1.460670	2.096649
H	4.581632	-3.107422	1.484570
H	2.047778	-3.867040	3.032388
H	2.273205	-4.037840	1.288334
H	0.863164	-3.158868	1.905857
H	-0.104114	-7.231563	-1.870573
H	-1.085584	-7.581017	-0.426273
H	0.654240	-7.232591	-0.271608

H	-2.779657	1.990515	3.128808
H	-3.109336	3.144490	-1.727750
H	-5.204567	2.484122	3.398673
H	-7.352482	3.265789	2.446526
H	-7.498634	3.907826	0.036908
H	-5.494356	3.747757	-1.415146
K	-1.329600	4.962510	1.239613

Compound 1: 9,10-activation

158

scf done: -3823.90779528

K	-0.000793	5.898809	0.000546
C	-1.340684	3.269553	-0.405797
C	-2.535171	3.861149	-0.829180
H	-2.805483	3.825193	-1.888097
C	-3.390225	4.462885	0.103014
H	-4.328398	4.911367	-0.233248
C	-3.064544	4.462573	1.462092
H	-3.746221	4.913338	2.187879
C	-1.876065	3.865403	1.900349
H	-1.626854	3.848009	2.965447
C	-1.005091	3.272466	0.979123
H	-5.162614	2.471459	-3.455974
C	-4.129510	0.840172	-2.498704
C	-2.948681	0.065637	-2.486831
C	2.534268	3.861845	0.829254
H	2.804711	3.825873	1.888142
C	1.339902	3.269951	0.405931
C	-0.305396	2.629442	-1.263326
H	0.576210	2.559786	2.320936
C	0.304794	2.629641	1.263490
C	-3.225662	2.145103	-4.335331
H	-3.332888	2.956186	-5.060473
H	-0.576790	2.559711	-2.320788
H	4.327195	4.912521	0.233253
H	1.625763	3.848534	-2.965326
C	3.389116	4.463821	-0.102976
C	3.063349	4.463493	-1.462030
C	1.875017	3.865990	-1.900238
H	3.744859	4.914465	-2.187844
H	-1.276178	1.573268	-5.041238
C	-2.070718	1.369992	-4.320343
C	3.225442	2.145392	4.335336
C	-1.915205	0.318458	-3.412151
C	4.248836	1.871731	3.435748
H	5.162375	2.472042	3.456025
Al	-0.000081	1.004124	0.000086
C	2.070613	1.370113	4.320308
O	-1.413675	0.031839	0.302288
H	1.275990	1.573300	5.041140
C	1.915315	0.318518	3.412140
C	0.715359	-0.598215	3.503883
H	0.555227	-1.038270	2.507521
C	1.043948	-1.737132	4.472863
H	1.257540	-1.334407	5.476303
H	0.195819	-2.432009	4.552961
H	1.926429	-2.304199	4.141838
C	-0.576744	0.097575	3.908682
H	-0.806052	0.944579	3.247566
H	-0.542142	0.465086	4.947161
H	-1.414448	-0.608512	3.841615
N	-2.816657	-0.982226	-1.538335
N	-2.770706	-2.066051	0.490796
C	-3.612386	-2.131178	-1.595341
B	-2.233990	-0.900717	-0.198703
H	-4.127725	-2.419581	-2.509233
C	-3.584776	-2.768426	-0.402475
C	-2.792158	-2.344624	1.881256
H	-4.075866	-3.695339	-0.113749
C	-3.671739	-1.616285	2.712878
C	-3.716649	-1.925468	4.075168
H	-4.385538	-1.361841	4.730087
C	-2.928377	-2.938570	4.609238
H	-2.980661	-3.171223	5.675664
C	-2.072147	-3.652261	3.779702
H	-1.457961	-4.452971	4.199634
C	-1.982897	-3.368150	2.412989
C	-1.070171	-4.193665	1.531410
H	-1.068157	-3.711476	0.544035
H	0.821113	-3.222004	2.031047
C	0.370044	-4.223843	2.037085
H	0.991541	-4.866813	1.397192
H	0.440245	-4.621175	3.061676
C	-1.630004	-5.606915	1.361992
H	-2.650800	-5.587871	0.951457
H	-1.668627	-6.138557	2.326565
H	-1.000294	-6.199299	0.679625
C	1.071020	-4.193572	-1.531533
C	-0.369145	-4.224236	-2.037350
H	-0.820655	-3.222591	-2.031074
H	-0.439066	-4.621285	-3.062065
H	-0.990404	-4.867679	-1.397699
C	1.631269	-5.606640	-1.361993
H	1.669971	-6.138386	-2.326505
H	2.652090	-5.587260	-0.951535
H	1.001773	-6.199115	-0.679510
C	1.983531	-3.367801	-2.413090
H	4.076647	-3.694663	0.113274
H	4.128478	-2.419235	2.508984
C	2.792472	-2.344024	-1.881358
C	3.585344	-2.767925	0.402219
N	2.771087	-2.065540	-0.490887
B	2.234267	-0.900417	0.198850
N	2.817030	-0.982032	1.538387
H	-0.195094	-2.431588	-4.553234
H	-1.925726	-2.304301	-4.142027
H	-1.257147	-1.334193	-5.476430
C	2.948862	0.065830	2.486901
O	1.413627	0.032018	-0.301818
C	4.129553	0.840575	2.498757
C	0.576894	0.097991	-3.908724
H	0.542192	0.465628	-4.947158
H	1.414734	-0.607944	-3.841779
H	0.806073	0.944963	-3.247515
C	2.072854	-3.651847	-3.779818
H	1.458926	-4.452749	-4.199761
C	2.928813	-2.937846	-4.609349
H	2.981128	-3.170461	-5.675782
C	3.671803	-1.615375	-2.712968
C	3.716780	-1.924503	-4.075264
H	6.215306	-0.921342	2.847709
C	6.483046	-0.007581	2.297003
H	7.292521	-0.259720	1.593741
H	6.882566	0.717054	3.025139
H	4.612160	1.676596	-2.158054
C	5.661637	1.789276	0.727638
C	5.277913	0.562252	1.549022
H	4.797947	2.176671	0.170755
H	6.047764	2.603580	1.362322
C	-5.662313	1.788532	-0.727950

H	-4.798907	2.176426	-0.170967
H	-6.048726	2.602553	-1.362824
H	-6.452606	1.533296	-0.004503
C	-5.277876	0.561555	-1.549062
H	-4.939382	-0.211022	-0.845669
C	-6.482658	-0.008923	-2.297139
H	-6.882454	0.715517	-3.025322
H	-7.292070	-0.261451	-1.593944
C	-4.541596	-0.501923	2.168291
C	1.004259	3.272805	-0.978983
C	-1.043423	-1.736964	-4.473040
H	-0.554876	-1.038171	-2.507633
C	-4.248975	1.871304	-3.435703
C	-0.715088	-0.598066	-3.503958
H	3.332498	2.956528	5.060443
H	4.939666	-0.210603	0.845813
H	-6.214416	-0.922555	-2.847805
H	4.385460	-1.360616	-4.730182
H	6.451920	1.534296	0.004087
H	1.068769	-3.711315	-0.544187
H	2.968014	1.001387	-2.109352
C	3.970008	0.867988	-2.539485
C	4.541479	-0.500845	-2.168402
H	4.514407	-0.581337	-1.072562
C	6.000987	-0.639806	-2.594531
C	3.612986	-2.130842	1.595181
C	-6.000981	-0.640531	2.594920
C	-3.969874	0.866978	2.538791
H	-4.514884	-0.582726	1.072467
H	-4.612063	1.675537	2.157352
H	-3.894561	0.981722	3.632590
H	-2.968013	1.000161	2.108318
H	-6.617874	0.123104	2.095709
H	-6.403258	-1.630409	2.331223
H	-6.129474	-0.502833	3.680428
H	6.617858	0.123922	-2.095435
H	6.403001	-1.629658	-2.330340
H	6.129823	-0.502572	-3.680057
H	3.894983	0.982411	-3.633337

Anion of 2: 1,4-activation

157
scf done: -3224.18714756

H	1.651640	6.541567	1.216334
C	1.949488	5.490573	1.177180
C	2.955321	5.080571	0.310787
C	3.340590	3.739229	0.239255
C	4.290577	0.994479	1.559869
C	4.421980	3.326722	-0.738196
H	4.569291	2.244607	-0.622625
C	3.988638	3.581449	-2.180732
H	3.802877	4.652278	-2.363788
H	4.770926	3.256145	-2.884796
H	3.061665	3.035964	-2.410742
C	5.756670	3.996445	-0.416798
H	6.074129	3.769367	0.612387
H	5.695878	5.092621	-0.515973
H	6.544069	3.642054	-1.101362
C	1.666717	3.202905	1.943984
C	2.693521	2.799318	1.068640
C	1.313758	4.554857	1.984522
H	0.514957	4.876822	2.655614
C	1.709480	2.184392	4.212258
H	1.670077	3.178453	4.687809
H	1.233233	1.459966	4.889747
H	2.768366	1.906864	4.097931
C	0.989474	2.211023	2.862671
H	1.096380	1.218124	2.399916
C	-0.500868	2.467313	3.040551
H	-0.954825	1.647579	3.611444
H	-0.698922	3.400392	3.593387
H	-1.012104	2.521580	2.070023
H	-0.556564	3.631275	0.102469
C	-0.653640	2.975286	-0.767787
C	0.259439	3.297968	-1.908137
H	0.952054	4.142054	-1.856987
N	3.088787	1.436904	1.003775
C	4.634788	-0.199022	1.021298
H	5.490408	-0.830194	1.254408
N	3.677877	-0.589125	0.078512
B	2.634938	0.440076	0.029196
H	2.823165	-2.341435	1.422831
C	4.119322	-3.986615	1.860196
H	4.432814	-4.957665	1.442920
H	3.707739	-4.173165	2.864703
H	5.020657	-3.364840	1.972641
C	0.182250	2.440660	-2.951960
H	0.808089	2.517060	-3.846635
C	3.083760	-3.305727	0.964936
C	1.803638	-4.132178	0.857359
C	-0.808504	1.331017	-2.772284
H	1.969522	-5.078657	0.318861
H	1.418099	-4.387053	1.855062
Al	-0.131802	0.968962	-0.848412
H	3.044658	-1.954793	3.649164
C	2.168425	-1.331407	3.879874
H	2.298944	-0.952940	4.906027
H	2.189784	-0.471612	3.196439
H	-3.144011	3.957798	0.172948
H	0.775543	-2.402137	2.664809
C	-3.202208	3.289092	-0.691724
C	0.879846	-2.135314	3.725485
C	-2.047707	2.779594	-1.247215
C	-2.132358	1.875997	-2.368687
C	-3.361558	1.549480	-2.897602
H	-3.425457	0.854529	-3.739322
C	0.170551	-2.581409	-2.435954
H	0.414889	-2.200624	-1.432500
H	0.921653	-0.793428	-3.422715
H	2.137977	-2.050536	-3.127316
C	1.094475	-1.878422	-3.420124
H	0.975169	-2.259672	-4.447747
C	0.419963	-4.091962	-2.440115
H	-0.185986	-4.600041	-1.674988
H	0.153873	-4.520181	-3.420753
H	1.480285	-4.310137	-2.243781
C	-1.800244	-2.194551	-3.972721
C	-1.302648	-2.329201	-2.673955
H	-1.102785	-2.182144	-4.812508
C	-3.165360	-2.081743	-4.213537
H	-3.534273	-1.973047	-5.236942
C	-4.056226	-2.100716	-3.148073
H	-5.128680	-2.001689	-3.335691
C	-3.601822	-2.216248	-1.831304
C	-2.214487	-2.330603	-1.598982
C	-4.558878	2.072728	-2.343836
C	-5.837359	1.749448	-2.863570
C	-2.010110	-3.547888	0.534552
H	-4.034665	-2.228900	0.242717
C	-4.605389	-2.221821	-0.695492

H	-2.302666	-4.502313	0.100612
C	-6.991477	2.240019	-2.291301
C	-5.477039	-0.970479	-0.694982
H	-6.132415	-0.956701	0.190281
H	-4.865684	-0.058241	-0.686791
H	-6.124775	-0.916333	-1.583485
C	-5.451624	-3.494532	-0.711417
H	-4.821319	-4.395087	-0.655482
H	-6.053361	-3.562881	-1.632696
H	-6.145244	-3.509989	0.144877
H	-1.925575	-3.873087	2.713191
H	-7.830608	3.467231	-0.703306
C	-1.820791	-3.235386	1.837629
C	-6.914293	3.084438	-1.161104
C	-5.684929	3.426457	-0.638498
H	0.026010	-4.036591	4.381286
H	-5.620619	4.081578	0.235722
N	-1.419919	-1.900129	1.952611
C	-4.479111	2.944819	-1.208275
B	-1.341526	-1.325125	0.606001
O	-1.008758	-0.084386	0.267338
H	-0.859223	0.597628	-3.583697
H	3.445912	5.814175	-0.334919
C	0.932997	-3.432377	4.534429
C	-0.443630	-0.732305	5.345548
H	0.398698	-0.813639	6.037758
C	-0.355962	-1.340236	4.088916
H	1.801805	-4.043457	4.242249
C	-1.581064	-0.031489	5.727375
H	-1.630779	0.443119	6.710785
C	-2.651152	0.071042	4.845908
H	-3.540319	0.633784	5.140625
C	-2.611417	-0.527671	3.583313
C	-1.450500	-1.239987	3.205702
H	4.808644	1.570333	2.324639
C	3.917337	-1.669227	-0.805915
C	3.673168	-2.993548	-0.393341
C	4.006502	-4.043120	-1.257030
H	3.826031	-5.073239	-0.938396
C	4.443376	-1.407878	-2.092333
C	4.555022	-3.797954	-2.508395
C	4.761839	-2.485899	-2.921390
H	4.808705	-4.629273	-3.171161
H	5.171945	-2.293970	-3.915041
H	-7.967218	1.972910	-2.706411
H	-5.889634	1.084389	-3.731023
H	1.017815	-3.224808	5.613842
O	1.559110	0.465247	-0.747031
H	3.993943	0.654419	-2.007177
C	4.694049	0.011074	-2.558789
C	4.405964	0.219562	-4.041955
C	6.117551	0.442258	-2.200770
C	-3.791851	-0.374743	2.647375
H	1.012658	-3.579369	0.331940
N	-1.740242	-2.436514	-0.265202
H	4.469529	1.289858	-4.289440
H	5.128141	-0.307022	-4.687064
H	3.394874	-0.126069	-4.302096
H	6.307096	1.478684	-2.521765
H	6.287451	0.387696	-1.115405
H	6.856326	-0.208279	-2.697530
C	-3.821204	1.024608	2.032252
H	-3.643783	-1.090406	1.826740
C	-5.116395	-0.727458	3.320253
H	-5.933286	-0.702358	2.582532
H	-5.086024	-1.734295	3.765091
H	-5.377856	-0.012991	4.117646
H	-4.692036	1.147029	1.371025
H	-3.877258	1.799837	2.814430
H	-2.920437	1.205168	1.430220

Anion of 2: 9,10-activation

157

scf done: -3224.19265160

C	1.032856	-0.935745	-3.166076
C	-0.257197	-1.271214	-2.493025
C	-1.319181	-0.454190	-3.161643
C	-1.033512	0.937795	-3.165320
C	0.256592	1.272767	-2.492121
C	1.318522	0.456213	-3.161404
Al	-0.000062	0.000203	-0.880761
O	1.445095	-0.184742	0.127888
B	2.601428	0.178265	0.684998
N	3.283684	1.486031	0.676236
C	4.387182	1.414968	1.526005
C	4.502128	0.169519	2.032207
N	3.474881	-0.638179	1.538571
C	3.475239	-2.020375	1.833568
C	3.226928	-2.438174	3.158911
C	3.296751	-3.801992	3.456535
C	3.599666	-4.731091	2.468061
C	3.831693	-4.306892	1.164575
C	3.777427	-2.953131	0.818477
C	2.821134	-1.421231	4.207261
C	3.052280	-1.879764	5.641901
C	4.033015	-2.494365	-0.605888
C	5.377764	-1.773785	-0.736479
O	-1.444676	0.184540	0.128812
B	-2.601243	-0.178745	0.685300
N	-3.474723	0.637287	1.539238
C	-4.502029	-0.170633	2.032371
C	-4.387144	-1.415791	1.525441
N	-3.283644	-1.486410	0.675615
C	-3.095268	-2.596264	-0.181443
C	-1.903102	-3.344744	-0.083357
C	-1.705785	-4.410395	-0.963641
C	-2.674660	-4.760612	-1.898696
C	-3.866606	-4.050306	-1.950374
C	-4.101323	-2.960499	-1.105316
C	-0.910592	-3.050195	1.022381
C	0.486141	-3.581915	0.755146
C	-5.442578	-2.250473	-1.179337
C	-6.549852	-3.136819	-0.604089
C	3.095241	2.596304	-0.180258
C	1.903029	3.344659	-0.081805
C	1.705670	4.410777	-0.961517
C	2.674545	4.761559	-1.896361
C	3.866517	4.051320	-1.948419
C	4.101262	2.961066	-1.103951
C	0.910494	3.049498	1.023749
C	-0.486319	3.581095	0.756694
C	5.442506	2.251066	-1.178362
C	6.549768	3.137040	-0.602496
C	-3.475002	2.019303	1.835068
C	-3.226405	2.436281	3.160606
C	-3.296122	3.799915	3.459096
C	-3.599235	4.729628	2.471260
C	-3.831517	4.306241	1.167555
C	-3.777321	2.952695	0.820597

C	-2.820381	1.418661	4.208208
C	-3.051147	1.876295	5.643190
C	-4.033064	2.494825	-0.604034
C	-3.949535	3.622258	-1.624490
C	-5.377755	1.774204	-0.734995
C	-1.367830	0.995244	4.000084
C	5.807206	1.787015	-2.584062
C	1.447152	3.583483	2.355144
C	-5.807214	-1.785547	-2.584770
C	3.949326	-3.621140	-1.627063
C	-1.447349	-3.584648	2.353545
C	1.368518	-0.997712	3.999838
H	-5.260773	0.222902	2.705582
H	5.260915	-0.224377	2.705158
H	5.011333	2.283901	1.718787
H	-5.011342	-2.284807	1.717685
H	-3.238845	1.771805	-0.856713
H	-3.436497	0.520000	4.050000
H	2.500745	5.596449	-2.580113
H	-0.483245	-2.342189	-2.443045
H	5.375972	1.349063	-0.555160
C	-2.475219	-0.919650	-3.786511
H	-4.634088	-4.336734	-2.674404
H	3.102682	-4.144641	4.474476
H	3.645998	-5.795502	2.712737
H	4.633989	4.338157	-2.672298
H	-3.645495	5.793889	2.716605
H	-4.055091	5.048072	0.400126
H	0.830336	1.956309	1.118416
H	-5.376092	-1.348847	-0.555585
H	0.777584	4.981744	-0.909824
H	3.238822	-1.771134	-0.858034
H	0.482662	2.343705	-2.441437
H	-0.777738	-4.981449	-0.912244
H	-3.101813	4.141918	4.477209
C	-1.910812	1.823671	-3.789012
H	-2.500882	-5.595132	-2.582905
H	4.050252	-3.204624	-2.637746
H	2.983659	-4.146930	-1.576984
H	4.754608	-4.361520	-1.487124
H	-0.830280	-1.957055	1.117489
H	-5.466347	0.936862	-0.029074
H	-5.492252	1.373567	-1.754268
H	-6.209984	2.471541	-0.541853
H	4.055124	-5.048244	0.396641
H	3.437184	-0.522452	4.049491
C	2.474616	0.922147	-3.786017
H	-5.048192	-1.098039	-2.976968
H	-6.773686	-1.256264	-2.568526
H	-5.904807	-2.627367	-3.289739
C	1.910149	-1.821178	-3.790482
H	-4.754806	4.362536	-1.483957
H	-4.050612	3.206397	-2.635427

H	-2.983865	4.148025	-1.574211
H	5.904844	2.629272	-3.288504
H	5.048186	1.099794	-2.976774
H	6.773666	1.257702	-2.568117
H	7.510688	2.597678	-0.580971
H	6.320969	3.467967	0.421607
H	6.685794	4.043362	-1.215220
H	-6.685811	-4.042772	-1.217374
H	-7.510789	-2.597496	-0.582321
H	-6.321146	-3.468374	0.419833
H	6.209930	-2.471312	-0.543749
H	5.466462	-0.936857	-0.030080
H	5.492287	-1.372573	-1.755522
H	-1.567320	-4.679691	2.310006
H	-2.422185	-3.140818	2.602909
H	-0.746219	-3.352485	3.170604
H	1.164211	-3.261142	1.554874
H	0.889552	-3.199057	-0.193672
H	0.516273	-4.683215	0.730125
H	0.683984	-1.838835	4.195395
H	1.189160	-0.652671	2.971803
H	1.106513	-0.178229	4.684843
H	4.089837	-2.210801	5.805155
H	2.383910	-2.710451	5.921102
H	2.844768	-1.052960	6.338787
H	-0.683220	1.836213	4.196023
H	-1.188789	0.650923	2.971751
H	-1.105644	0.175283	4.684447
H	1.567058	4.678549	2.312025
H	2.422012	3.139614	2.604349
H	0.746015	3.350971	3.172100
H	-0.516664	4.682398	0.732142
H	-1.164319	3.259873	1.556302
H	-0.889651	3.198540	-0.192283
H	-2.843559	1.049032	6.339508
H	-4.088632	2.207339	5.806885
H	-2.382627	2.706737	5.922770
C	-3.062309	1.345071	-4.420088
C	-3.338995	-0.021998	-4.423344
C	3.338316	0.024953	-4.423541
C	3.061599	-1.342133	-4.421264
H	1.679289	-2.890396	-3.797633
H	3.737117	-2.037766	-4.928002
H	4.228414	0.398090	-4.937409
H	2.692871	1.993757	-3.780752
H	-1.679952	2.892894	-3.795389
H	-3.737884	2.041082	-4.926229
H	-4.229136	-0.394760	-4.937411
H	-2.693486	-1.991264	-3.782058

Truncated 1: 1,4-activation

86

scf done: -2878.788685

C	5.143719	2.336544	-2.087019
C	4.424869	1.697169	-1.069718
C	3.685311	2.475372	-0.168714
C	3.660362	3.860097	-0.291255

C	4.378983	4.495508	-1.303513
C	5.121890	3.724734	-2.196288
N	4.421591	0.299565	-0.949570
C	5.502367	-0.523510	-1.275170
C	5.185971	-1.820139	-1.056279
N	3.857519	-1.907492	-0.626868
C	3.238492	-3.052912	-0.125542
C	3.937717	-3.981783	0.662744
C	3.274004	-5.071787	1.224001
C	1.904184	-5.251366	1.025466
C	1.205167	-4.340995	0.228691
C	1.865063	-3.259823	-0.352764
H	5.003265	-3.829935	0.849066
H	1.326683	-2.572403	-1.008205
H	3.116192	1.978496	0.617187
H	5.698327	1.740802	-2.814637
B	3.326651	-0.550913	-0.525626
O	2.123644	-0.227798	-0.020187
K	1.379260	-1.326471	2.288097
Al	0.530018	0.326457	-0.590839
C	0.176366	2.285154	-1.032833
C	-1.257274	1.998024	-1.335498
C	-1.439704	0.831393	-2.163078
C	-0.153564	0.141469	-2.499860
C	0.865194	1.112649	-3.018242
C	1.033213	2.220308	-2.260975
C	-2.353175	2.631637	-0.788485
C	-3.667872	2.156889	-1.030394
C	-3.850909	1.025151	-1.887620
C	-2.712995	0.387292	-2.446308
C	-5.167215	0.551141	-2.117566
C	-6.255780	1.154172	-1.526246
C	-6.074221	2.269135	-0.676731
C	-4.808542	2.756823	-0.439126
O	-0.453899	-0.429950	0.713191
B	-1.709446	-0.187271	1.144346
N	-2.968151	-0.822892	0.776311
C	-3.199573	-1.834207	-0.163850
C	-2.118505	-2.473067	-0.794528
C	-2.332256	-3.472903	-1.736118
C	-3.625692	-3.873004	-2.071986
C	-4.701127	-3.249182	-1.444164
C	-4.497312	-2.243214	-0.502793
H	-1.107748	-2.153685	-0.550722
H	-5.363480	-1.756283	-0.057119
C	-3.960649	-0.281744	1.599921
C	-3.421501	0.579263	2.488553
N	-2.046859	0.689724	2.261884
C	-1.133070	1.254125	3.149337
C	0.037922	1.865566	2.663228
C	1.004149	2.342497	3.546952
C	0.816111	2.250011	4.927887
C	-0.361351	1.680256	5.413939
C	-1.324296	1.180358	4.539721
H	0.164969	1.990940	1.585975
H	-2.227626	0.705707	4.929094
H	1.435781	0.916653	-3.930133
H	1.759200	3.003939	-2.491382

H	-3.935946	1.194448	3.223551
H	5.798900	-2.703461	-1.221795
H	6.444498	-0.116400	-1.634537
H	-5.014049	-0.506806	1.469069
H	4.356896	5.583134	-1.398552
H	-0.275741	-0.779698	-3.079599
H	-5.724569	-3.540152	-1.694172
H	3.835468	-5.784339	1.833593
H	1.388284	-6.104788	1.470633
H	5.682256	4.207312	-3.000755
H	1.569146	2.637967	5.617215
H	1.902984	2.820138	3.146367
H	3.068724	4.449950	0.413441
H	0.339249	3.161060	-0.394781
H	-1.470209	-3.940111	-2.220360
H	-0.530746	1.612444	6.491645
H	-3.792406	-4.656512	-2.814092
H	0.137021	-4.478805	0.039010
H	-2.214812	3.498796	-0.135860
H	-2.858258	-0.501821	-3.066037
H	-4.662865	3.618904	0.217900
H	-6.941388	2.745049	-0.211756
H	-7.263061	0.774871	-1.717260
H	-5.300603	-0.314435	-2.771521

Truncated 1: 9,10-activation

86
scf done: -2878.797855

C	0.050503	3.201306	3.035186
C	0.104007	1.807761	2.881212
C	-1.098030	1.101643	2.707409
C	-2.311300	1.779001	2.647101
C	-2.357558	3.166867	2.783852
C	-1.171867	3.869211	2.991917
N	1.325845	1.130179	2.854505
C	2.493079	1.584403	3.466216
C	3.551425	0.860425	3.040653
N	3.139623	-0.097944	2.110295
C	4.012895	-0.876460	1.350056
C	5.392428	-0.617599	1.315953
C	6.239796	-1.385213	0.520784
C	5.739597	-2.422310	-0.264172
C	4.369271	-2.685271	-0.231566
C	3.515617	-1.931481	0.565348
B	1.689514	0.036983	1.955680
O	0.874556	-0.634866	1.135823
Al	-0.090690	-0.176823	-0.255966
C	-0.106142	1.863535	-0.635261
C	-0.614160	1.765641	-2.028019
C	-0.008614	0.716483	-2.781225
C	1.009872	-0.046790	-2.011509
C	1.993281	0.901496	-1.414765
C	1.381236	1.943335	-0.656686
C	-1.576727	2.582030	-2.633465
C	-1.955883	2.362983	-3.963493
C	-1.368942	1.330226	-4.698216

C	-0.396384	0.511790	-4.110243
C	3.388707	0.838443	-1.500614
C	4.176367	1.791027	-0.845201
C	3.577340	2.805637	-0.094935
C	2.183775	2.883216	0.000736
O	-1.565797	-1.064172	-0.289835
B	-2.579632	-1.883925	-0.028130
N	-3.976024	-1.519862	0.206081
C	-4.691960	-2.696526	0.441411
C	-3.868033	-3.762775	0.344838
N	-2.562684	-3.337987	0.088033
C	-1.483582	-4.209710	-0.096183
C	-1.658465	-5.448726	-0.730019
C	-0.581323	-6.314892	-0.898476
C	0.689768	-5.959795	-0.450210
C	0.868261	-4.727033	0.177993
C	-0.203932	-3.858143	0.360758
C	-4.505582	-0.237594	0.356955
C	-3.992113	0.831783	-0.394966
C	-4.497632	2.115049	-0.223263
C	-5.526879	2.366182	0.684790
C	-6.045763	1.306359	1.426567
C	-5.540542	0.017626	1.270746
H	-2.644814	-5.722775	-1.110135
H	-0.058264	-2.912846	0.886406
H	-3.193616	0.642816	-1.112288
H	-5.931064	-0.797079	1.883373
H	5.808719	0.209139	1.892273
H	2.445896	-2.132241	0.576626
H	-1.068843	0.015102	2.618780
H	0.977249	3.764391	3.164051
H	0.054112	-0.304629	-4.682378
H	-1.678772	1.149710	-5.730583
H	-2.723836	2.991832	-4.420796
H	-2.040634	3.384066	-2.051993
H	-0.738879	-7.275608	-1.395542
H	1.710205	3.657275	0.610499
H	-0.606976	2.600339	0.002380
H	1.533537	-6.640913	-0.581888
H	1.450234	-0.902138	-2.536062
H	4.197817	3.525971	0.443426
H	3.862373	0.017164	-2.045357
H	5.264735	1.713299	-0.892517
H	1.854142	-4.442976	0.554807
H	-1.191959	4.955991	3.110272
H	-3.234441	1.216745	2.489139
H	-4.114355	-4.814587	0.470284
H	-5.762822	-2.691906	0.629675
H	-4.082444	2.932748	-0.817060
H	-5.921559	3.376756	0.811539
H	-6.847590	1.482774	2.148110
H	4.573364	0.914894	3.405155
H	2.476629	2.352042	4.237055
H	7.309667	-1.160090	0.512868

H	6.407885	-3.020240	-0.887645
H	-3.314930	3.689314	2.731502
H	3.949960	-3.495233	-0.834857
K	1.678767	3.099319	-3.379871

Truncated anion of 2: 1,4-activation

85

scf done: -2279.115807

C	-4.164953	3.008936	-1.559263
C	-3.776897	1.674575	-1.569147
C	-4.556045	0.702181	-0.916458
C	-5.736034	1.111670	-0.272941
C	-6.121066	2.450380	-0.282380
C	-5.338998	3.412058	-0.919684
N	-4.141033	-0.626893	-0.900201
B	-2.757852	-1.127747	-0.938088
O	-1.659788	-0.419237	-1.110956
Al	-0.093480	0.360326	-1.054825
O	0.518029	-0.013929	0.544938
B	0.762823	-0.213471	1.825803
N	0.080327	0.350344	2.998153
C	0.690770	-0.171842	4.139476
C	1.669680	-1.036432	3.793123
N	1.785164	-1.095648	2.404700
C	2.628999	-1.969300	1.722212
C	3.033884	-3.188273	2.290771
C	3.863389	-4.057963	1.586536
C	4.298169	-3.739971	0.300959
C	3.897017	-2.528818	-0.266509
C	3.079336	-1.647137	0.430154
H	2.666413	-3.470678	3.279047
H	2.786281	-0.698587	-0.019819
H	-6.337397	0.380943	0.270510
H	-2.860973	1.369935	-2.075605
C	-4.985362	-1.714593	-0.674919
C	-4.261881	-2.853380	-0.599153
N	-2.901480	-2.573684	-0.728076
C	-1.905008	-3.554253	-0.736914
C	-2.178262	-4.861640	-1.170149
C	-1.179556	-5.832747	-1.165920
C	0.112060	-5.520575	-0.746350
C	0.392043	-4.220923	-0.322239
C	-0.602696	-3.248700	-0.308161
H	-3.175728	-5.112006	-1.536876
H	-0.373775	-2.245757	0.051331
C	1.142665	0.126642	-2.688086
C	0.153574	0.804134	-3.583787
C	-0.381742	1.939756	-3.078454
C	0.104596	2.303164	-1.709998
C	1.592789	2.293313	-1.651399
C	2.168485	1.089192	-2.202553
C	2.410913	3.231102	-1.059573
C	3.814289	3.032907	-0.969705
C	4.386474	1.840883	-1.522690

C	3.531662	0.892284	-2.143529
C	5.787275	1.649369	-1.423484
C	6.597827	2.584008	-0.815712
C	6.034921	3.760266	-0.272750
C	4.675099	3.972861	-0.349010
C	-0.930984	1.313084	3.032726
C	-1.870063	1.398233	1.991158
C	-2.874045	2.360305	2.020481
C	-2.978518	3.248727	3.091461
C	-2.055417	3.165652	4.132012
C	-1.037469	2.215480	4.103581
H	-1.817634	0.694267	1.161225
H	-0.298838	2.188554	4.907022
H	0.897221	-6.280331	-0.748493
H	1.396318	-3.955556	0.015217
H	1.542687	-0.826848	-3.051284
H	-0.125808	0.384143	-4.554313
H	-6.065526	-1.607621	-0.614068
H	-1.130840	2.538976	-3.605373
H	3.970339	-0.018898	-2.562767
H	1.977173	4.138158	-0.627388
H	-3.588908	2.405363	1.196241
H	-3.773025	3.998470	3.109965
H	-2.114613	3.859186	4.975689
H	0.359302	0.091118	5.141006
H	2.327947	-1.597246	4.452330
H	7.676027	2.414768	-0.749291
H	6.217438	0.733274	-1.839460
H	-0.366624	3.177845	-1.247405
H	-1.416418	-6.843663	-1.509803
H	4.157872	-5.005209	2.046673
H	4.941643	-4.427218	-0.253274
H	4.230266	-2.253918	-1.270378
H	-4.627925	-3.862620	-0.426749
H	-7.038464	2.744797	0.234708
H	-5.636881	4.463043	-0.914258
H	-3.532872	3.744814	-2.063299
H	6.679228	4.499198	0.211517
H	4.234155	4.879959	0.075299

Truncated anion of 2: 9,10-activation

85

scf done: -2279.136913

C	2.143631	-5.161715	-1.293674
C	0.810910	-4.857705	-1.026288
C	0.458715	-3.617561	-0.469596
C	1.479124	-2.691816	-0.197369
C	2.805986	-2.997995	-0.481873
C	3.150040	-4.235185	-1.027742
H	0.029335	-5.578251	-1.275382
N	-0.874661	-3.306423	-0.188311
B	-1.542224	-1.999913	-0.254261
N	-2.945710	-2.307678	0.049786
C	-3.986180	-1.399217	0.234587
C	-4.012370	-0.201368	-0.499978

C	-5.033446	0.720031	-0.299408
C	-6.054584	0.473814	0.620605
C	-6.037449	-0.717657	1.343876
C	-5.014063	-1.644548	1.159735
H	1.231545	-1.731945	0.255279
H	-3.227749	0.000171	-1.229572
H	-4.989591	-2.553770	1.763398
C	-1.841040	-4.265257	0.119219
C	-3.047029	-3.678521	0.284592
O	-1.027658	-0.808983	-0.499318
Al	-0.002927	0.589480	-0.733593
O	1.030590	0.653315	0.676789
B	1.548837	0.671132	1.890944
N	0.884722	0.884314	3.183378
C	1.854557	0.791847	4.182690
C	3.059649	0.508080	3.641131
N	2.954132	0.443687	2.252139
C	3.992939	0.072602	1.400624
C	5.026687	-0.773139	1.835000
C	6.048068	-1.147738	0.965048
C	6.057594	-0.697872	-0.354240
C	5.030655	0.142815	-0.788401
C	4.011409	0.532117	0.072551
H	5.008516	-1.167117	2.852909
H	3.221823	1.197187	-0.277721
C	0.806914	0.809472	-2.616619
C	-0.525348	0.949770	-3.275391
C	-1.408852	1.821295	-2.581648
C	-0.822778	2.384188	-1.331152
C	0.506577	3.004599	-1.607845
C	1.390169	2.150217	-2.322566
C	-2.710531	2.000353	-3.053453
C	-3.155583	1.306485	-4.182188
C	-2.293122	0.439703	-4.853433
C	-0.980398	0.268416	-4.405001
C	2.690009	2.578925	-2.597585
C	3.132990	3.826614	-2.149739
C	2.270384	4.658254	-1.435606
C	0.959590	4.250583	-1.172369
C	-0.448632	1.222930	3.430810
C	-1.470366	0.756837	2.587458
C	-2.797212	1.098120	2.828587
C	-3.139952	1.895827	3.920910
C	-2.132302	2.355561	4.766692
C	-0.799581	2.031529	4.523903
H	-1.223723	0.109086	1.746595
H	-0.017164	2.430398	5.172615
H	-0.304415	-0.413980	-4.928852
H	-2.643566	-0.108978	-5.732209
H	-4.182226	1.440619	-4.534303
H	-3.384370	2.680073	-2.522649
H	2.392553	-6.133263	-1.730057
H	0.283620	4.900518	-0.608747
H	-1.505540	3.001484	-0.735111
H	4.195014	-4.470448	-1.243110
H	1.489955	0.102357	-3.102408
H	2.619323	5.631365	-1.078669
H	3.364189	1.922365	-3.156399

H	4.158235	4.146646	-2.355689
H	3.579184	-2.258790	-0.261840
H	-2.380269	2.989388	5.622889
H	-3.571348	0.723482	2.155520
H	-1.590977	-5.317446	0.231611
H	-3.994748	-4.155048	0.523984
H	-5.027240	1.644048	-0.882608
H	-6.853154	1.203736	0.772670
H	-6.821546	-0.925830	2.077057
H	4.009259	0.378741	4.154997
H	1.607498	0.906411	5.235359
H	6.836824	-1.815251	1.322816
H	6.854758	-0.999768	-1.037621
H	-4.184900	2.154961	4.107003
H	5.018354	0.513410	-1.816282

Single crystal X-Ray structure determination:

The Rigaku XtaLAB Synergy-DW VHF is equipped with a PhotonJet-R dual wavelength rotating anode and HyPix-Arc 150° detector to collect all crystallographic data. Crystals were prepared with Paratone-N or perfluorinated oil, mounted on MiTeGen Micromount loops, and quench-cooled with an Oxford Cryosystems open flow N2 cooling device.[11, 12] Specific data collection details are in *Table S3-8*. Data processing involved CrysAlisPro: unit cell refinement, SCALE3 ABSPACK inter-frame scaling, merging equivalent reflections, and diffraction pattern processing. Structures were solved using SHELXT and refined with SHELXL in OLEX2.[13-15] Crystallographic data is in supplementary CIF files (2321565, 2321566, 2321567, 2321568, 2322595, 2322556 and 2322558) available free via the Cambridge Crystallographic Data Centre: http://www.ccdc.cam.ac.uk/data_request/cif."

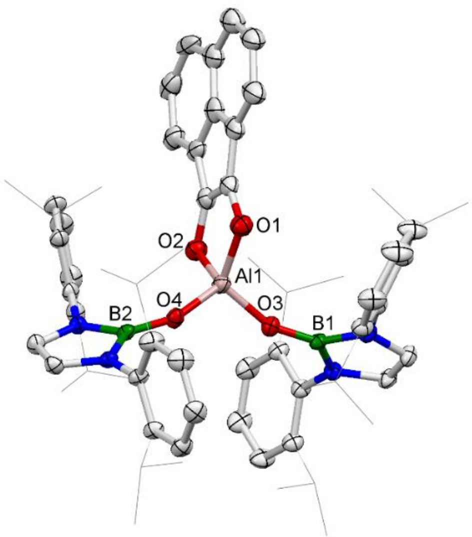

Figure S25: Molecular structure of compound **4** in the solid state as determined by X-ray crystallography (ellipsoids set at 35% probability level; H atoms and counterions omitted and ^{i}Pr groups shown in wireframe format for clarity). Key bond lengths (Å) and angles (°): B1–O3 1.327(5), B2–O4 1.332(5), Al1–O1 1.781(1), Al1–O2 1.838(3), Al1–O3 1.708(3), Al1–O4 1.701(3); O4–Al1–O3 108.8(1).

Table S3: **Crystallographic and refinement parameters for 1**

CCDC Number	2321565
Empirical formula	$C_{52}H_{72}AlB_2KN_4O_2$
Formula weight	872.83
Temperature/K	99.99(10)
Crystal system	monoclinic
Space group	$P2_1/c$
a/Å	10.64203(11)
b/Å	18.2504(2)
c/Å	27.2431(3)
α/°	90
β/°	93.5736(9)
γ/°	90
Volume/$Å^3$	5280.89(10)
Z	4
ρ_{calc}g/cm^3	1.098
μ/mm^{-1}	1.343
F(000)	1880.0
Crystal size/mm^3	0.1 × 0.08 × 0.07
Radiation	Cu Kα (λ = 1.54184)
2Θ range for data collection/°	5.832 to 150.17
Index ranges	$-11 \leq h \leq 13$, $-22 \leq k \leq 21$, $-34 \leq l \leq 32$
Reflections collected	118674
Independent reflections	10743 [R_{int} = 0.0465, R_{sigma} = 0.0174]
Data/restraints/parameters	10743/483/605
Goodness-of-fit on F^2	1.089
Final R indexes [I>=2σ (I)]	R_1 = 0.0847, wR_2 = 0.2525
Final R indexes [all data]	R_1 = 0.0899, wR_2 = 0.2561
Largest diff. peak/hole / e $Å^{-3}$	0.80/-0.50

Table S4: **Crystallographic and refinement parameters for 2**

CCDC number	2321566
Empirical formula	$C_{70}H_{108}AlB_2KN_6O_8$
Formula weight	1249.32
Temperature/K	100.15
Crystal system	monoclinic
Space group	$P2_1$
a/Å	12.6114(3)
b/Å	24.5518(5)
c/Å	12.7182(3)
α/°	90
β/°	111.919(3)
γ/°	90
Volume/Å^3	3653.30(16)
Z	2
ρ_{calc}g/cm^3	1.136
μ/mm^{-1}	0.139
F(000)	1352.0
Crystal size/mm^3	0.83 × 0.16 × 0.06
Radiation	MoKα (λ = 0.71073)
2Θ range for data collection/°	3.318 to 54.968
Index ranges	$-16 \leq h \leq 16$, $-31 \leq k \leq 31$, $-16 \leq l \leq 14$
Reflections collected	69529
Independent reflections	16753 [R_{int} = 0.0400, R_{sigma} = 0.0339]
Data/restraints/parameters	16753/51/861
Goodness-of-fit on F^2	1.032
Final R indexes [I>=2σ (I)]	R_1 = 0.0389, wR_2 = 0.0890
Final R indexes [all data]	R_1 = 0.0460, wR_2 = 0.0916
Largest diff. peak/hole / e Å^{-3}	0.29/-0.22
Flack parameter	0.018(12)

Table S5: **Crystallographic and refinement parameters for 3**

CCDC Number	2321567
Empirical formula	$C_{88}H_{126}AlB_2KN_6O_8$
Formula weight	1483.64
Temperature/K	100.00(10)
Crystal system	triclinic
Space group	P-1
a/Å	12.76230(6)
b/Å	18.06461(10)
c/Å	19.63512(10)
α/°	74.2755(5)
β/°	80.5749(4)
γ/°	78.3274(5)
Volume/$Å^3$	4238.69(4)
Z	2
$\rho_{calc}g/cm^3$	1.162
μ/mm^{-1}	1.093
F(000)	1604.0
Crystal size/mm^3	0.15 × 0.1 × 0.1
Radiation	Cu Kα (λ = 1.54184)
2Θ range for data collection/°	4.706 to 152.186
Index ranges	$-15 \leq h \leq 15$, $-22 \leq k \leq 22$, $-24 \leq l \leq 24$
Reflections collected	198506
Independent reflections	17406 [R_{int} = 0.0348, R_{sigma} = 0.0155]
Data/restraints/parameters	17406/18/1034
Goodness-of-fit on F^2	1.023
Final R indexes [I>=2σ (I)]	R_1 = 0.0385, wR_2 = 0.1018
Final R indexes [all data]	R_1 = 0.0431, wR_2 = 0.1046
Largest diff. peak/hole / e $Å^{-3}$	0.33/-0.30

Table S6: **Crystallographic and refinement parameters for 4**

CCDC Number	2321568
Empirical formula	$C_{82}H_{114}AlB_2KN_6O_{10}$
Formula weight	1431.49
Temperature/K	150.0(3)
Crystal system	monoclinic
Space group	$P2_1/c$
a/Å	13.41780(10)
b/Å	27.0912(2)
c/Å	22.7470(2)
α/°	90
β/°	96.1120(10)
γ/°	90
Volume/Å^3	8221.63(11)
Z	4
ρ_{calc}g/cm^3	1.156
μ/mm^{-1}	1.130
F(000)	3080.0
Crystal size/mm^3	0.5 × 0.1 × 0.1
Radiation	Cu Kα (λ = 1.54184)
2Θ range for data collection/°	6.526 to 152.546
Index ranges	$-16 \leq h \leq 16$, $-30 \leq k \leq 34$, $-27 \leq l \leq 28$
Reflections collected	100118
Independent reflections	17072 [R_{int} = 0.0521, R_{sigma} = 0.0337]
Data/restraints/parameters	17072/54/923
Goodness-of-fit on F^2	1.112
Final R indexes [I>=2σ (I)]	R_1 = 0.0997, wR_2 = 0.2744
Final R indexes [all data]	R_1 = 0.1101, wR_2 = 0.2813
Largest diff. peak/hole / e Å^{-3}	1.62/-0.85

Table S7: **Crystallographic and refinement parameters for 5**

CCDC Number	2322595
Empirical formula	$C_{80}H_{116}AlB_2KN_6O_8$
Formula weight	1377.48
Temperature/K	104(5)
Crystal system	triclinic
Space group	P-1
a/Å	14.20230(10)
b/Å	16.62790(10)
c/Å	20.0099(2)
α/°	79.5050(10)
β/°	72.5670(10)
γ/°	70.6550(10)
Volume/Å^3	4235.76(7)
Z	2
ρ_{calc}g/cm^3	1.080
μ/mm^{-1}	1.060
F(000)	1488.0
Crystal size/mm^3	0.08 × 0.07 × 0.06
Radiation	Cu Kα (λ = 1.54184)
2Θ range for data collection/°	4.648 to 152.116
Index ranges	$-17 \leq h \leq 17, -20 \leq k \leq 20, -25 \leq l \leq 25$
Reflections collected	186128
Independent reflections	17428 [R_{int} = 0.0402, R_{sigma} = 0.0168]
Data/restraints/parameters	17428/691/941
Goodness-of-fit on F^2	1.028
Final R indexes [I>=2σ (I)]	R_1 = 0.0437, wR_2 = 0.1208
Final R indexes [all data]	R_1 = 0.0486, wR_2 = 0.1239
Largest diff. peak/hole / e Å^{-3}	0.29/-0.45

Table S8: **Crystallographic and refinement parameters for 6**

CCDC Number	2322556
Empirical formula	$C_{84}H_{100}AlB_2KN_4O_2$
Formula weight	1285.37
Temperature/K	150.15
Crystal system	monoclinic
Space group	I2
a/Å	12.45390(10)
b/Å	17.3714(2)
c/Å	17.4410(2)
α/°	90
β/°	92.6870(10)
γ/°	90
Volume/Å^3	3769.07(7)
Z	2
ρ_{calc}g/cm^3	1.133
μ/mm^{-1}	1.094
F(000)	1380.0
Crystal size/mm^3	0.22 × 0.22 × 0.2
Radiation	CuKα (λ = 1.54184)
2Θ range for data collection/°	7.186 to 152.538
Index ranges	$-15 \leq h \leq 14$, $-21 \leq k \leq 21$, $-21 \leq l \leq 21$
Reflections collected	49252
Independent reflections	7844 [R_{int} = 0.0325, R_{sigma} = 0.0170]
Data/restraints/parameters	7844/119/481
Goodness-of-fit on F^2	1.047
Final R indexes [I>=2σ (I)]	R_1 = 0.0397, wR_2 = 0.1077
Final R indexes [all data]	R_1 = 0.0405, wR_2 = 0.1085
Largest diff. peak/hole / e Å^{-3}	0.33/-0.24
Flack parameter	0.008(11)

Table S9: **Crystallographic and refinement parameters for 7**

CCDC Number	2322558
Empirical formula	$C_{90}H_{124}AlB_2KN_6O_8$
Formula weight	1505.64
Temperature/K	150.01(10)
Crystal system	monoclinic
Space group	$P2_1/c$
a/Å	15.59330(10)
b/Å	36.9424(2)
c/Å	15.67490(10)
α/°	90
β/°	105.6910(10)
γ/°	90
Volume/Å^3	8693.09(10)
Z	4
ρ_{calc}g/cm^3	1.150
μ/mm^{-1}	1.074
F(000)	3248.0
Crystal size/mm^3	0.25 × 0.23 × 0.17
Radiation	Cu Kα (λ = 1.54184)
2Θ range for data collection/°	7.488 to 152.686
Index ranges	-19 ≤ h ≤ 19, -44 ≤ k ≤ 46, -19 ≤ l ≤ 19
Reflections collected	171246
Independent reflections	18144 [R_{int} = 0.0358, R_{sigma} = 0.0148]
Data/restraints/parameters	18144/61/1033
Goodness-of-fit on F^2	1.065
Final R indexes [I>=2σ (I)]	R_1 = 0.0401, wR_2 = 0.0970
Final R indexes [all data]	R_1 = 0.0472, wR_2 = 0.1023
Largest diff. peak/hole / e Å^{-3}	0.26/-0.25

References:

1. Loh, Y. K.; Ying, L.; Ángeles Fuentes, M.; Do, D. C. H.; Aldridge, S., An N-Heterocyclic Boryloxy Ligand Isoelectronic with N-Heterocyclic Imines: Access to an Acyclic Dioxysilylene and its Heavier Congeners. *Angew. Chem. Int. Ed.* **2019,** *58* (15), 4847-4851.
2. Ganesamoorthy, C.; Loerke, S.; Gemel, C.; Jerabek, P.; Winter, M.; Frenking, G.; Fischer, R. A., Reductive elimination: a pathway to low-valent aluminium species. *Chem. Commun.* **2013,** *49* (28), 2858-2860.
3. Gaussian 16, R. C., M. J. Frisch, G. W. Trucks, H. B. Schlegel, G. E. Scuseria, M. A. Robb, J. R. Cheeseman, G. Scalmani, V. Barone, G. A. Petersson, H. Nakatsuji, X. Li, M. Caricato, A. V. Marenich, J. Bloino, B. G. Janesko, R. Gomperts, B. Mennucci, H. P. Hratchian, J. V. Ortiz, A. F. Izmaylov, J. L. Sonnenberg, D. Williams-Young, F. Ding, F. Lipparini, F. Egidi, J. Goings, B. Peng, A. Petrone, T. Henderson, D. Ranasinghe, V. G. Zakrzewski, J. Gao, N. Rega, G. Zheng, W. Liang, M. Hada, M. Ehara, K. Toyota, R. Fukuda, J. Hasegawa, M. Ishida, T. Nakajima, Y. Honda, O. Kitao, H. Nakai, T. Vreven, K. Throssell, J. A. Montgomery, Jr., J. E. Peralta, F. Ogliaro, M. J. Bearpark, J. J. Heyd, E. N. Brothers, K. N. Kudin, V. N. Staroverov, T. A. Keith, R. Kobayashi, J. Normand, K. Raghavachari, A. P. Rendell, J. C. Burant, S. S. Iyengar, J. Tomasi, M. Cossi, J. M. Millam, M. Klene, C. Adamo, R. Cammi, J. W. Ochterski, R. L. Martin, K. Morokuma, O. Farkas, J. B. Foresman, D. J. Fox *Gaussian, Inc., Wallingford CT* **2019**.

4. Perdew, J. P.; Burke, K.; Ernzerhof, M., Generalized Gradient Approximation Made Simple. *Phys. Rev. Lett.* **1996,** *77* (18), 3865-3868.
5. Perdew, J. P.; Burke, K.; Ernzerhof, M., Generalized Gradient Approximation Made Simple. *Phys. Rev. Lett.* **1997,** *78* (7), 1396-1396.
6. Adamo, C.; Barone, V., Toward reliable density functional methods without adjustable parameters: The PBE0 model. *J. Chem. Phys.* **1999,** *110* (13), 6158-6170.
7. Weigend, F.; Ahlrichs, R., Balanced basis sets of split valence, triple zeta valence and quadruple zeta valence quality for H to Rn: Design and assessment of accuracy. *Phys. Chem. Chem. Phys.* **2005,** *7* (18), 3297-3305.
8. Weigend, F., Accurate Coulomb-fitting basis sets for H to Rn. *Phys. Chem. Chem. Phys.* **2006,** *8* (9), 1057-1065.
9. Grimme, S.; Ehrlich, S.; Goerigk, L., Effect of the damping function in dispersion corrected density functional theory. *J. Comp. Chem.* **2011,** *32* (7), 1456-1465.
10. Marenich, A. V.; Cramer, C. J.; Truhlar, D. G., Universal Solvation Model Based on Solute Electron Density and on a Continuum Model of the Solvent Defined by the Bulk Dielectric Constant and Atomic Surface Tensions. *J. Phys. Chem. B* **2009,** *113* (18), 6378-6396.
11. Cosier, J.; Glazer, A. M. A nitrogen-gas-stream cryostat for general X-ray diffraction studies. *J. Appl. Cryst.* **1986**, 19, 105.
12. CrysAlisPro, Agilent Technologies, Version 1.171.39.46.
13. Sheldrick, G. M. SHELXT - Integrated space-group and crystal-structure determination. *Acta Crystallogr., Sect. A: Found. Adv.* **2015**, 71, 3.
14. Sheldrick, G. M. Crystal structure refinement with SHELXL. *Acta Crystallogr., Sect. C: Struct. Chem*. **2015**, 71, 3.
15. Dolomanov, O. V.; Bourhis, L. J.; Gildea, R. J.; Howard, J. A. K.; Puschmann, H. OLEX2: a complete structure solution, refinement and analysis program. *J. Appl. Cryst.* **2009**, 42, 339.
